# Gut-disc axis: A Mendelian randomization study on the relationship between gut microbiota and cervical spondylosis

**DOI:** 10.1097/MD.0000000000041536

**Published:** 2025-02-14

**Authors:** Jiling Zhang, Baodong Wang, Peng Du, He Song, Lihui Yang, Yu Zhou

**Affiliations:** a Department of Clinical Laboratory, Beijing Shunyi District Hospital, Beijing, China; b Department of Orthopedics, Beijing Chaoyang Hospital, Capital Medical University, Beijing, China.

**Keywords:** cervical spondylosis, gut microbiota, gut-disc axis, Mendelian randomization

## Abstract

The gut-disc axis, which refers to the interaction between gut microbiota and bone health, has recently garnered widespread attention in the scientific community. However, it remains to be determined whether gut microbiota directly induces cervical spondylosis (CS). This study employed a bidirectional 2-sample Mendelian randomization (MR) analysis to explore the potential causal link between gut microbiota and CS. We initially used the inverse variance weighted method for preliminary estimation and supplemented it with other MR methods, including MR-Egger, weighted median, weighted mode, and simple mode. Furthermore, we utilized the Cochrane *Q* test, MR-PRESSO global test, and MR-Egger intercept test to assess possible pleiotropy and heterogeneity. Ultimately, we conducted a bidirectional MR study to investigate potential reverse associations between gut microbiota and CS. The preliminary MR analysis identified 27 gut microbiota significantly associated with CS, of which 12 may be contributing factors, while 15 may have protective effects. The reverse MR analysis further revealed a potential causal relationship between CS and 24 gut microbiota. In this study, no significant heterogeneity or pleiotropy was detected. Through MR analysis, we uncovered a significant causal relationship between gut microbiota and CS, providing new perspectives for the prevention and treatment of CS, especially in the modulation of the microbiota.

## 1. Introduction

Cervical spondylosis (CS) is a degenerative disease characterized by neck pain, dizziness, myelopathy or structural changes, and even loss of horizontal gaze.^[1,2]^ In 2017, it was reported that 288.7 million patients worldwide suffered from neck pain, with 28.6 million being disabled as a result.^[3]^ CS not only disrupts patients’ daily life, significantly reducing their quality of life, but also triggers psychosocial issues.^[4]^ Presently, the exact cause is still unclear, with related factors including degeneration, trauma, strain, and congenital deformity.^[5]^

The gut microbiota, often called our “second genome,” plays a key role in various histological functions, including metabolism, immune defense, infection prevention, regulation of intestinal barrier structure integrity, and regulation of the nervous system.^[6,7]^ Recently, the emerging paradigm of the gut-bone axis and gut-disc axis has gained attention, revealing the microbiota’s role in digestion, metabolism, immune function, and its influence on bone metabolism through different mechanisms.^[8]^ Some researchers have found that some protective bacteria are more common in normal discs, while harmful bacteria are prevalent in degenerative discs.^[9]^ Other studies have shown that gut microbiota, particularly those from the Burkholderiales order, can increase osteoclasts and reduce the risk of postmenopausal osteoporosis.^[10]^ In addition, Fang identified 46 taxa and 33 metabolic pathways related to intervertebral disc degeneration, low back pain, and sciatica.^[8]^ Despite these findings, the exact relationship between gut microbiota and CS is still unclear, highlighting the need to further investigate the connection between the 2.

Mendelian randomization (MR) uses genetic variation as tool to assess the causal effect of exposure on outcomes. It mitigates some inherent issues in observational studies, such as confounding and reverse causality, by leveraging the random allocation of genetic variants, providing more reliable causal inferences.^[11]^ Due to its scientific rigor and cost-effectiveness, MR has been used in studying various diseases, including CS.^[12]^ In this study, we used aggregated data from large-scale genome-wide association studies (GWAS) and MR analysis to explore the causal relationship between gut microbiota and CS, aiming to provide more insights for preventing and managing CS.

## 2. Materials and methods

### 2.1. Study design

**Figure 1.**
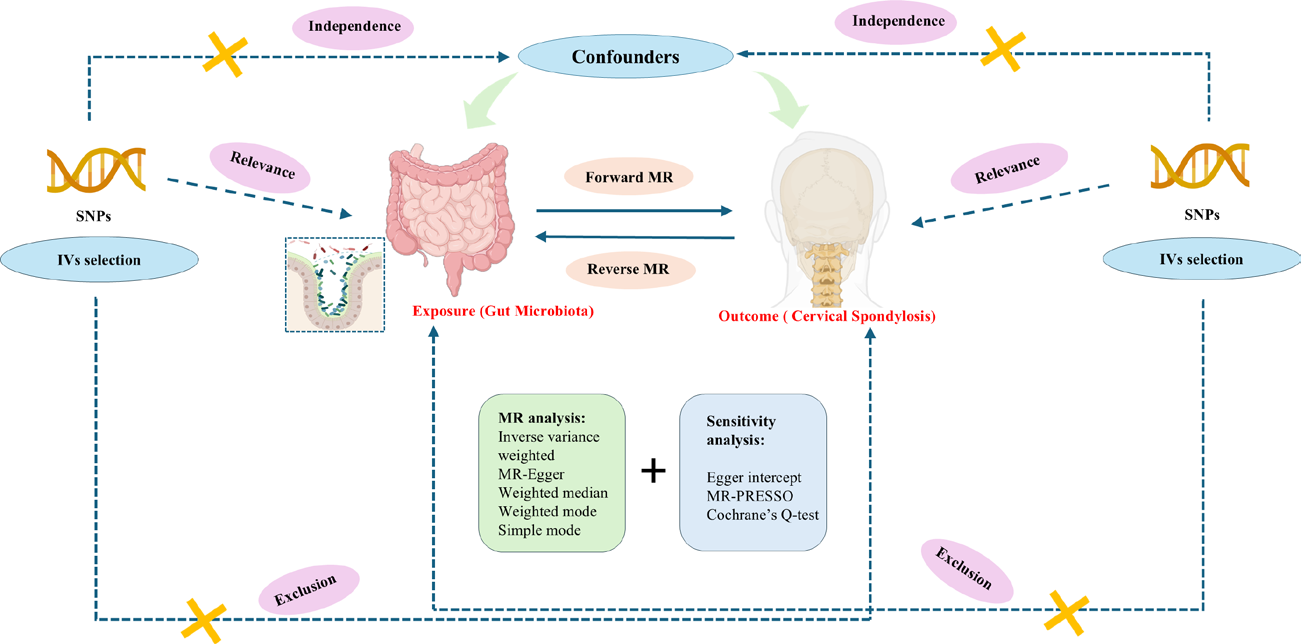
Research design for bidirectional MR study of the association between gut microbiota and cervical spondylosis. MR = Mendelian randomization, SNPs = single nucleotide polymorphisms.

Figure 1 illustrates the schematic of this study. To determine the causal relationship between 473 gut microbial groups and CS, we used a 2-sample bidirectional MR study. Genetic variants served as instrumental variables (IVs) to obtain causal relationships.^[13]^ Therefore, all IVs must satisfy 3 assumptions: IVs are significantly correlated with exposure; IVs affected the outcome only through exposure; IVs should not have a direct effect on the outcome.

### 2.2. Data selection

#### 2.2.1. Gut microbiota GWAS

Comprehensive, aggregated statistics on gut microbiota with key genome-wide findings are publicly available in the NHGRI-EBI GWAS catalog (accession IDs GCST90032172 to GCST90032644). Genetic variants associated with the gut microbiome were obtained from genome-wide association tests involving 7,979,834 genetic variants in 5959 individuals from the FR02 cohort, which is based on the Finnish population.^[14]^ The comparison table of intestinal microbes is shown in Table S1, Supplemental Digital Content, http://links.lww.com/MD/O381.

#### 2.2.2. Cervical spondylosis GWAS

We obtained cervical spondylopathy data from the FinnGen database (https://www.finngen.fi/en). We can access the FinnGen project on the website, which provides detailed participants information.

#### 2.2.3. Qualified IV selection

To ensure accurate and reliable results, we have implemented quality control measures to identify important independent IVs for each gut microbiome and cervical spine disease. The procedure for selecting IVs is as follows: select single nucleotide polymorphisms (SNPs) with *P*-values below the genome-wide significance threshold (5 × 10^−6^).^[15]^ To prevent bias in MR analysis, it is necessary to remove linkage disequilibrium and ensure SNPs are independent. The parameters are set as follows: *r*^2^ = 0.001 and kb = 10,000.^[16]^ When an SNP is missing, we look for a proxy SNP with linkage disequilibrium *r*^2^ > 0.8. If no suitable proxy is found, the SNP is discarded. Coordinate SNPs of exposure and results, and remove palindromic sequences. Calculate the F statistic for each gut microbiota to prevent weak IVs (*F* < 10) from violating the association hypothesis and reducing test power. *F*-value is calculated by the formula *F* = *R*^2^ (*N* − *k* − 1)/ *k* (1 − *R*^2^). Here, *R*^2^ is the proportion of variance explained by all SNPs, *N* is the total sample size, and *k* is the number of SNPs.^[17]^

### 2.3. MR Analysis and sensitivity analysis

In our MR study, we used the inverse variance weighted (IVW) method as the primary analysis approach, supplemented by MR-Egger, weighted median, weighted mode, and simple mode methods. In the IVW method, we used a fixed-effects meta-analysis model, which is reliable because it combines the causal effects of each SNP. Previous MR studies of the gut microbiota suggested that *P < *.05 signifies a significant association between exposure and outcomes.^[18]^ We also conducted a comprehensive sensitivity analysis and detected abnormal deviations using MR-PRESSO. Heterogeneity between each SNP estimate was assessed using Cochrane’s *Q* test. The MR-Egger intercept was used to check for horizontal pleiotropy. Reservation-sensitivity analysis was used to prevent significant changes in overall estimates due to strong SNP effects.^[19,20]^ All analyses were conducted using software (version 4.3.3). We used the “Two Sample MR” software package (version 0.5.7) for MR and MR-PRESSO analyses.

### 2.4. Reverse Mendelian randomization analysis

To further evaluate the causal relationship between gut microbiota and CS, we conducted reverse MR analysis, with CS as the exposure and gut microbiota as the outcome. We selected SNPs strongly associated with the exposure with a significance threshold of *P < *5 × 10^−8^, and all other methods and settings were consistent with those used in the forward analysis.

### 2.5. Ethical approval

The dataset used in this study is publicly available through GWAS. Since the data we used come from studies with ethical approval from the committee that oversees standards for human experimentation, no additional ethical approval was required for this study.

## 3. Results

### 3.1. The causal effects of gut microbiota on cervical spondylosis

**Figure 2.**
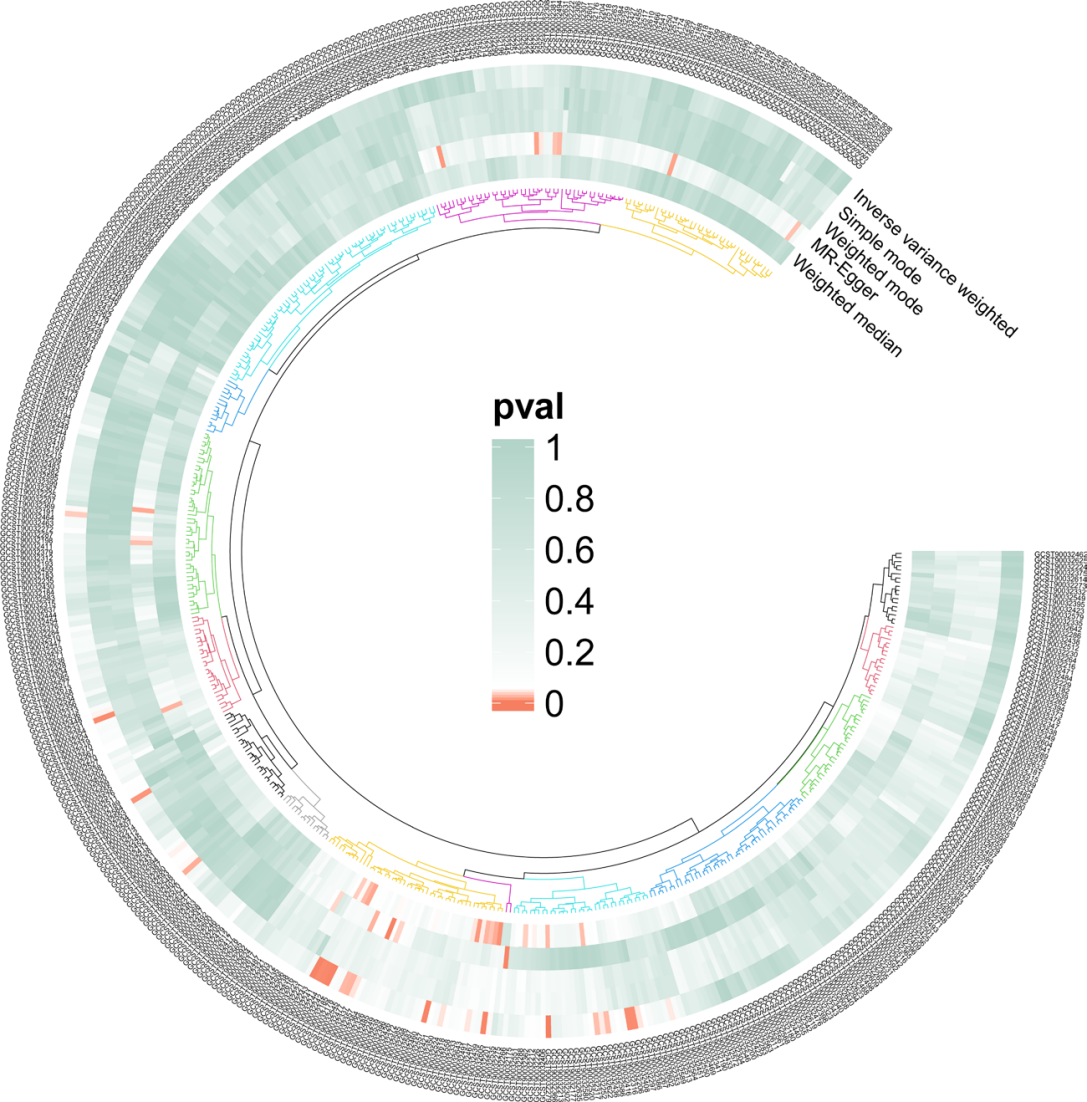
Heatmap outcomes of the causal link between gut microbiota and cervical spondylosis.

**Figure 3.**
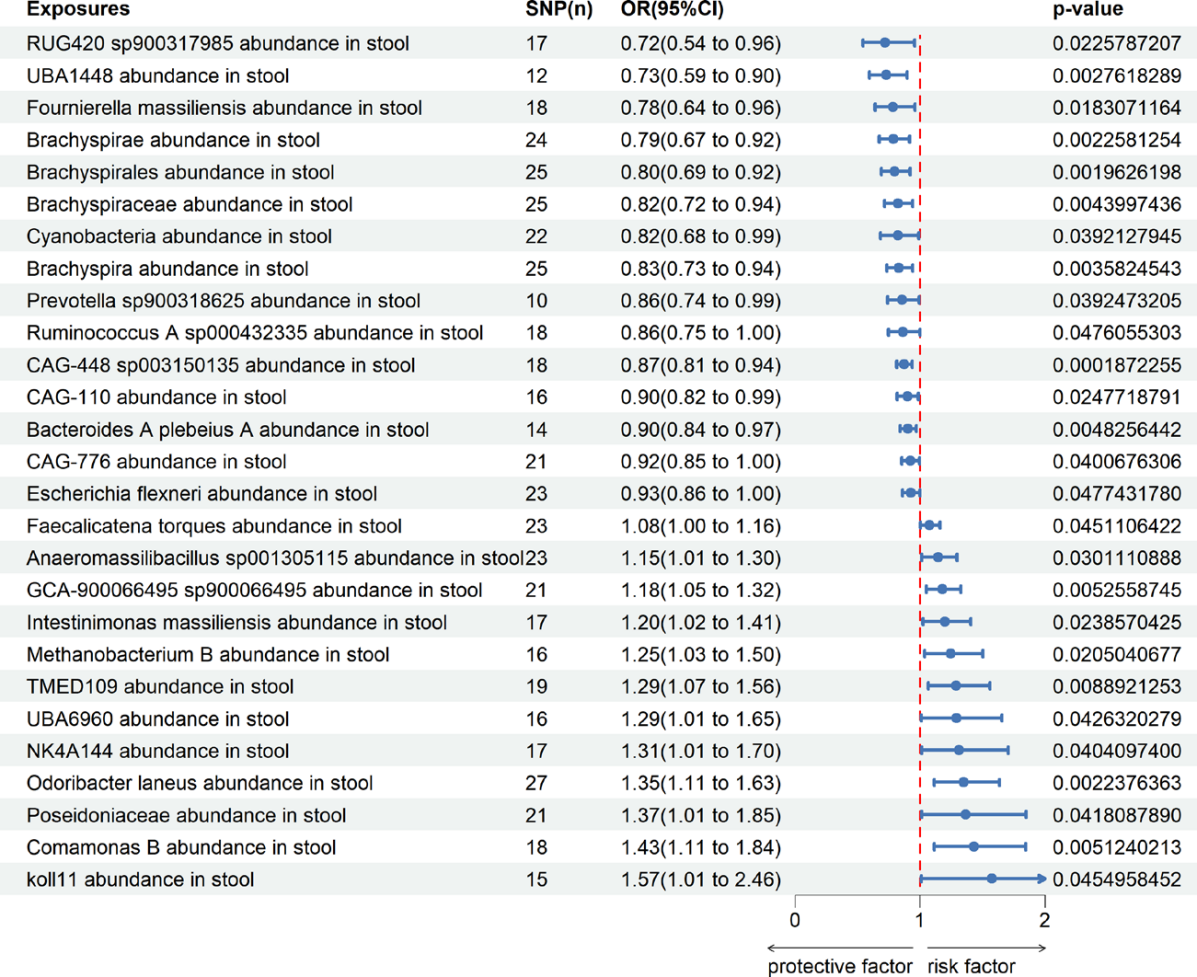
The forest plot demonstrates a correlation between the gut microbiome and cervical spondylosis. SNP (n) = number of single nucleotide polymorphism, OR = odds ratio, CI = confidence interval.

We created a circus plot to visually represent the data (Fig. 2), including related bacterial taxa (GTDB 89th Edition nomenclature). Five methods were used, with the IVW method being the main. The forward MR analysis identified significant associations between 27 microbial taxa and cervical spine disease. Preliminary results are shown in Table S2, Supplemental Digital Content, http://links.lww.com/MD/O381. Fifteen microbial taxa were found to have a protective effect, while 12 increase the risk of cervical spine disease, IVW results as shown in Figure 3. The microbial taxa that play a protective role in cervical spine disease are as follows: RUG420 sp900317985 abundance in stool (OR = 0.721, 95% CI = 0.544–0.955; *P* = .023), UBA1448 abundance in stool (OR = 0.729, 95% CI = 0.593–0.897; *P* = .003), *Fournierella massiliensis* abundance in stool (OR = 0.784, 95% CI = 0.641–0.960; *P* = .018), Brachyspirae abundance in stool (OR = 0.785, 95% CI = 0.673–0.917; *P* = .002), Brachyspirales abundance in stool (OR = 0.796, 95% CI = 0.689–0.920; *P* = .002), Brachyspiraceae abundance in stool (OR = 0.822, 95% CI = 0.718–0.941; *P* = .004), Cyanobacteria abundance in stool (OR = 0.823, 95% CI = 0.683–0.990; *P* = .039), Brachyspira abundance in stool (OR = 0.830, 95% CI = 0.733–0.941; *P* = .004), Prevotella sp900318625 abundance in stool (OR = 0.857, 95% CI = 0.741–0.992; *P* = .039), Ruminococcus A sp000432335 abundance in stool (OR = 0.863, 95% CI = 0.745–0.998; *P* = .048), CAG-448 sp003150135 abundance in stool (OR = 0.872, 95% CI = 0.812–0.937; *P* = .000), CAG-110 abundance in stool (OR = 0.898, 95% CI = 0.818–0.986; *P* = .025), Bacteroides A plebeius A abundance in stool (OR = 0.902, 95% CI = 0.840–0.969; *P* = .005), CAG-776 abundance in stool (OR = 0.923, 95% CI = 0.854–0.996; *P* = .040), *Escherichia flexneri* abundance in stool (OR = 0.927, 95% CI = 0.861–0.999; *P* = .048).

In addition, the microbial taxa that increase CS risk are as follows: Faecalicatena torques abundance in stool (OR = 1.077, 95% CI = 1.077–1.159; *P* = .045), Anaeromassilibacillus sp001305115 abundance in stool (OR = 1.146, 95% CI = 1.013–1.296; *P* = .030), GCA-900066495 sp900066495 abundance in stool (OR = 1.180, 95% CI = 1.050–1.325; *P* = .005), *Intestinimonas massiliensis* abundance in stool (OR = 1.200, 95% CI = 1.024–1.406; *P* = .024), Methanobacterium B abundance in stool (OR = 1.247, 95% CI = 1.035–1.503; *P* = .021), TMED109 abundance in stool (OR = 1.289, 95% CI = 1.066–1.559; *P* = .009), UBA6960 abundance in stool (OR = 1.292, 95% CI = 1.009–1.655; *P* = .043), NK4A144 abundance in stool (OR = 1.313, 95% CI = 1.012–1.704; *P* = .040), *Odoribacter laneus* abundance in stool (OR = 1.349, 95% CI = 1.113–1.634; *P* = .002), Poseidoniaceae abundance in stool (OR = 1.367, 95% CI = 1.012–1.848; *P* = .042), Comamonas B abundance in stool (OR = 1.433, 95% CI = 1.114–1.844; *P* = .005), koll11 abundance in stool (OR = 1.574, 95% CI = 1.009–2.456; *P* = .045).

Additionally, we conducted sensitivity analyses. Cochrane’s *Q* test and MR-Egger regression intercept analysis indicated no significant heterogeneity or horizontal pleiotropy among the IVs. MR-PRESSO analysis found no outliers, as shown in Table 1. Finally, we visualized the results and confirmed them using a funnel plot and the leave-one-out method, as shown in Figures S1 and S2, Supplemental Digital Content, http://links.lww.com/MD/O381.

**Table 1:** Sensitivity analysis of the causal association between gut microbiota and cervical spondylosis.

Gut microbiota	Heterogeneity		MR_PRESSO			Pleiotropy	
	Method	*Q*	Q_pval	MR_PRESSO outlier-corrected	MR_PRESSO global test *P*-value	MR_Egger_intercept	*P*-value
Protective factor							
RUG420 sp900317985 abundance in stool	MR_Egger	19.332	0.199	NA	.126	0.021	.099
	IVW	23.320	0.105				
UBA1448 abundance in stool	MR_Egger	2.387	0.992	NA	.993	0.007	.617
	IVW	2.654	0.995				
*Fournierella massiliensis* abundance in stool	MR_Egger	19.198	0.259	NA	.343	-0.004	.677
	IVW	19.414	0.305				
Brachyspirae abundance in stool	MR_Egger	20.148	0.574	NA	.660	0.001	.902
	IVW	20.164	0.632				
Brachyspirales abundance in stool	MR_Egger	19.233	0.688	NA	.729	0.002	.848
	IVW	19.270	0.737				
Brachyspiraceae abundance in stool	MR_Egger	18.851	0.710	NA	.764	0.001	.875
	IVW	18.877	0.759				
Cyanobacteria abundance in stool	MR_Egger	14.452	0.807	NA	.816	0.009	.290
	IVW	15.631	0.790				
Brachyspira abundance in stool	MR_Egger	23.117	0.454	NA	.542	0.002	.839
	IVW	23.159	0.510				
Prevotella sp900318625 abundance in stool	MR_Egger	3.036	0.932	NA	.951	0.006	.731
	IVW	3.163	0.957				
Ruminococcus A sp000432335 abundance in stool	MR_Egger	19.627	0.237	NA	.293	−0.002	.920
	IVW	19.640	0.293				
CAG-448 sp003150135 abundance in stool	MR_Egger	7.306	0.967	NA	.919	−0.014	.124
	IVW	9.947	0.906				
CAG-110 abundance in stool	MR_Egger	9.941	0.767	NA	.715	−0.012	.325
	IVW	10.983	0.754				
Bacteroides A plebeius A abundance in stool	MR_Egger	7.428	0.828	NA	.875	−0.010	.473
	IVW	7.976	0.845				
CAG-776 abundance in stool	MR_Egger	20.268	0.379	NA	.373	−0.011	.272
	IVW	21.635	0.361				
Escherichia flexneri abundance in stool	MR_Egger	26.705	0.181	NA	.202	0.009	.460
	IVW	27.426	0.195				
Risk factor							
Faecalicatena torques abundance in stool	MR_Egger	19.904	0.527	NA	.564	0.006	.524
	IVW	20.325	0.563				
Anaeromassilibacillus sp001305115 abundance in stool	MR_Egger	24.307	0.278	NA	.338	0.000	.986
	IVW	24.307	0.331				
GCA-900066495 sp900066495 abundance in stool	MR_Egger	17.139	0.580	NA	.555	0.013	.197
	IVW	18.925	0.527				
*Intestinimonas massiliensis* abundance in stool	MR_Egger	13.727	0.546	NA	.569	0.012	.353
	IVW	14.644	0.551				
Methanobacterium B abundance in stool	MR_Egger	10.205	0.747	NA	.706	−0.015	.223
	IVW	11.828	0.692				
TMED109 abundance in stool	MR_Egger	18.394	0.364	NA	.410	0.009	.481
	IVW	18.955	0.395				
UBA6960 abundance in stool	MR_Egger	7.562	0.911	NA	.953	0.009	.493
	IVW	8.057	0.921				
NK4A144 abundance in stool	MR_Egger	13.619	0.555	NA	.627	−0.005	.660
	IVW	13.821	0.612				
*Odoribacter laneus* abundance in stool	MR_Egger	28.045	0.306	NA	.350	0.003	.763
	IVW	28.150	0.351				
Poseidoniaceae abundance in stool	MR_Egger	14.632	0.746	NA	.797	0.002	.866
	IVW	14.662	0.795				
Comamonas B abundance in stool	MR_Egger	18.428	0.299	NA	.402	−0.002	.842
	IVW	18.475	0.359				
koll11 abundance in stool	MR_Egger	9.640	0.723	NA	.756	−0.004	.661
	IVW	9.841	0.774				

IVW = inverse variance weighted.

### 3.2. The causal effects of cervical spondylosis on gut microbiota

To assess the reverse causal effect on 473 gut microbiota, we performed a 2-sample reverse MR analysis, as shown in Table S3, Supplemental Digital Content, http://links.lww.com/MD/O381. This analysis identified potential causal relationships between CS and 24 gut microbial taxa. However, these causal relationships were not highly significant, specifically the following: Agathobacter sp000434275 abundance in stool (OR = 0.930, 95% CI = 0.887–0.975; *P* = .002), Ruminococcus D abundance in stool (OR = 0.950, 95% CI = 0.906–0.995; *P* = .030), Ruminococcus D bicirculans abundance in stool (OR = 0.952, 95% CI = 0.909–0.998; *P* = .040), Negativibacillus sp000435195 abundance in stool (OR = 0.959, 95% CI = 0.922–0.997; *P* = .035), *Lactococcus lactis* abundance in stool (OR = 0.970, 95% CI = 0.944–0.998; *P* = .034), UBA7177 abundance in stool (OR = 0.975, 95% CI = 0.955–0.995; *P* = .013), Faecalicatena sp002161355 abundance in stool (OR = 0.975, 95% CI = 0.952–0.998; *P* = .032), Hungatella sp900155545 abundance in stool (OR = 0.978, 95% CI = 0.961–0.995; *P* = .011), UBA7177 sp002491225 abundance in stool (OR = 0.981, 95% CI = 0.963–0.999; *P* = .037), UBA7182 abundance in stool (OR = 0.981, 95% CI = 0.965–0.998; *P* = .028), Morganella abundance in stool (OR = 0.982, 95% CI = 0.965–0.998; *P* = .032), UBA7182 sp002491115 abundance in stool (OR = 0.983, 95% CI = 0.969–0.997; *P* = .015), Kineothrix abundance in stool (OR = 1.433, 95% CI = 1.114–1.844; *P* = .005) (OR = 0.984, 95% CI = 0.969–0.998; *P* = .024), Thermoplasmatota abundance in stool (OR = 1.013, 95% CI = 1.002–1.024; *P* = .022), Stappia abundance in stool (OR = 1.014, 95% CI = 1.001–1.027; *P* = .038), *Enorma massiliensis* abundance in stool (OR = 1.023, 95% CI = 1.000–1.047; *P* = .045), *Eubacterium callanderi* abundance in stool (OR = 1.024, 95% CI = 1.004–1.044; *P* = .016), CAG-841 sp002479075 abundance in stool (OR = 1.025, 95% CI = 1.004–1.0478; *P* = .019), CAG-698 abundance in stool (OR = 1.027, 95% CI = 1.001–1.053; *P* = .041), Prevotella sp900318625 abundance in stool (OR = 1.029, 95% CI = 1.000–1.058; *P* = .048), CAG-485 sp002404675 abundance in stool (OR = 1.030, 95% CI = 1.006–1.054; *P* = .015), CAG-884 sp000433875 abundance in stool (OR = 1.034, 95% CI = 1.001–1.068; *P* = .046), CAG-273 sp003507395 abundance in stool (OR = 1.0534, 95% CI = 1.003–1.106; *P* = .037), CAG-349 abundance in stool (OR = 1.084, 95% CI = 1.018–1.153; *P* = .011). In addition, no horizontal pleiotropy or heterogeneity was found, as shown in Table S4, Supplemental Digital Content, http://links.lww.com/MD/O381.

## 4. Discussion

The gut microbiota is a complex ecosystem in the human gut, composed of a variety of microorganisms, including bacteria, fungi, viruses, and more. Recent studies have shown that the gut microbiome composition regulates the interaction between immune and dendritic cells, producing molecules such as short-chain fatty acids, indole derivatives, polyamines, and secondary bile acids, enabling their receptors on immune cells and regulating immune cell differentiation.^[21]^ In addition, the close interaction between immune and bone cells allows the gut microbiome to play a central role in bone health.^[22]^ For instance, studies have shown significant differences in gut microbiota abundance between patients with osteoporosis or reduced bone mass and the control group.^[23]^ Beneficial changes gut microbiota composition can improve bone metabolism as people age. A genome-wide association study using MR demonstrated that Burkella is associated with an increase in osteoclasts and a reduced risk of postmenopausal osteoporosis.^[10]^ Additionally, there is a strong causal relationship between gut microbiota and bone mineral density throughout life, with Firmicutes being the main group associated with bone mineral density. For individuals aged 45 and older, the number of gut microbiota species related to bone density increases.^[24]^ The state of the gut microbiota affects the skeletal system, and vice versa. This interaction forms the gut-bone axis, which is crucial for balancing the body’s immune system and maintaining bone health.^[25]^

Later, with the in-depth study of gut microbiota and bone diseases, the scientific research team proposed the concept of “gut-disc axis” and elaborated the 3 potential mechanisms of gut microbiota on the intervertebral disc, namely, bacteria across the intestinal epithelial barrier, the role of mucosal and systemic immune system, and the role of nutrient absorption and metabolite formation and diffusion of the intestinal epithelium.^[26]^ This suggests that the crosstalk mechanisms among gut microbiota may significantly impact intervertebral disc–related diseases. It has been proven that the bacterium *Faecalibacterium prausnitzii* has a protective effect against intervertebral disc degeneration, and its mechanism may be related to cholesterol lipid metabolism.^[8]^ The study found that Prevotella and Lactobacillus S16 may reduce the abnormal inflammatory response of lumbar disc herniation and alleviate low back pain, by reducing the percentage of Th1 + Th2 cells and Th17/Treg ratio, inhibiting the expression of pro-inflammatory cytokines.^[27]^ The genus Allisonella is positively correlated with bone turnover markers, serum N-terminal propeptide of type I procollagen (P1NP), and C-terminal telopeptide of type I collagen (CTX-1). The gut microbiota can stimulate the calcium and vitamin D absorption, which could contribute to sciatica.^[28]^

Cervical spondylosis, a type of degenerative disc disease, should not be taken lightly in terms of its severity. It can weaken muscle strength and cause muscle atrophy in the upper limbs, cause eye pain and blurred vision, and in severe cases lead to blindness. Moreover, it can impair blood supply to vital arteries such as the posterior cerebral artery, the inferior cerebellar artery, and the inner ear artery. Full recovery is often elusive for those affected.^[29]^ Currently, it is believed that approximately 349 million individuals worldwide suffer from neck pain and related conditions.^[30]^ Exploring the role of the gut microbiome in cervical disc disease, or CS, may pave the way for new preventive and therapeutic approaches. To our knowledge, this is the first MR analysis to examine the causal relationship between gut microbes and CS. Using the most comprehensive microbiome GWAS data available, we examined 473 different microbial taxa and confirmed that 27 specific gut microbiome taxa have a causal effect on CS. Our reverse MR analysis also suggested potential causal relationships between CS and 24 gut microbial taxa, although the associations were not highly significant. This provides new insights into the possible pathogenesis of CS.

A microcystis cyanobacteria toxin can increase micronucleus frequency in mouse bone marrow erythrocytes and reduce the polychromophilic/orthochromatin ratio in a dose-dependent manner, suggesting that it plays a protective role in bone disease.^[31]^ Only 2 species of Campylobacter have been isolated from humans: Campylobacter and *Campylobacter upsaliensis*. Patients infected with Campylobacter may present with diarrhea, fever, HIV, and immunodeficiency, and are considered pathogenic.^[32]^ While this contrasts with our results to some extent, the research on this bacterium is still limited. On one hand, Prevotella has been associated with rheumatoid arthritis, with the exact pathways still being unclear.^[33]^ On the other hand, the proportion of Prevotella in the osteoporosis group was significantly lower than that in the normal bone mass group, indicating its potential protective effect against osteoporosis.^[34]^

Research indicates that the abundance of g_Ruminococcus is positively correlated with bone resorption factors and intestinal inflammatory factors, while it is negatively correlated with bone formation factors, intestinal barrier indicators, and bone volume fraction.^[35]^ Nosal believes that Ruminococcus 2 may be a key bacterium for its protective effect on bones.^[36]^ There is a growing evidence that Bacteroides have the capacity to mitigate bone loss after menopause.^[37]^ Bacteroidetes also significantly influence skeletal health in male mice and are capable of expanding the Treg cell community in the gastrointestinal tract.^[38]^

Our results also indicate that some gut microbes act as danger signals. For instance, an upsurge in Anaeromassilibacillus can suppress the MEK/ERK pathway, which is pivotal for the transport of immunoglobulin A, causing decreased IgA production. This leads to the breakdown of tight junction proteins in the intestinal epithelium, causing imbalances in the gut microbiota and worsening liver inflammation. This implies that Anaeromassilibacillus might impact CS through immune-related factors.^[39]^ *Odoribacter laneus* is more abundant in participants with type 2 diabetes, indicating its pathogenicity.^[40]^ However, because of the intricate diversity of the gut microbiota, comprehensive studies are still limited. Thus, the full extent of the relationship between the gut microbiota and CS remains to be uncovered.

This study offers several advantages: initially, the MR technique was applied to dissect the causal relationship between the gut microbiota and CS, bypassing confounding variables and reverse causality problems. Moreover, compared with randomized controlled trials, MR research is more time-efficient and is less susceptible to reverse causality and confounding. Lastly, this research utilized the largest available gut microbiome GWAS database, enhancing the comprehensiveness and reliability of the results.

Admittedly, there are some limitations. Initially, the data on gut microbiota and CS are derived from European populations, which restricts the broad applicability of our findings. Future studies are essential to expand the GWAS database. Moreover, while our study suggests potential causal relationships between the gut microbiota and CS, further research is needed to explore the detailed mechanisms, which would involve basic experimental studies. Lastly, the gut microbiota consists of bacteria, fungi, viruses, and other microorganisms. Our study focuses only on gut bacteria in relation to CS. Furthermore, there is limited understanding of how genetics affect the microbial composition of body parts beyond the gut, which limits the scope of this study.

## 5. Conclusion

In conclusion, this study has outlined the causal impact of 27 gut microbes on CS, enhancing the its etiological evidence and presenting innovative approaches for prevention and intervention. Nevertheless, further research is needed to delineate the precise mechanisms through which these microbial groups affect CS.

## Acknowledgments

We express our gratitude to the individuals and scientists involved in the FinnGen study, and we appreciate the MiBioGen Consortium for providing the summary statistics of the gut microbiome GWAS.

## Author contributions

**Conceptualization:** Yu Zhou.

**Data curation:** Jiling Zhang, Baodong Wang, Yu Zhou.

**Formal analysis:** Jiling Zhang, Baodong Wang.

**Investigation:** Jiling Zhang.

**Methodology:** Jiling Zhang, Baodong Wang, Peng Du.

**Resources:** Jiling Zhang, Baodong Wang, Peng Du, He Song, Yu Zhou.

**Software:** Jiling Zhang, Baodong Wang, Peng Du, He Song.

**Supervision:** Jiling Zhang, Baodong Wang, Peng Du, He Song, Lihui Yang.

**Validation:** Jiling Zhang, Baodong Wang, Peng Du, He Song, Lihui Yang.

**Visualization:** Jiling Zhang, Baodong Wang, Peng Du, He Song, Lihui Yang.

**Writing – original draft:** Jiling Zhang, Baodong Wang, Peng Du.

**Writing – review & editing:** Yu Zhou.

## Supplementary Material


