# Supplementary material for: Gut-disc axis: A Mendelian randomization study on the relationship between gut microbiota and cervical spondylosis
Source: Medicine (Baltimore). 2025 Feb 14;104(7):e41536. doi: 10.1097/MD.0000000000041536 (PMC11835084; doi:10.1097/MD.0000000000041536)
Supplement: Supplementary file 1 [file medi-104-e41536-s001.pdf]

Supplementary Figure S1. Scatter plots for the causal association between gut microbiota and cervical spondylosis.

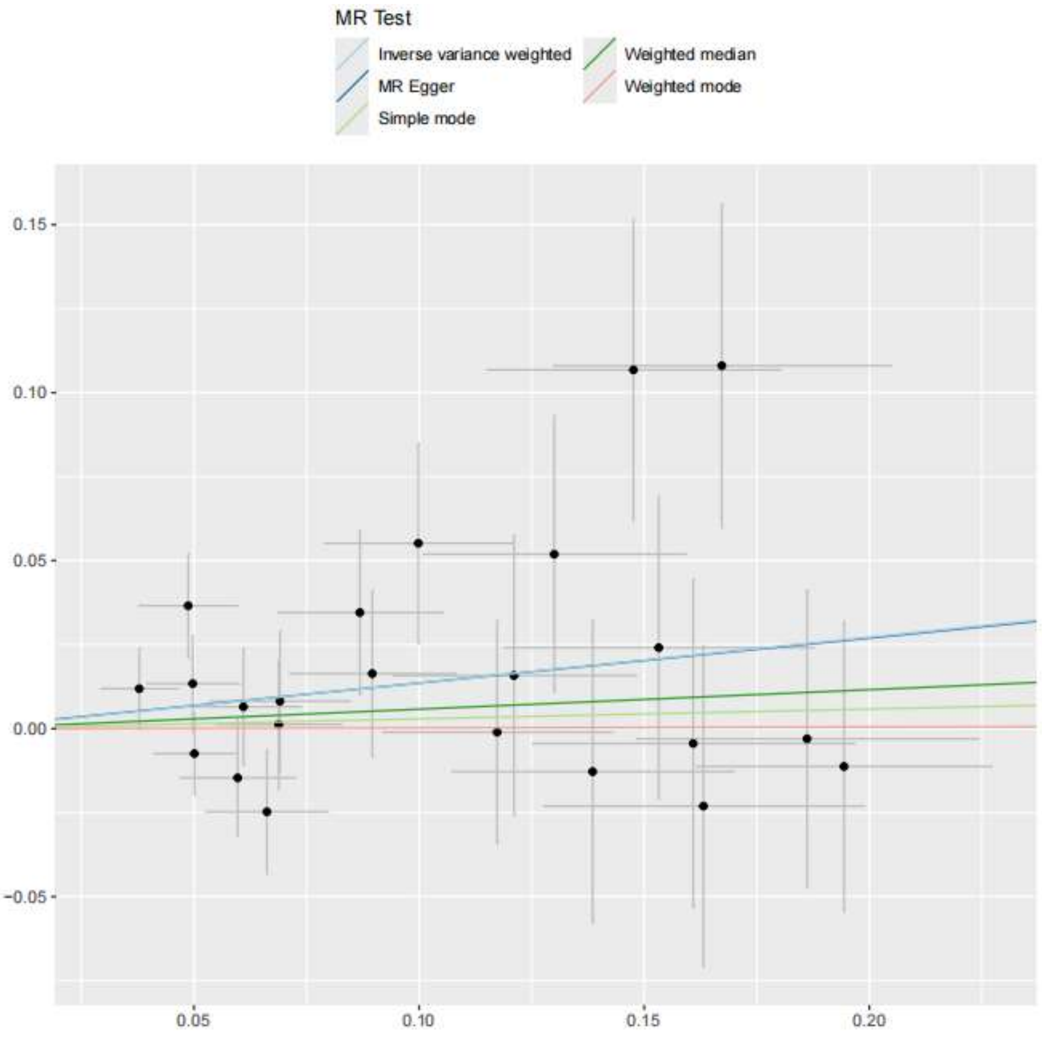

SNP effect on cervical spondylosis

SNP effect on Anaeromassilibacillus sp001305115 abundance in stool

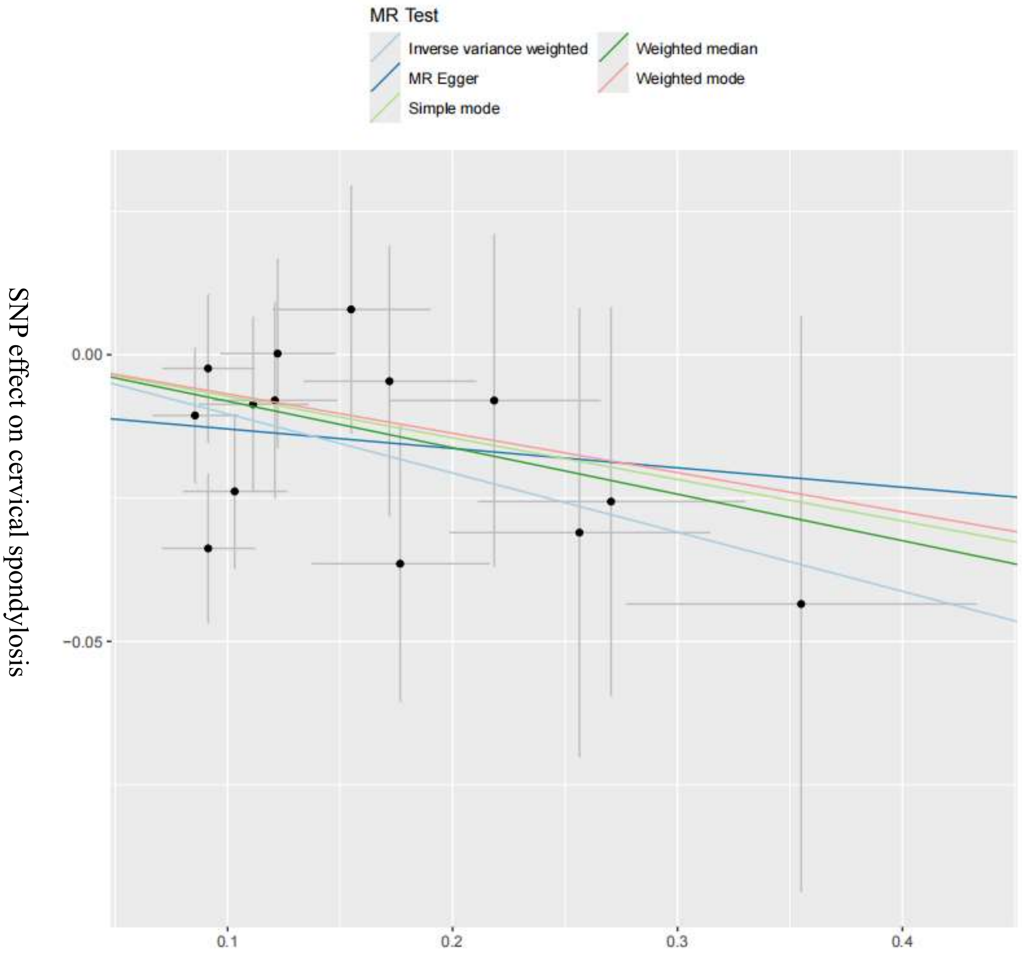

SNP effect on Bacteroides A plebeius A abundance in stool

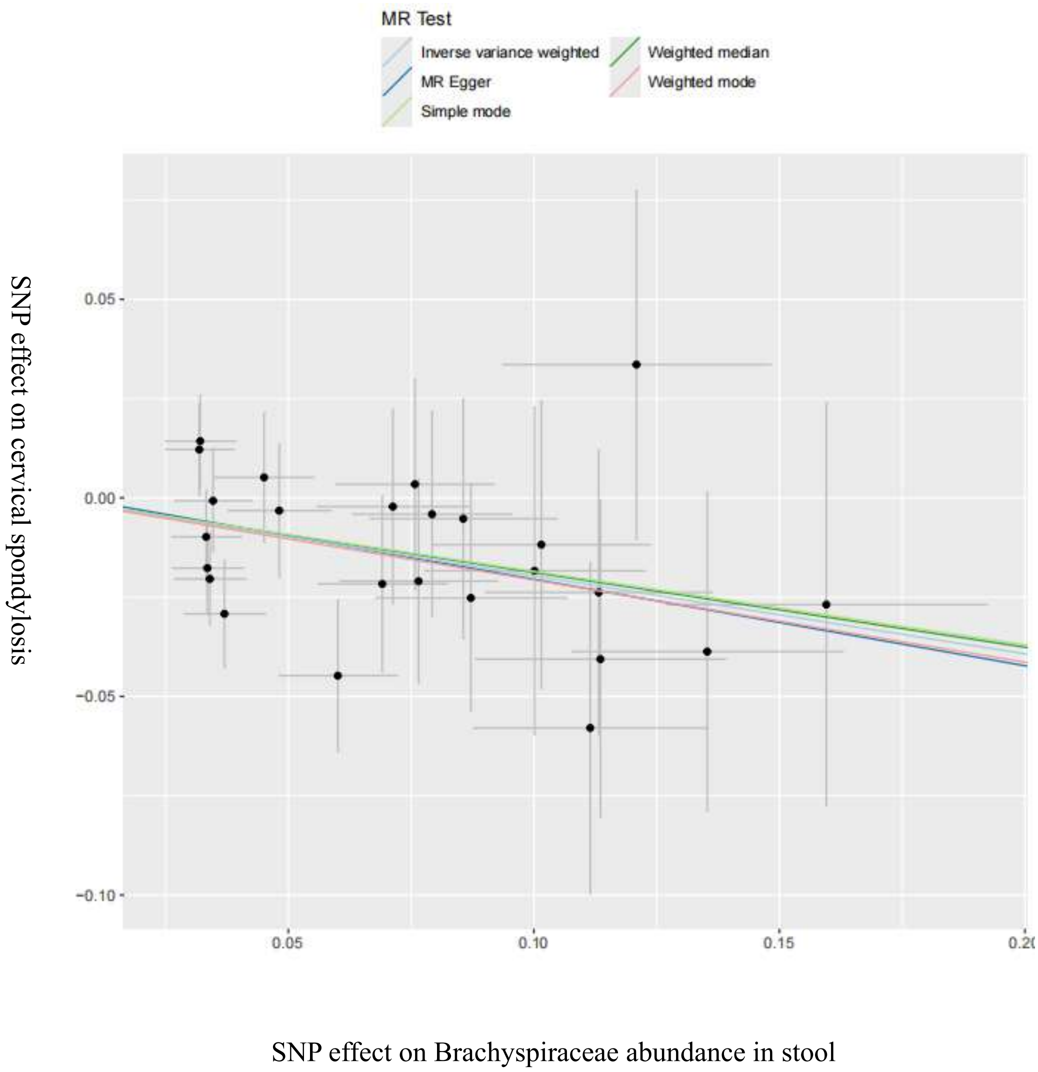
MR Test
Inverse variance weighted
MR Egger
Simple mode
Weighted median
Weighted mode
SNP effect on cervical spondylosis
0.05
0.00
-0.05
-0.10
0.05
0.10
0.15
SNP effect on Brachyspiraceae abundance in stool

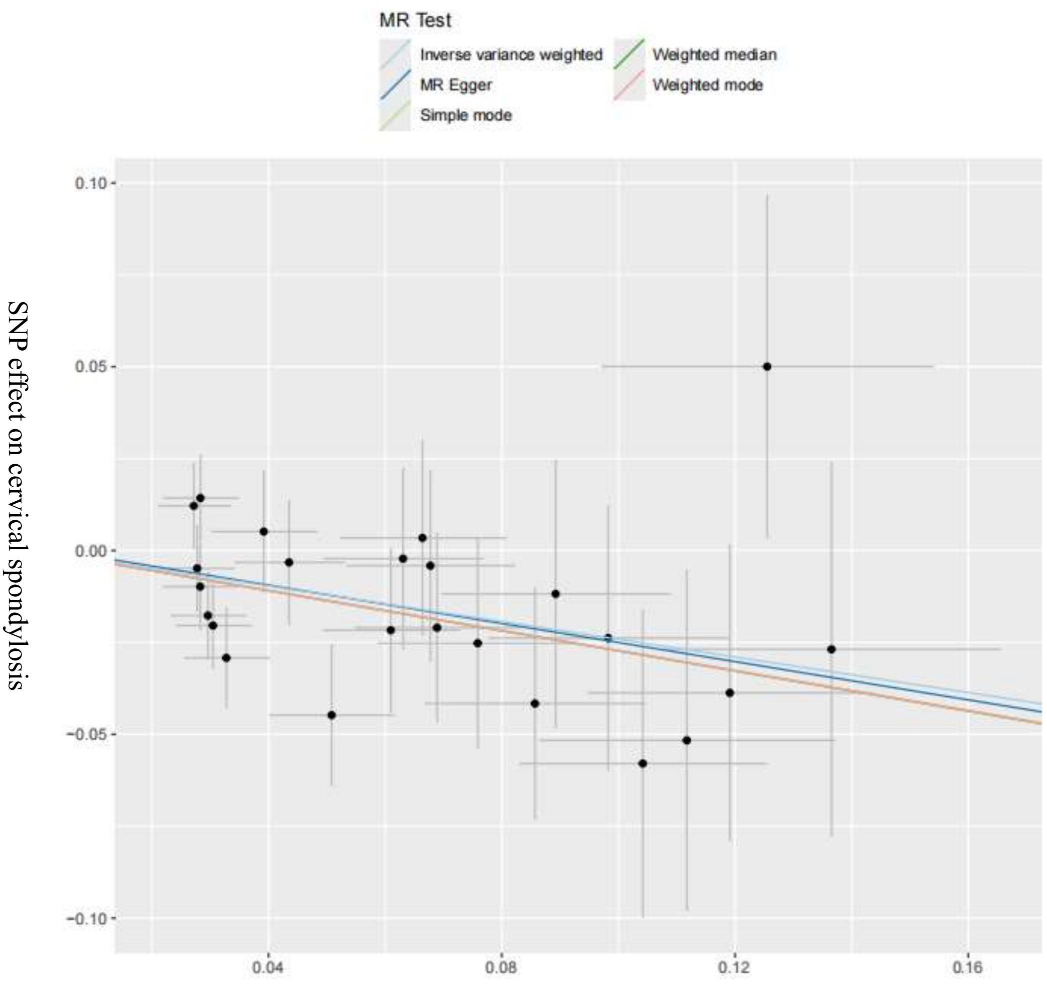

SNP effect on Brachyspirae abundance in stool

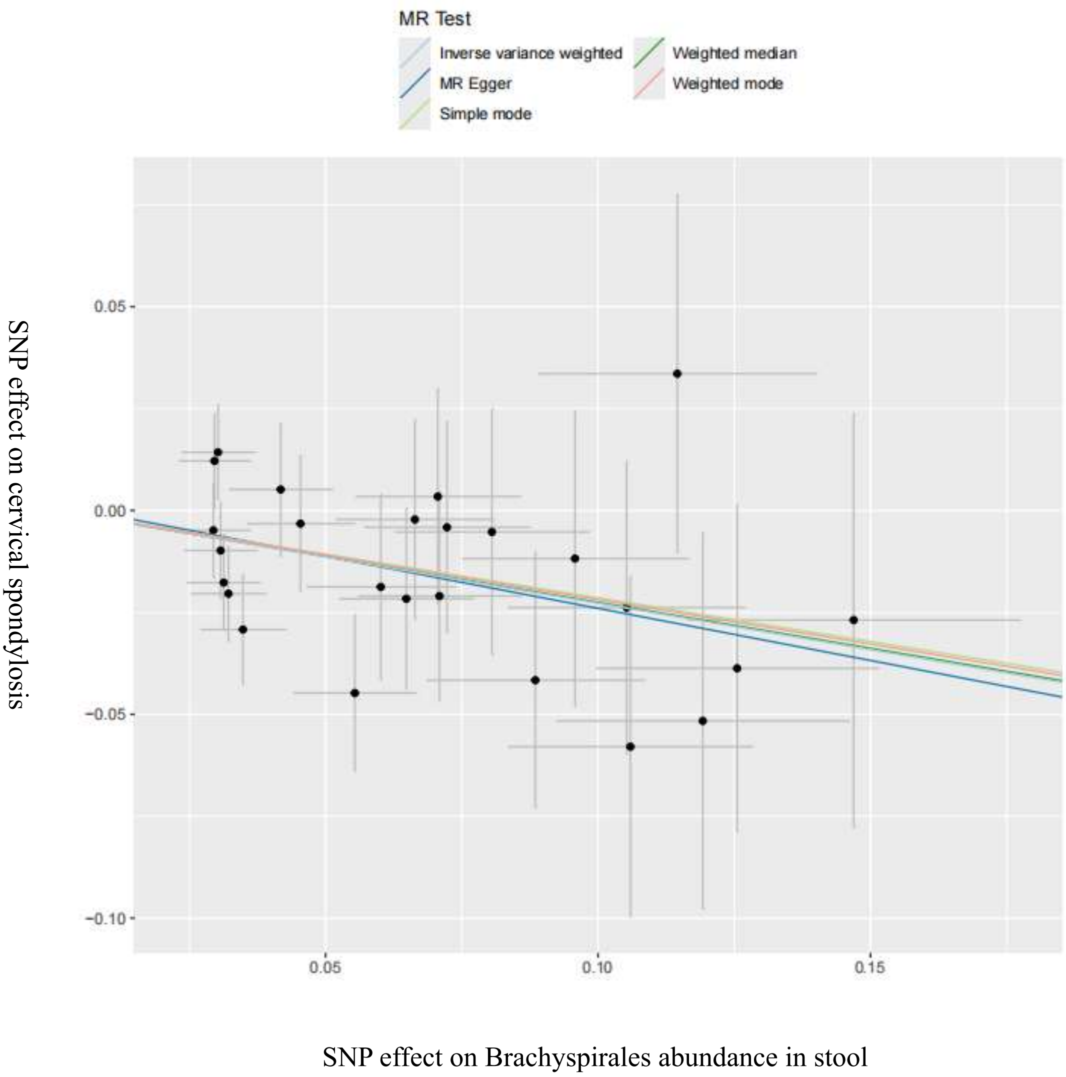
MR Test
Inverse variance weighted
MR Egger
Simple mode
Weighted median
Weighted mode
0.05
0.00
-0.05
-0.10
0.05
0.10
0.15
SNP effect on cervical spondylosis
SNP effect on Brachyspirales abundance in stool

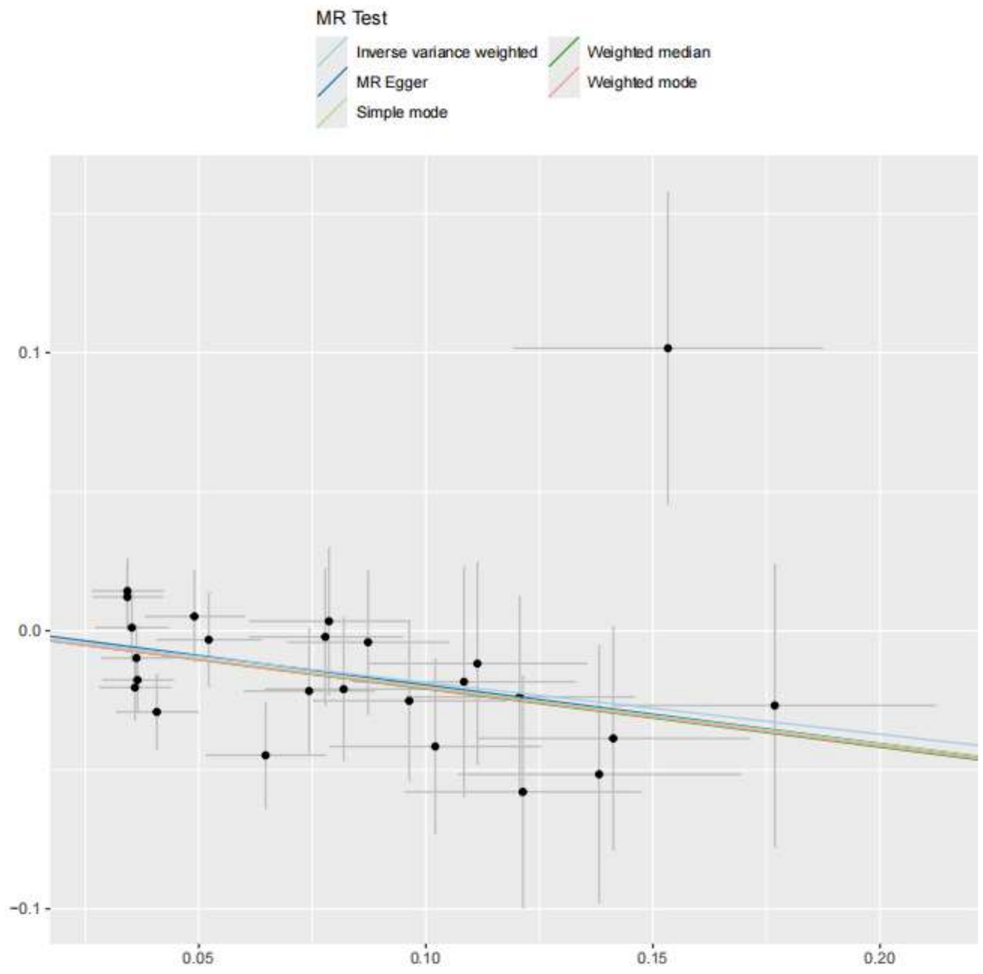

SNP effect on cervical spondylosis

SNP effect on Brachyspira abundance in stool

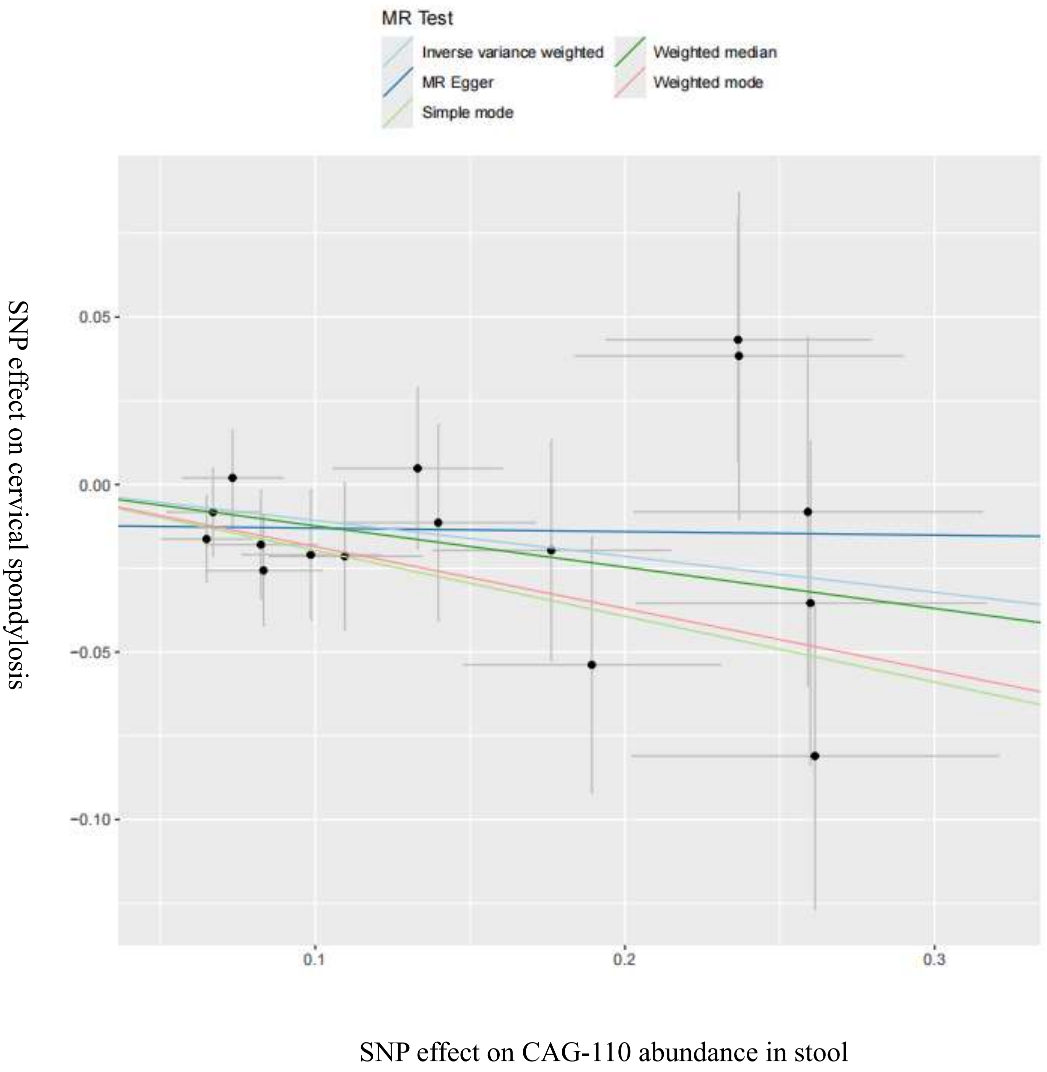
MR Test
Inverse variance weighted
MR Egger
Simple mode
Weighted median
Weighted mode
0.05
0.00
−0.05
−0.10
0.1
0.2
0.3
SNP effect on cervical spondylosis
SNP effect on CAG-110 abundance in stool

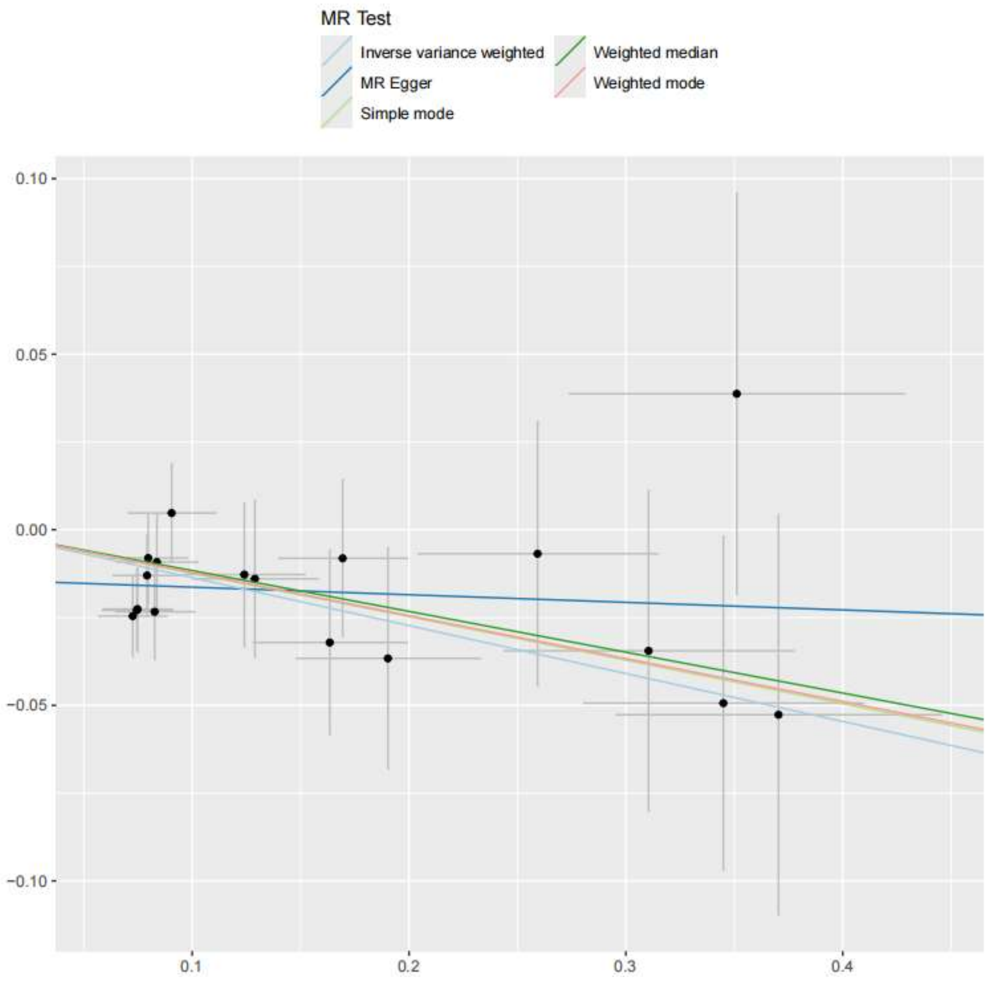

SNP effect on cervical spondylosis

SNP effect on CAG-448 sp003150135 abundance in stool

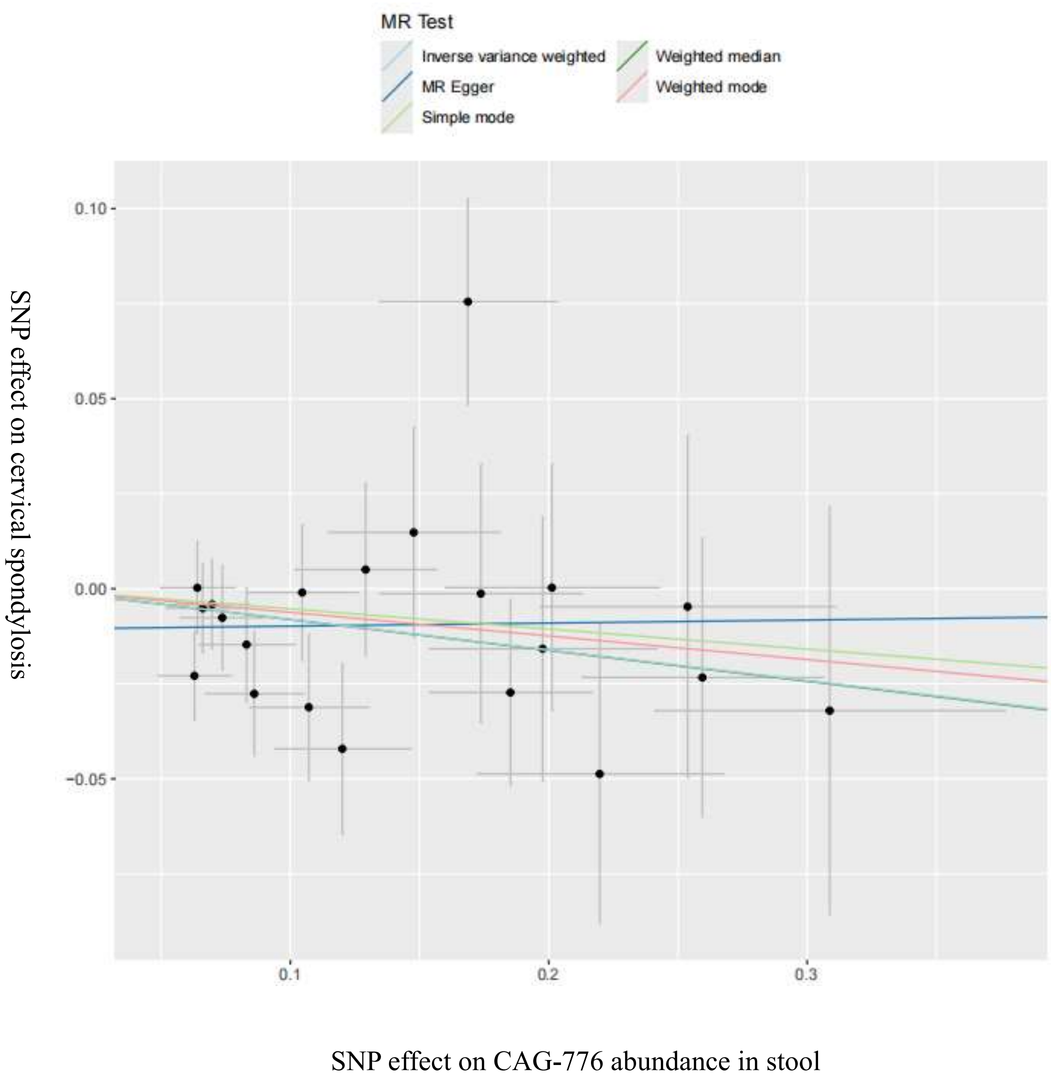
MR Test
Inverse variance weighted
MR Egger
Simple mode
Weighted median
Weighted mode
0.10
0.05
0.00
−0.05
0.1
0.2
0.3
SNP effect on cervical spondylosis
SNP effect on CAG-776 abundance in stool

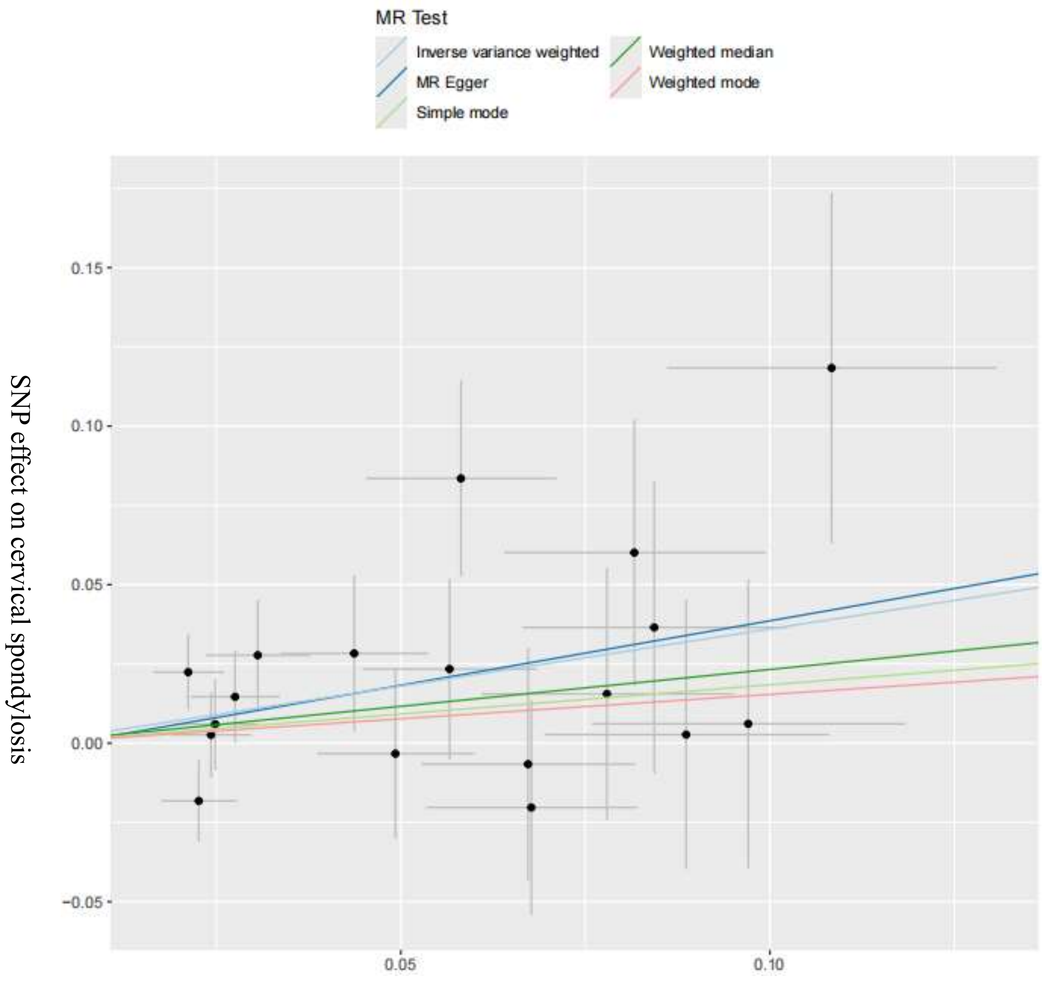

SNP effect on Comamonas B abundance in stool

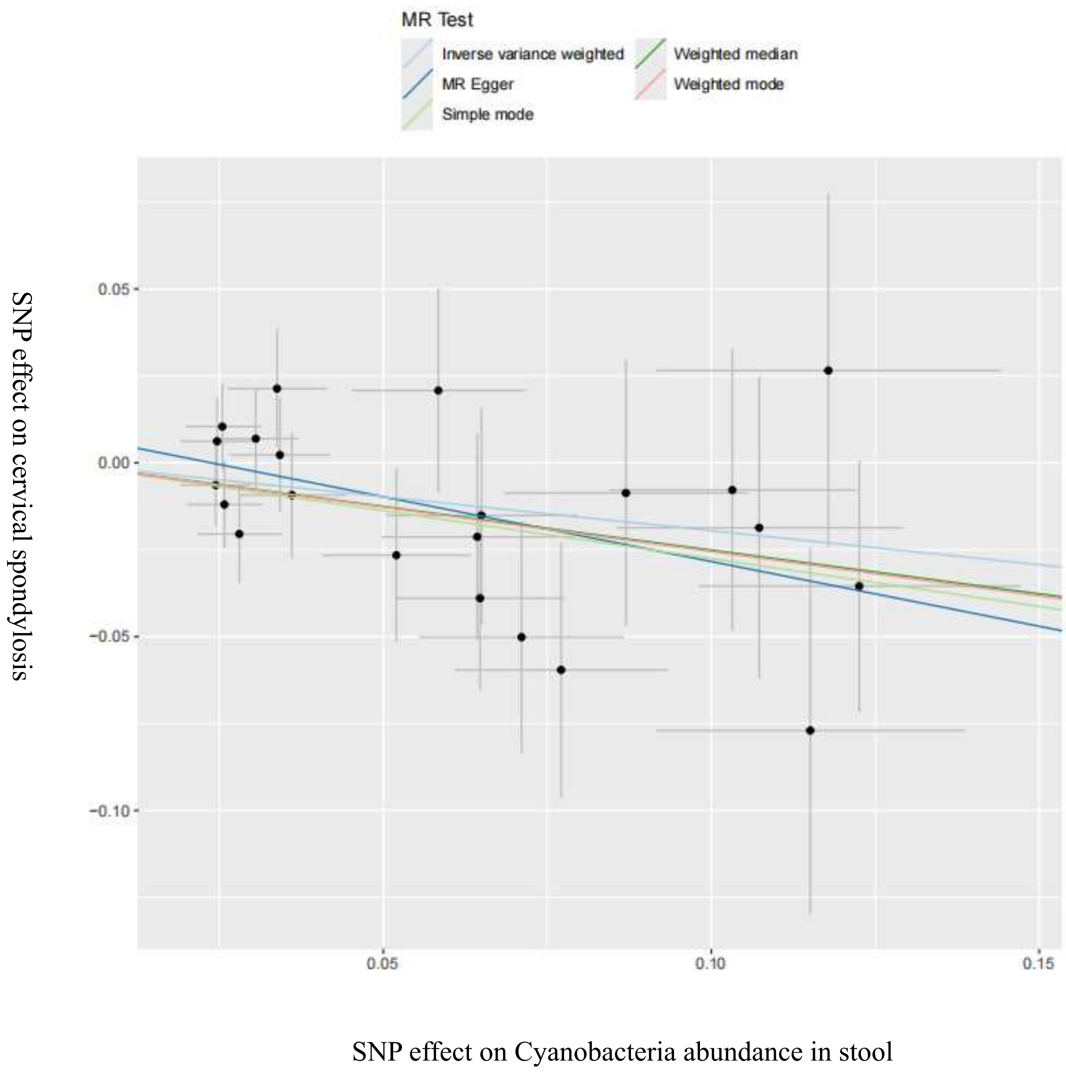
MR Test
Inverse variance weighted
MR Egger
Simple mode
Weighted median
Weighted mode
SNP effect on cervical spondylosis
0.05
0.00
−0.05
−0.10
0.05
0.10
0.15
SNP effect on Cyanobacteria abundance in stool

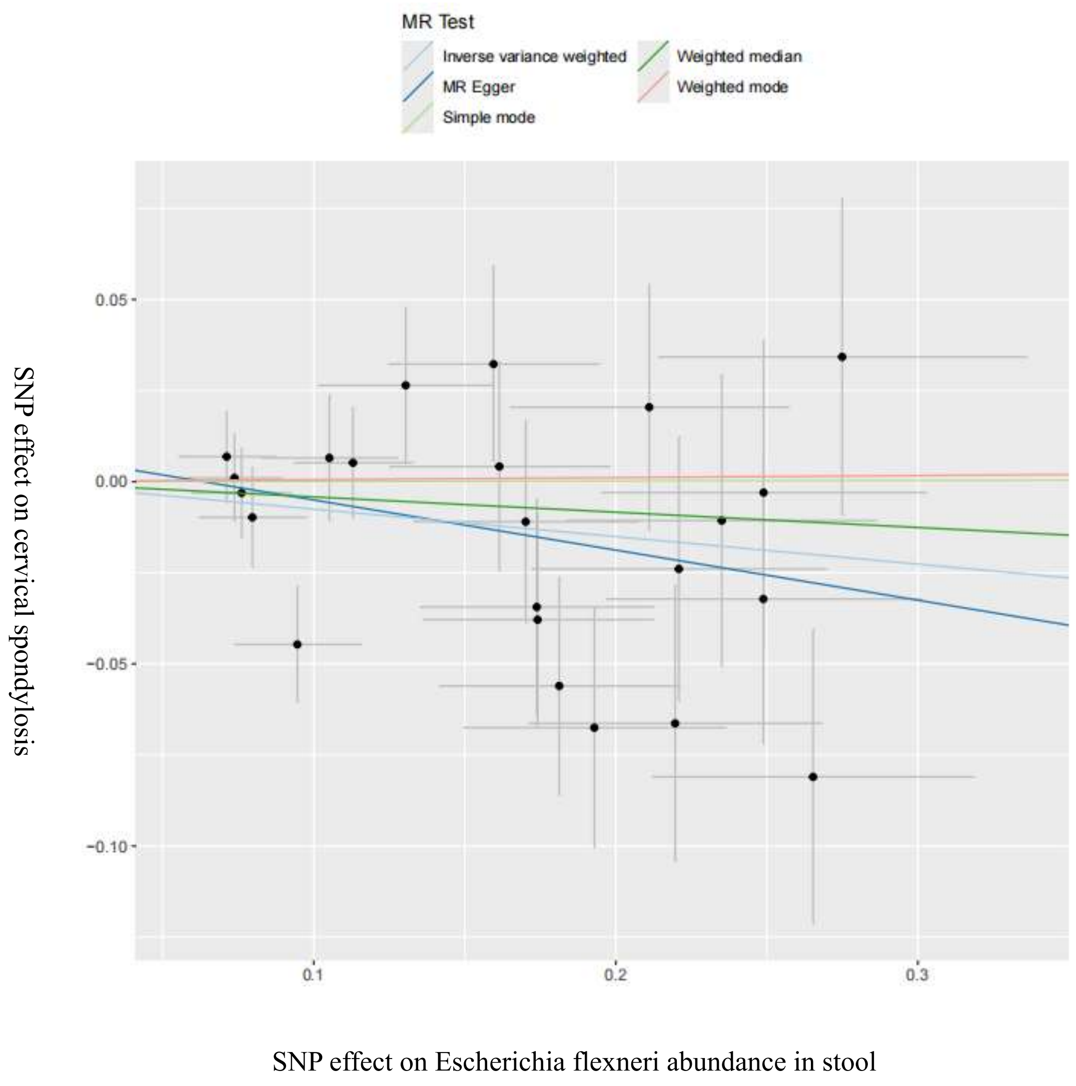
MR Test
Inverse variance weighted
MR Egger
Simple mode
Weighted median
Weighted mode
0.05
0.00
−0.05
−0.10
0.1
0.2
0.3
SNP effect on cervical spondylosis
SNP effect on Escherichia flexneri abundance in stool

SNP effect on cervical spondylosis

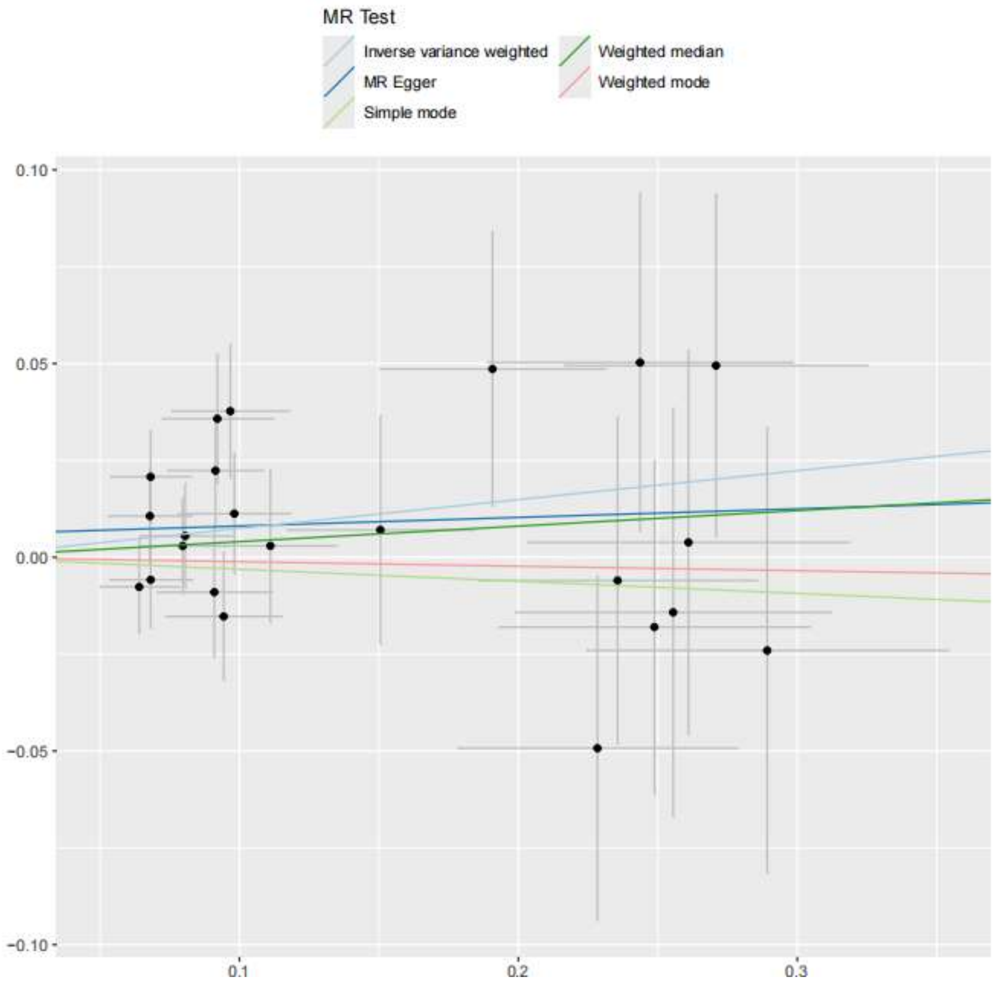

SNP effect on Faecalicatena torques abundance in stool

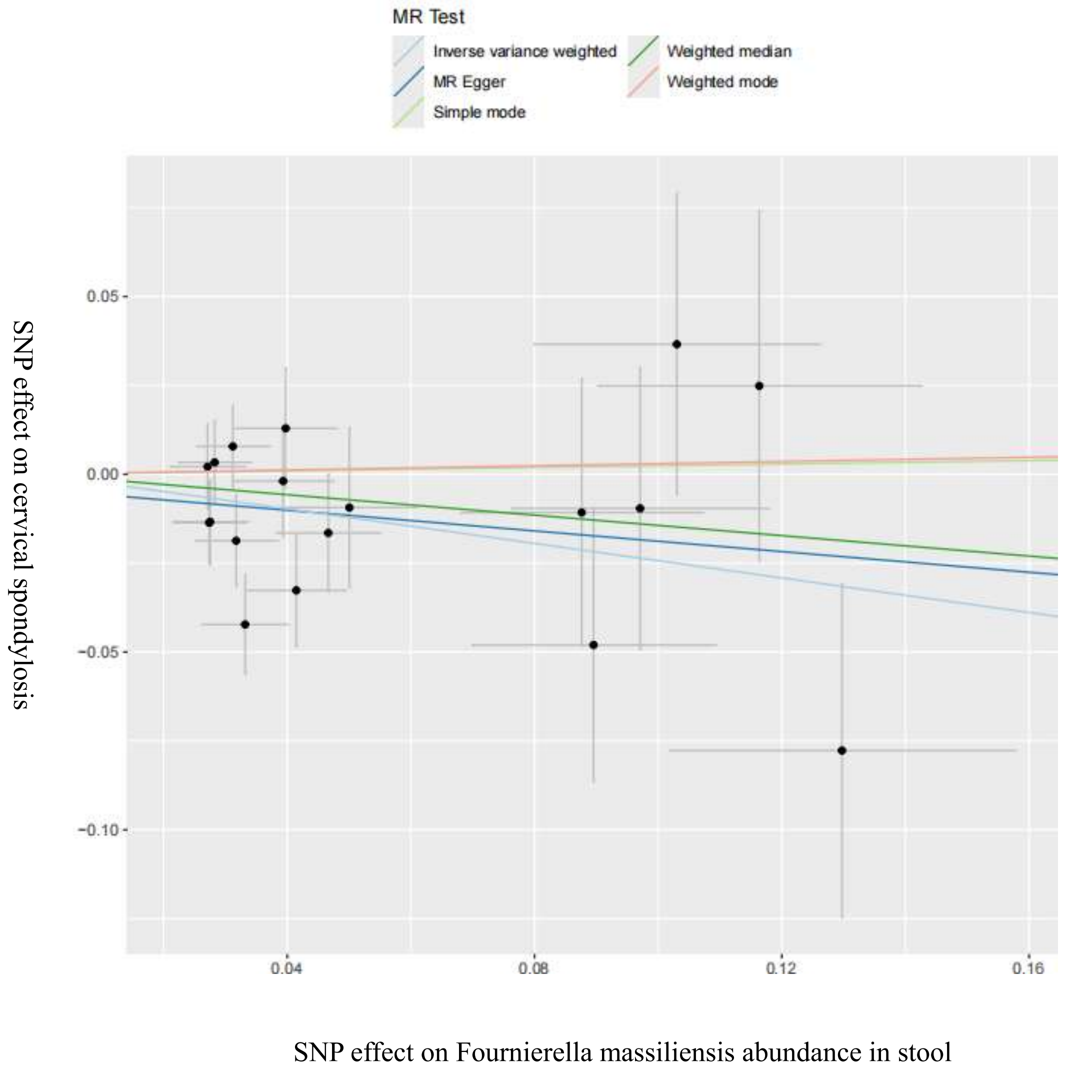
MR Test
Inverse variance weighted
MR Egger
Simple mode
Weighted median
Weighted mode
0.05
0.00
-0.05
-0.10
0.04
0.08
0.12
0.16
SNP effect on cervical spondylosis
SNP effect on Fournierella massiliensis abundance in stool

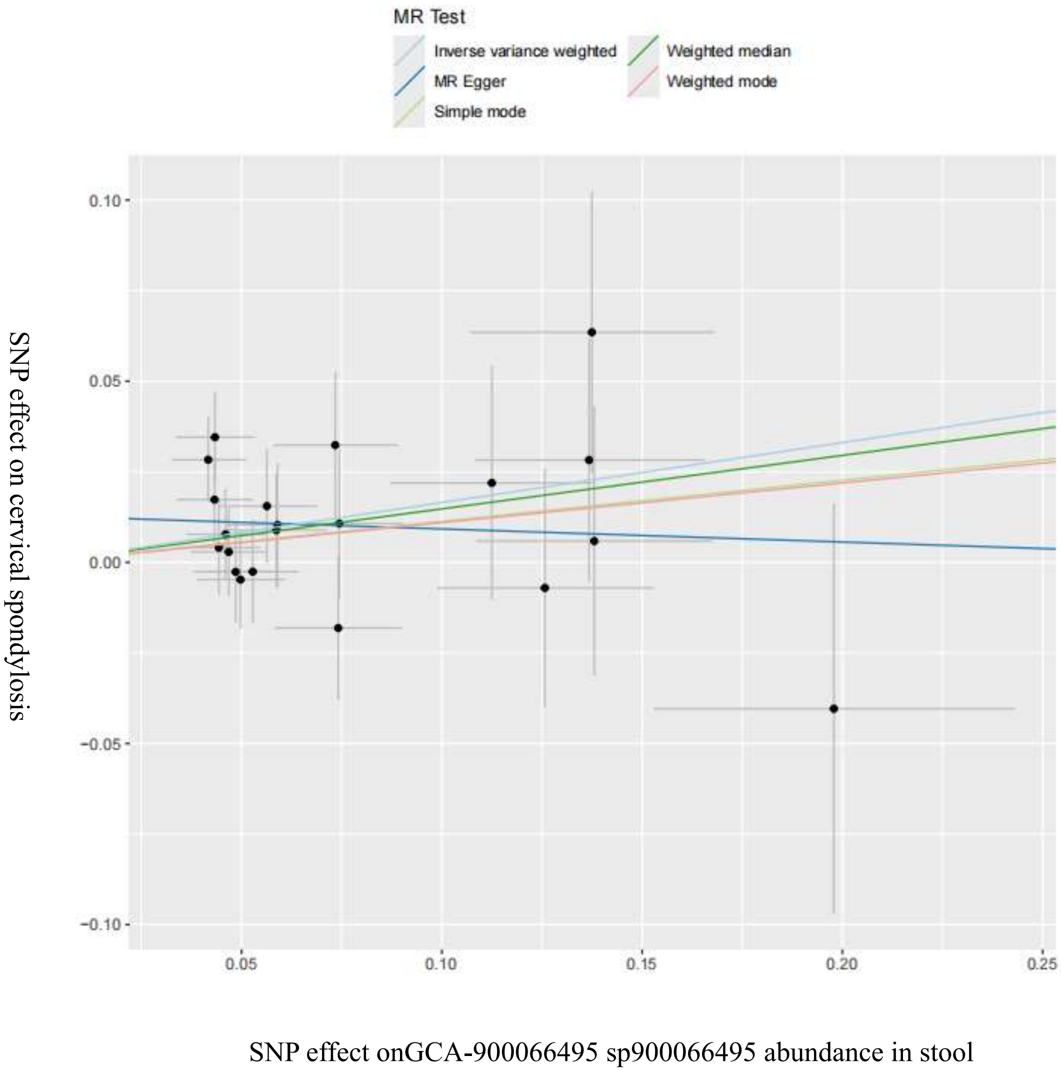
MR Test
Inverse variance weighted
MR Egger
Simple mode
Weighted median
Weighted mode
0.10
0.05
0.00
−0.05
−0.10
0.05
0.10
0.15
0.20
0.25
SNP effect on cervical spondylosis
SNP effect onGCA-900066495 sp900066495 abundance in stool

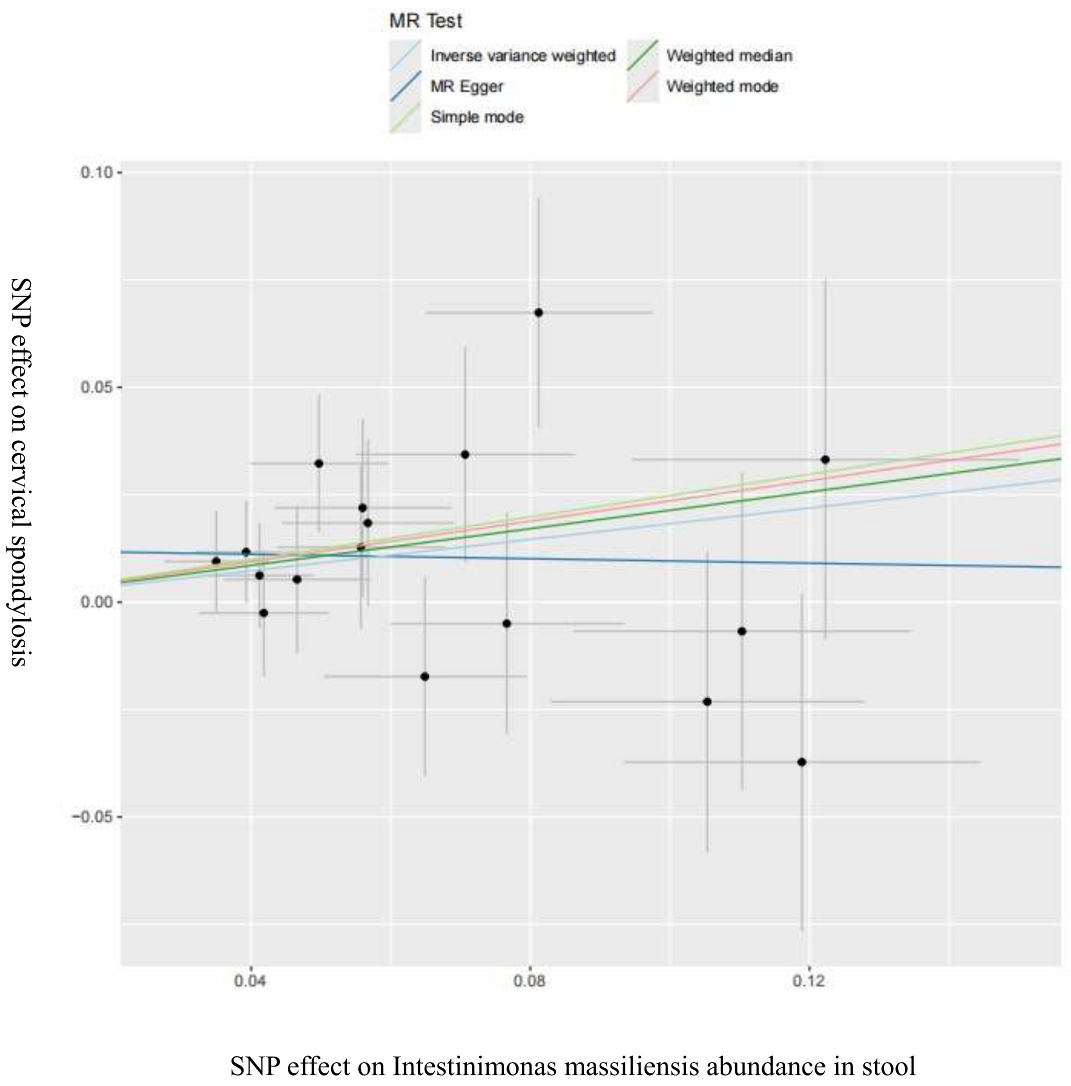
MR Test
Inverse variance weighted
MR Egger
Simple mode
Weighted median
Weighted mode
0.10
0.05
0.00
−0.05
0.04
0.08
0.12
SNP effect on cervical spondylosis
SNP effect on Intestinimonas massiliensis abundance in stool

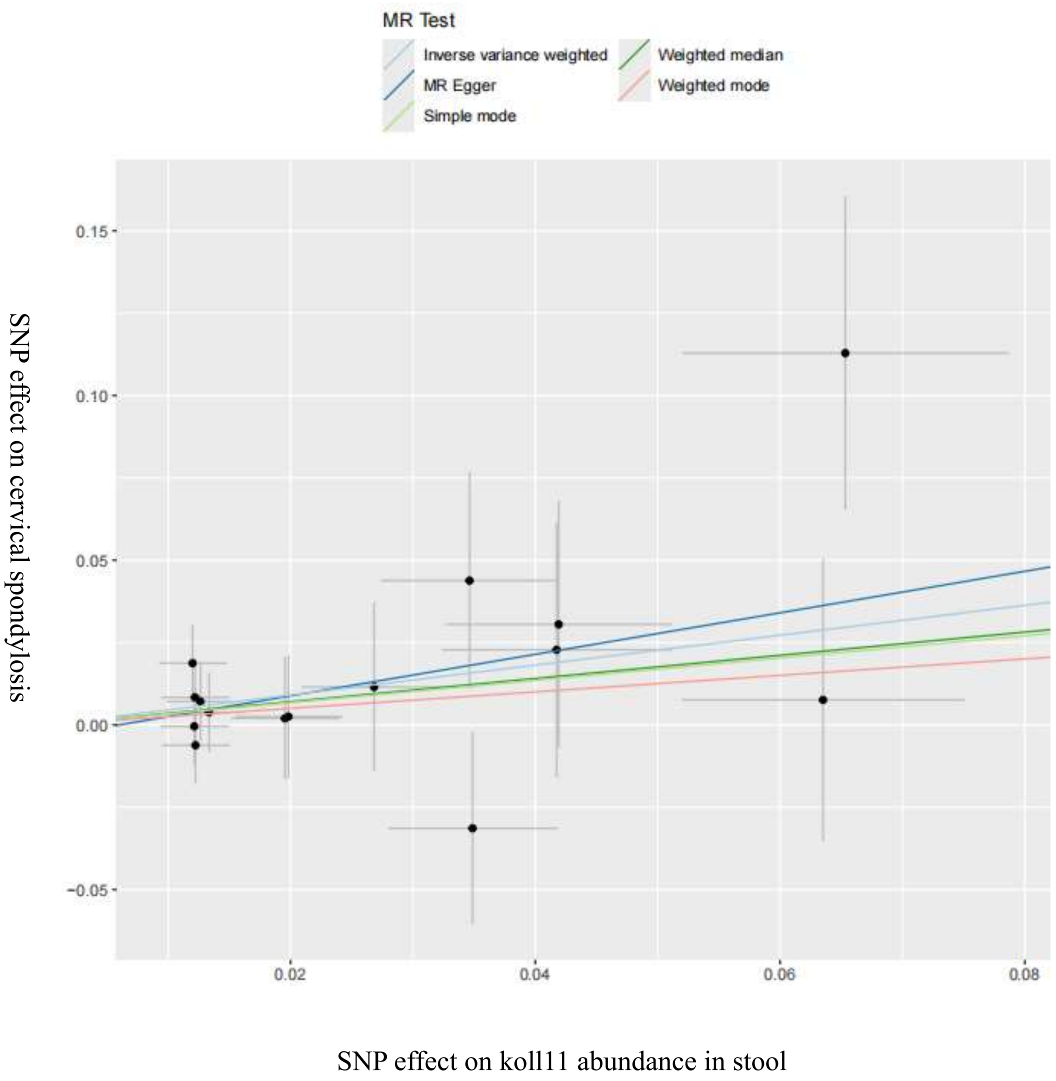
MR Test
Inverse variance weighted
MR Egger
Simple mode
Weighted median
Weighted mode
0.15
0.10
0.05
0.00
-0.05
0.02
0.04
0.06
0.08
SNP effect on cervical spondylosis
SNP effect on koll11 abundance in stool

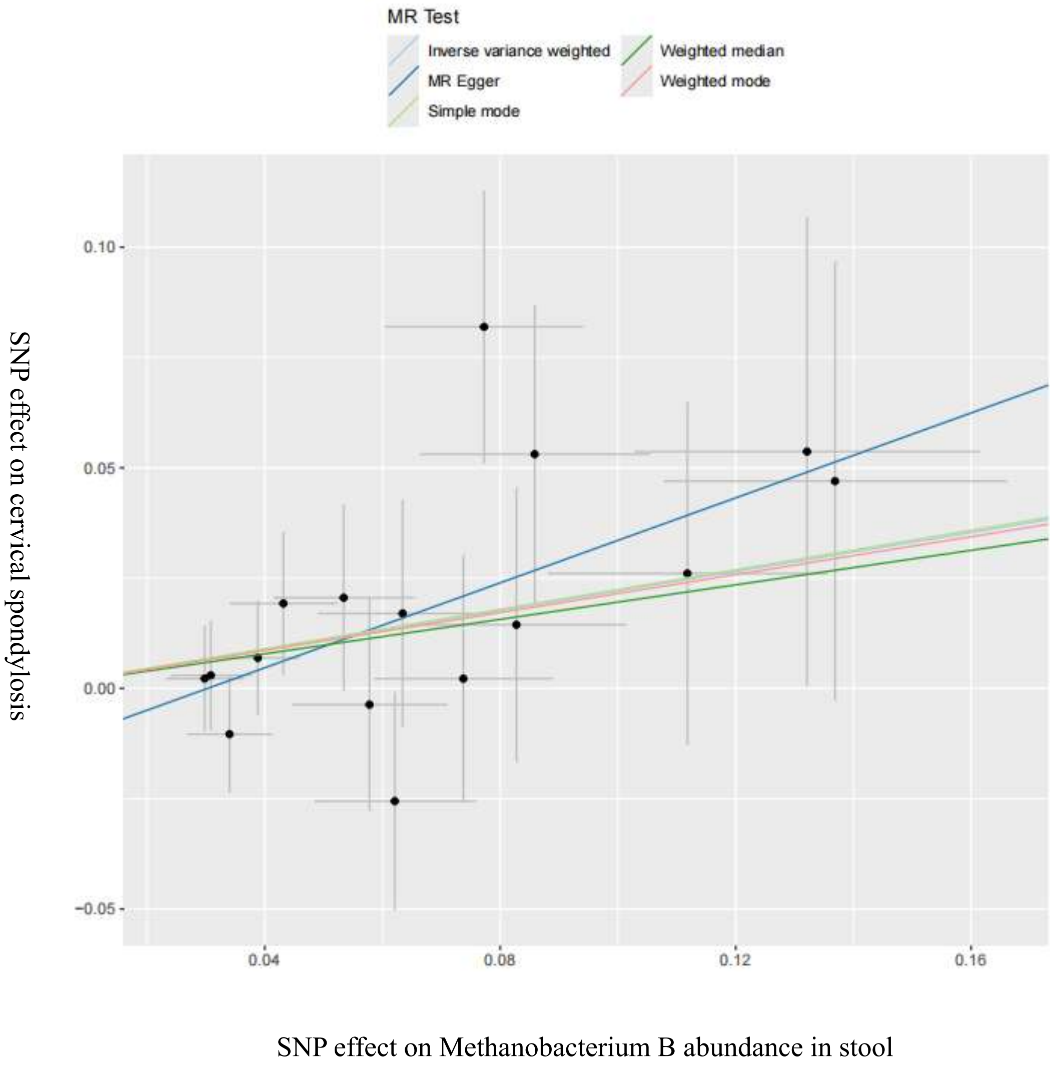

MR Test
Inverse variance weighted
MR Egger
Simple mode
Weighted median
Weighted mode
0.10
0.05
0.00
−0.05
0.04
0.08
0.12
0.16
SNP effect on cervical spondylosis
SNP effect on Methanobacterium B abundance in stool

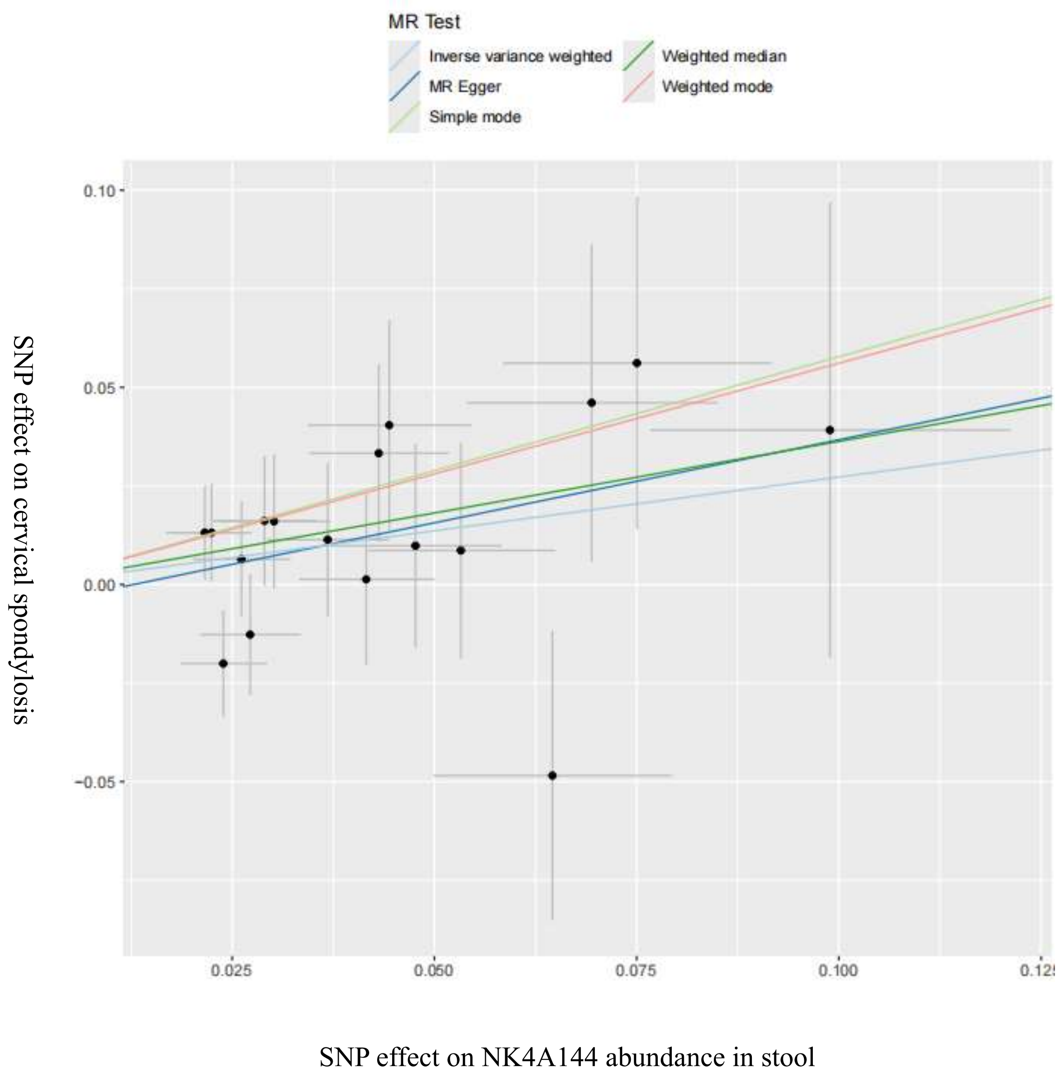
MR Test
Inverse variance weighted
MR Egger
Simple mode
Weighted median
Weighted mode
0.10
0.05
0.00
−0.05
0.025
0.050
0.075
0.100
SNP effect on cervical spondylosis
SNP effect on NK4A144 abundance in stool

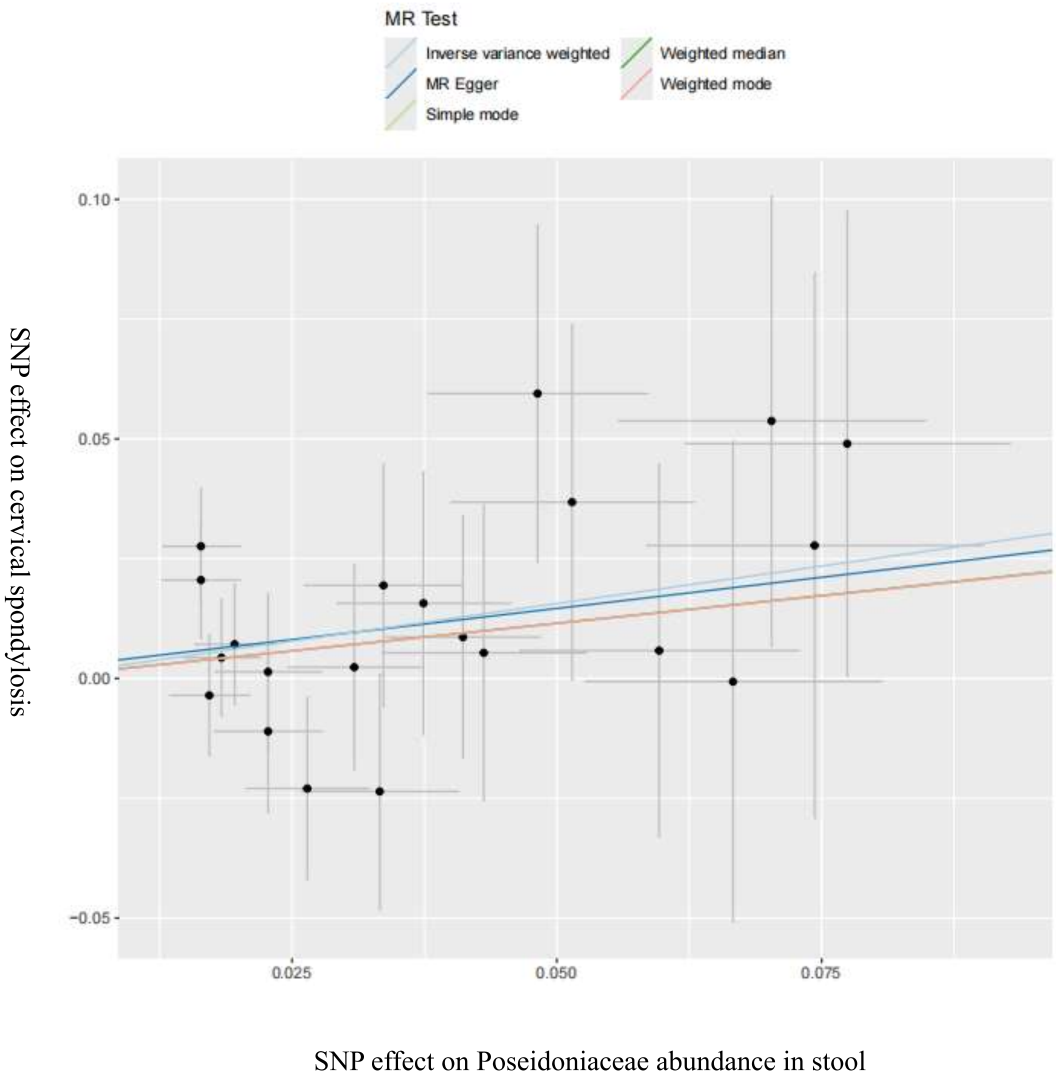
MR Test
Inverse variance weighted
MR Egger
Simple mode
Weighted median
Weighted mode
0.10
0.05
0.00
−0.05
0.025
0.050
0.075
SNP effect on cervical spondylosis
SNP effect on Poseidoniaceae abundance in stool

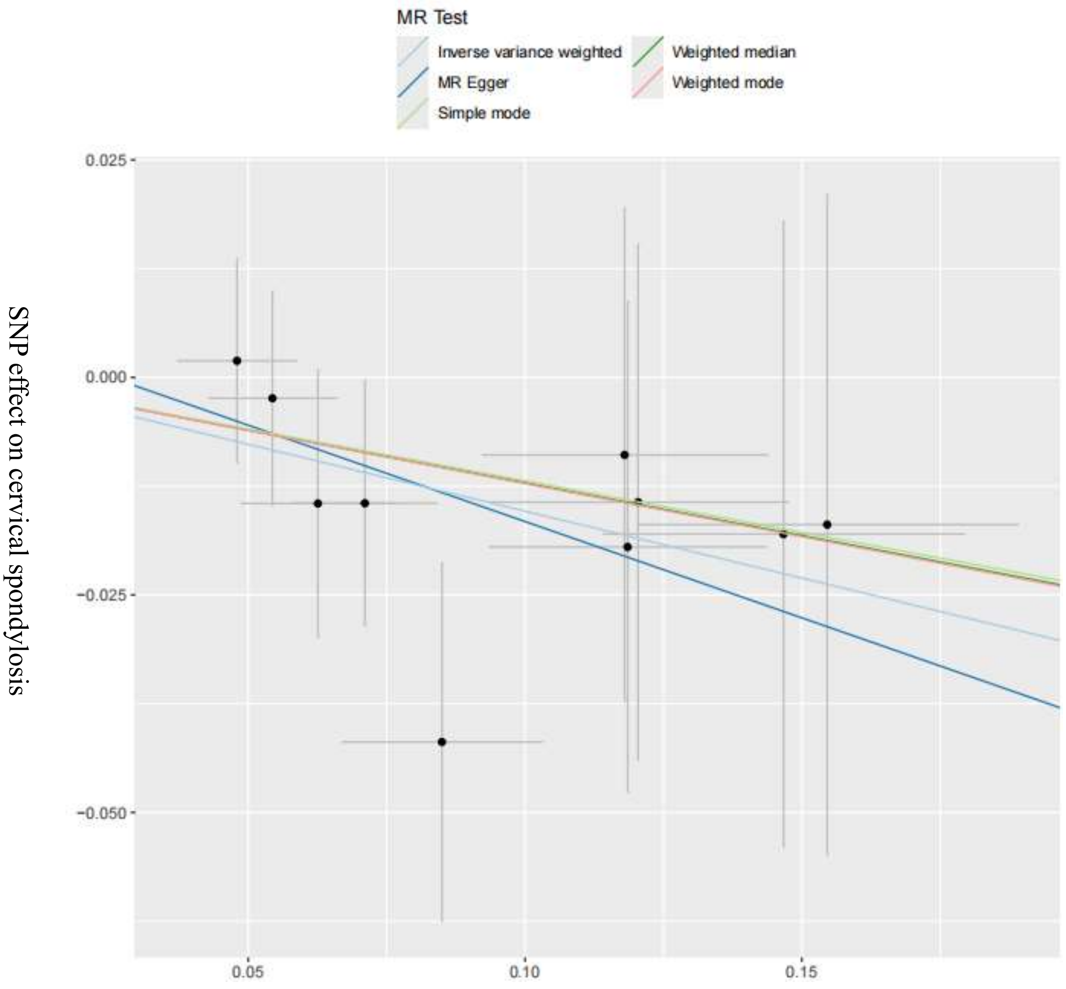

SNP effect on Prevotella sp900318625 abundance in stool

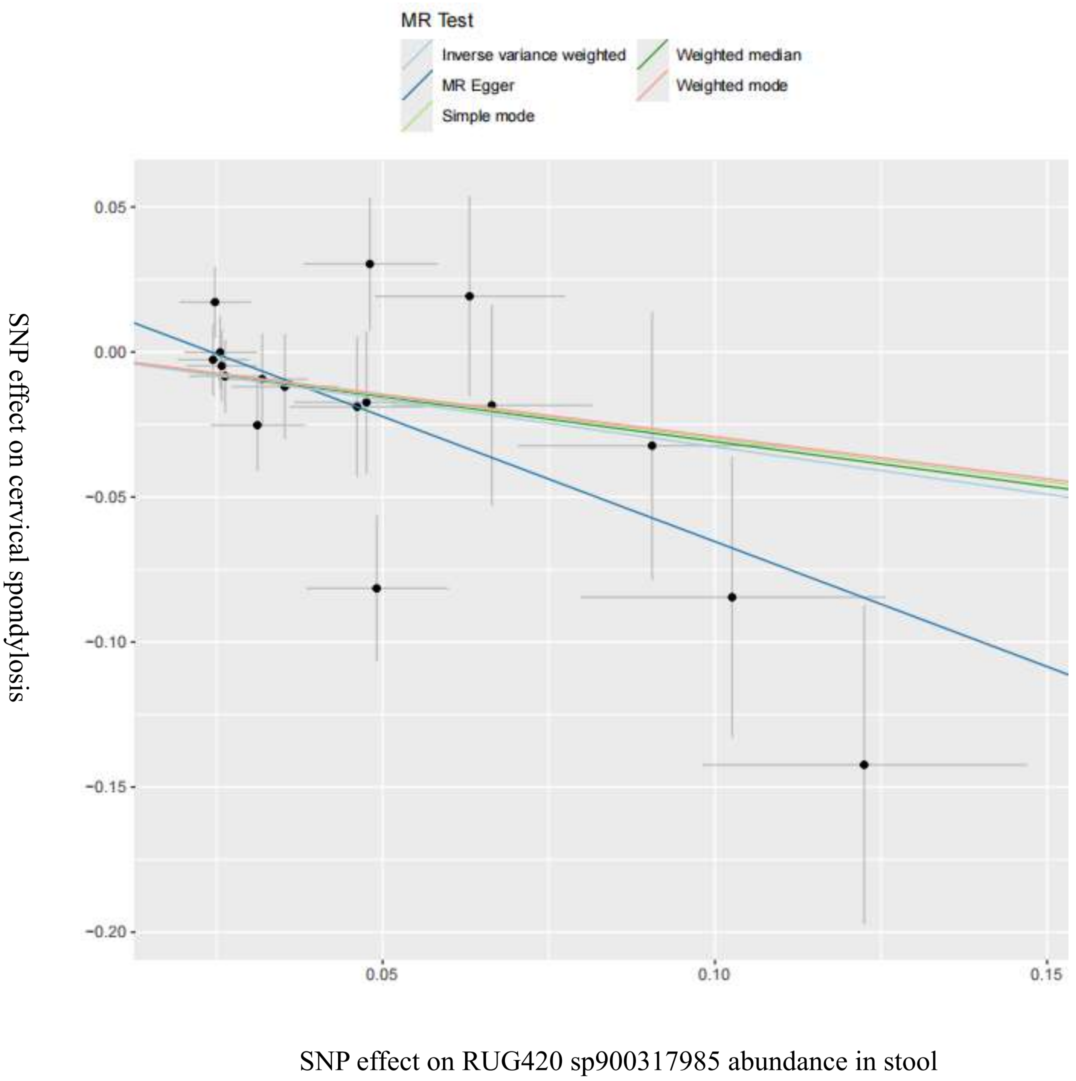
MR Test
Inverse variance weighted
MR Egger
Simple mode
Weighted median
Weighted mode
0.05
0.00
−0.05
−0.10
−0.15
−0.20
0.05
0.10
0.15
SNP effect on cervical spondylosis
SNP effect on RUG420 sp900317985 abundance in stool

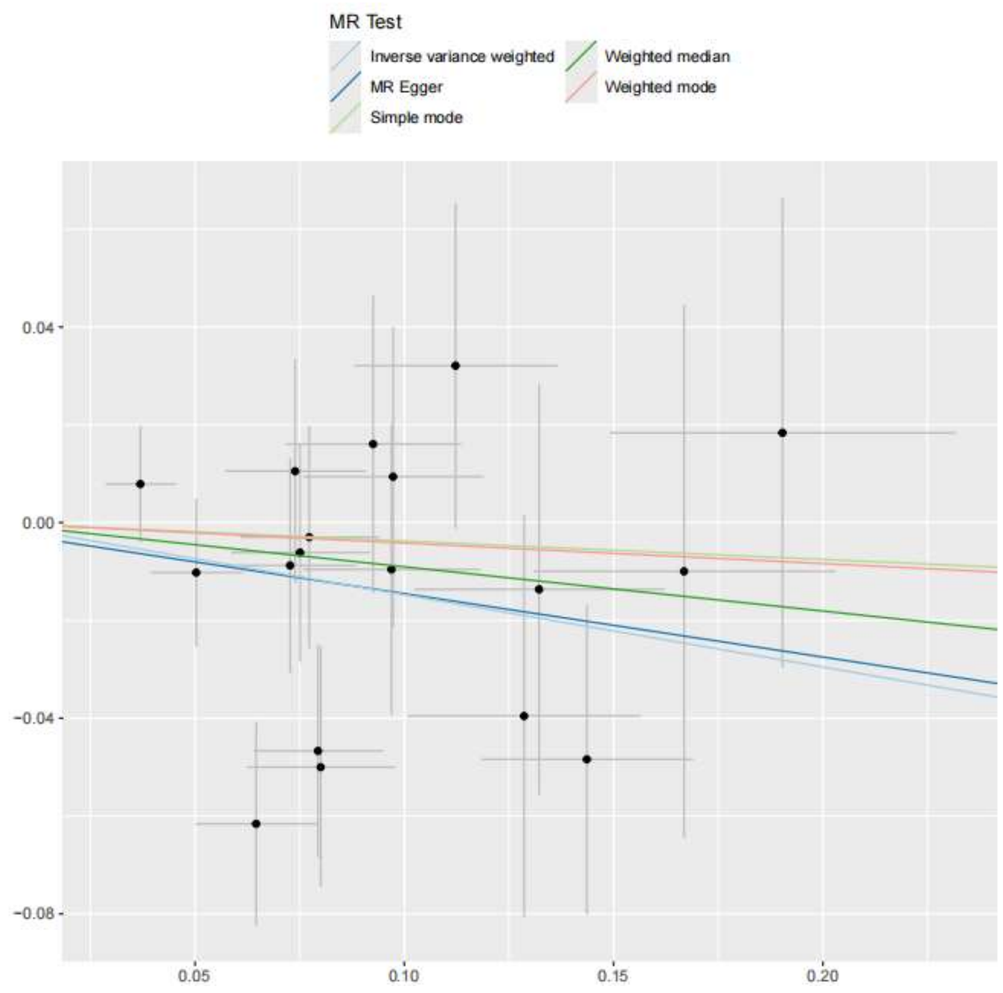

SNP effect on cervical spondylosis

SNP effect on Ruminococcus A sp000432335 abundance in stool

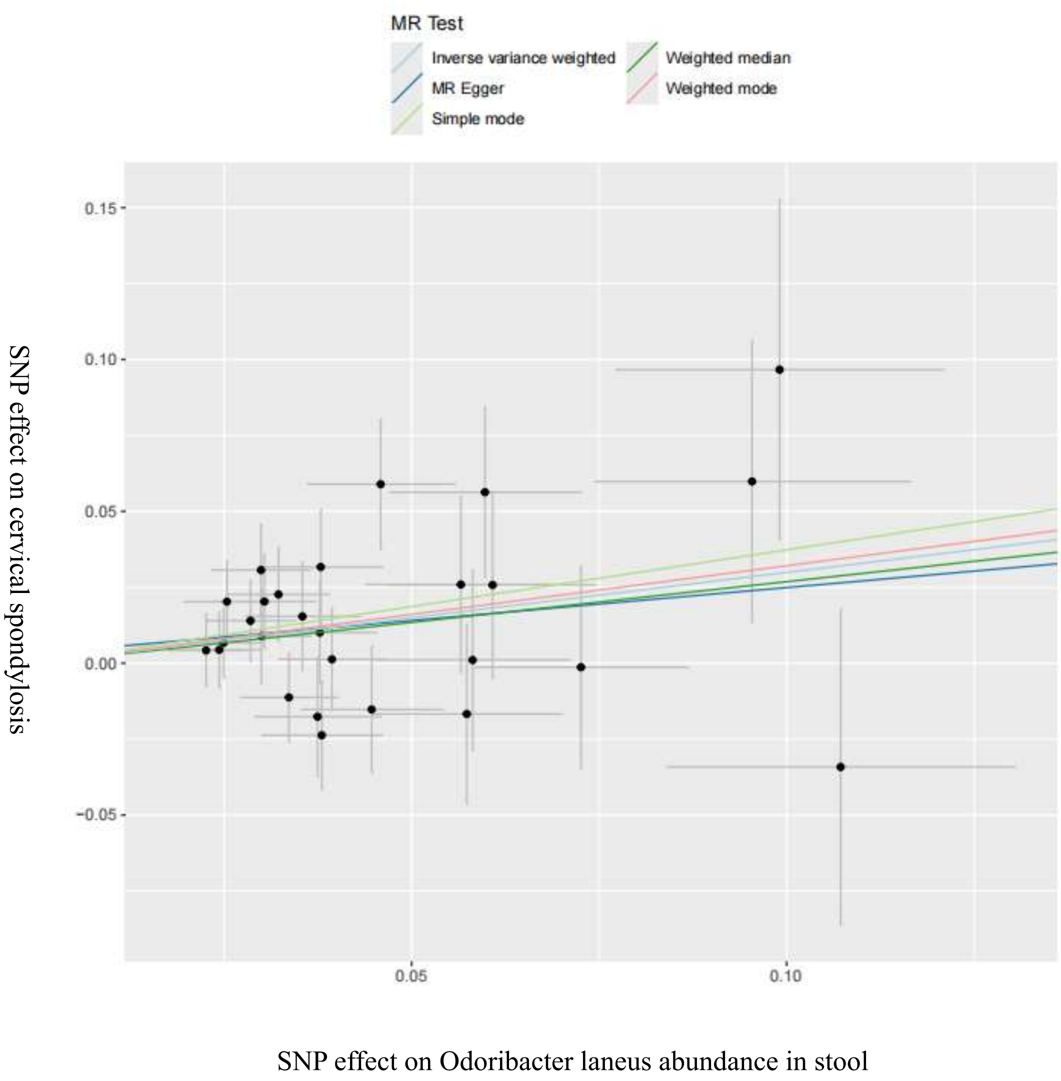
MR Test
Inverse variance weighted
MR Egger
Simple mode
Weighted median
Weighted mode
0.15
0.10
0.05
0.00
−0.05
0.05
0.10
SNP effect on cervical spondylosis
SNP effect on Odoribacter laneus abundance in stool

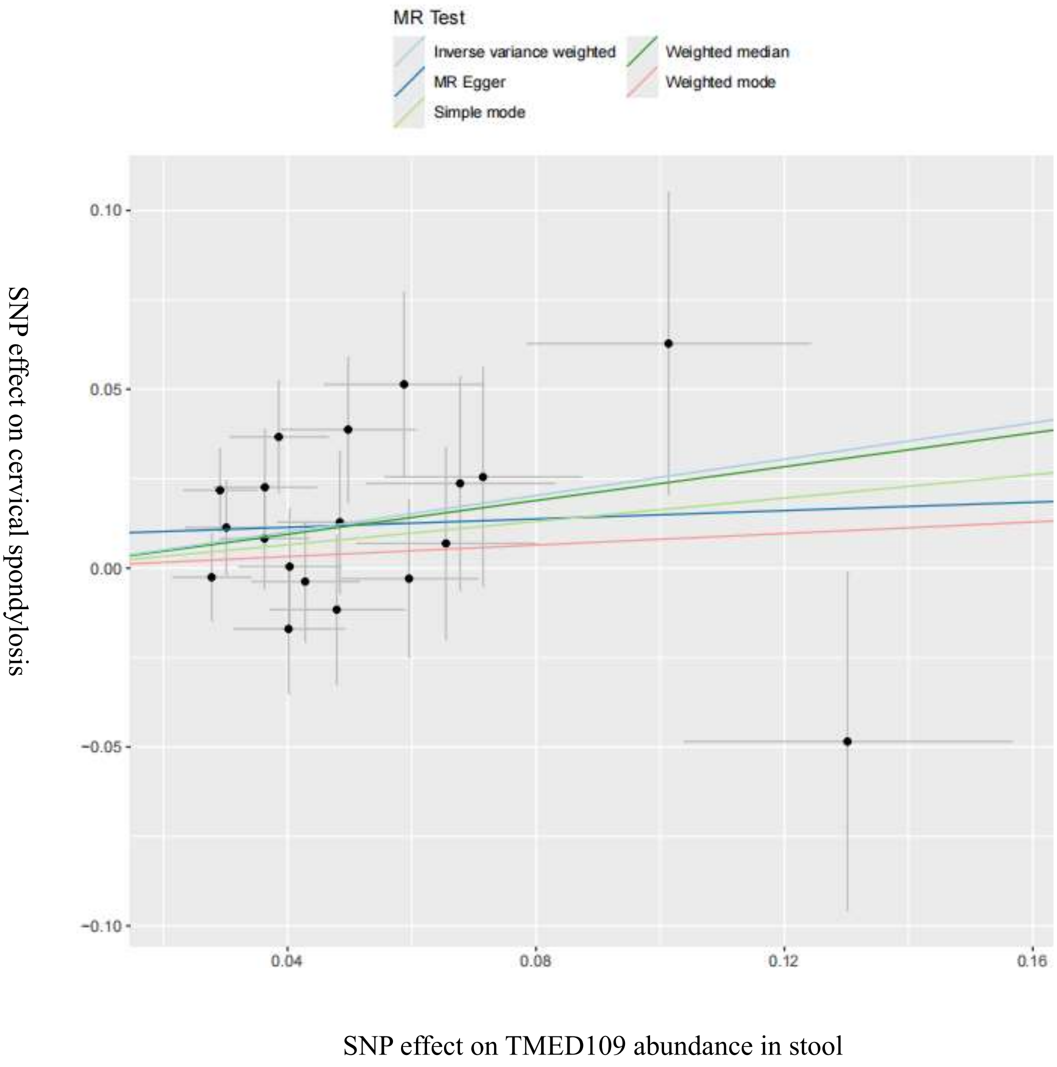

MR Test
Inverse variance weighted
MR Egger
Simple mode
Weighted median
Weighted mode
0.10
0.05
0.00
−0.05
−0.10
0.04
0.08
0.12
0.16
SNP effect on cervical spondylosis
SNP effect on TMED109 abundance in stool

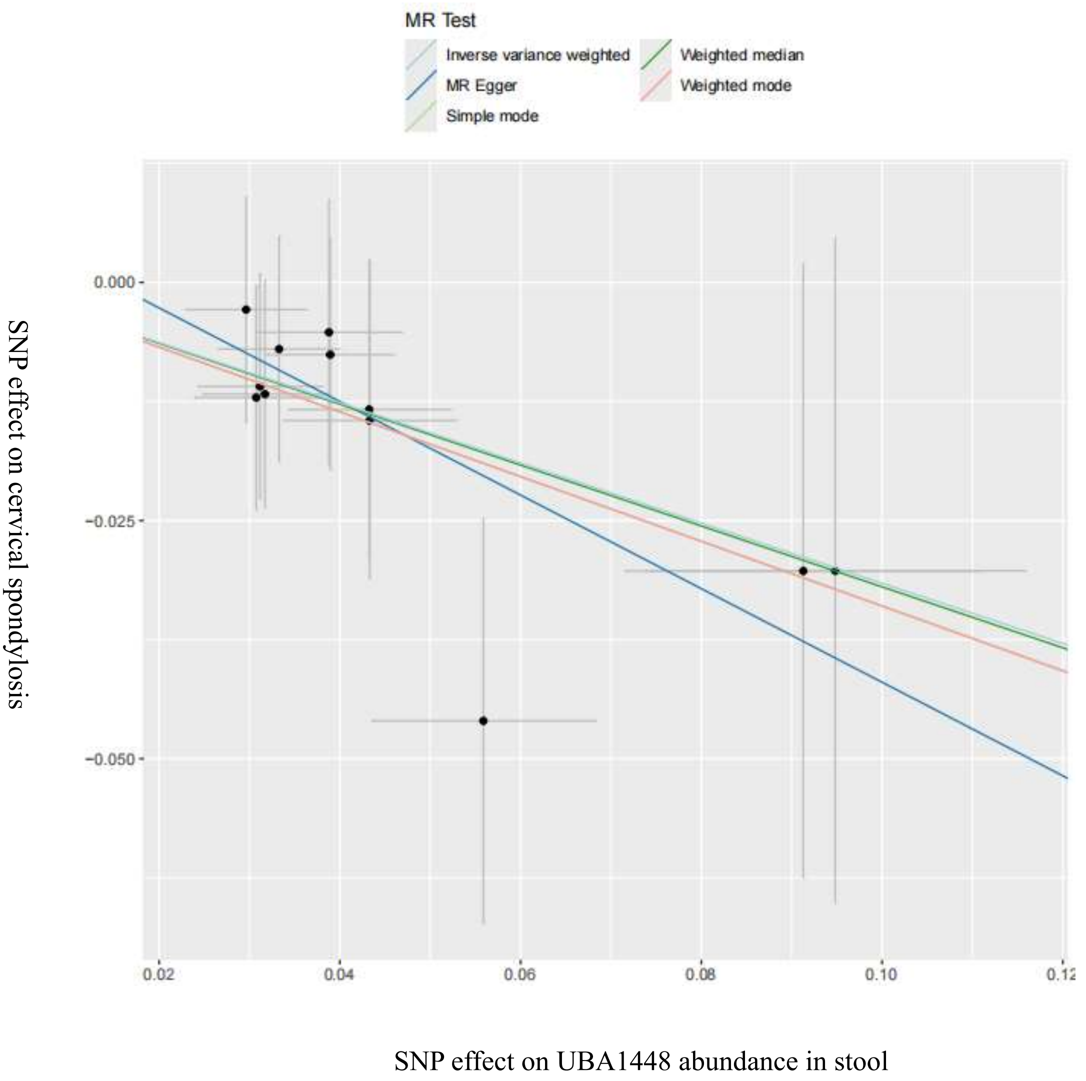
MR Test
Inverse variance weighted
MR Egger
Simple mode
Weighted median
Weighted mode
0.000
-0.025
-0.050
0.02
0.04
0.06
0.08
0.10
0.12
SNP effect on cervical spondylosis
SNP effect on UBA1448 abundance in stool

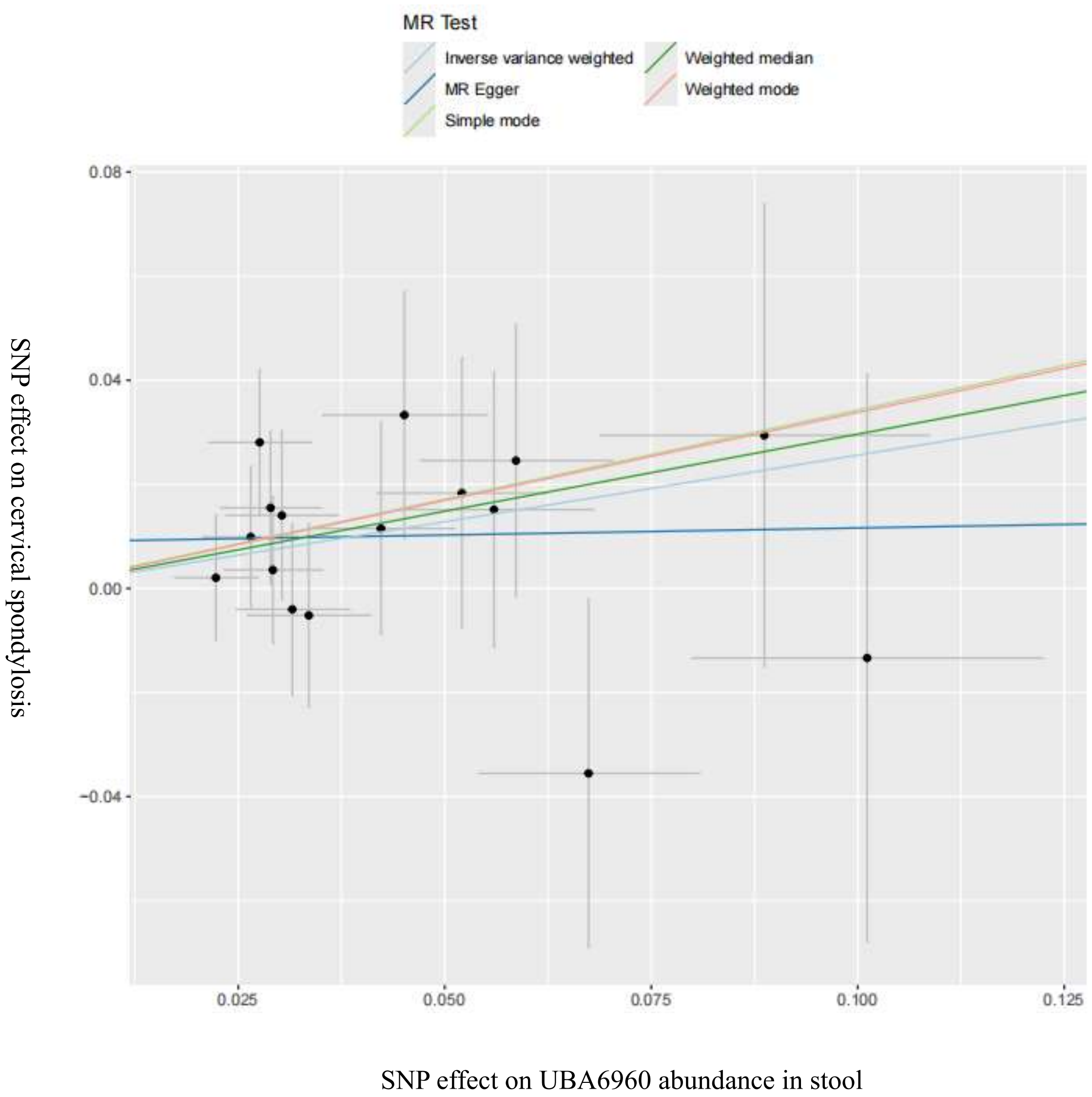
MR Test
Inverse variance weighted
MR Egger
Simple mode
Weighted median
Weighted mode
0.08
0.04
0.00
−0.04
0.025
0.050
0.075
0.100
0.125
SNP effect on cervical spondylosis
SNP effect on UBA6960 abundance in stool

Supplementary Figure S2. Mendelian randomization“leave-one-out”sensitivity analysis of gut microbiota on cervical spondylosis

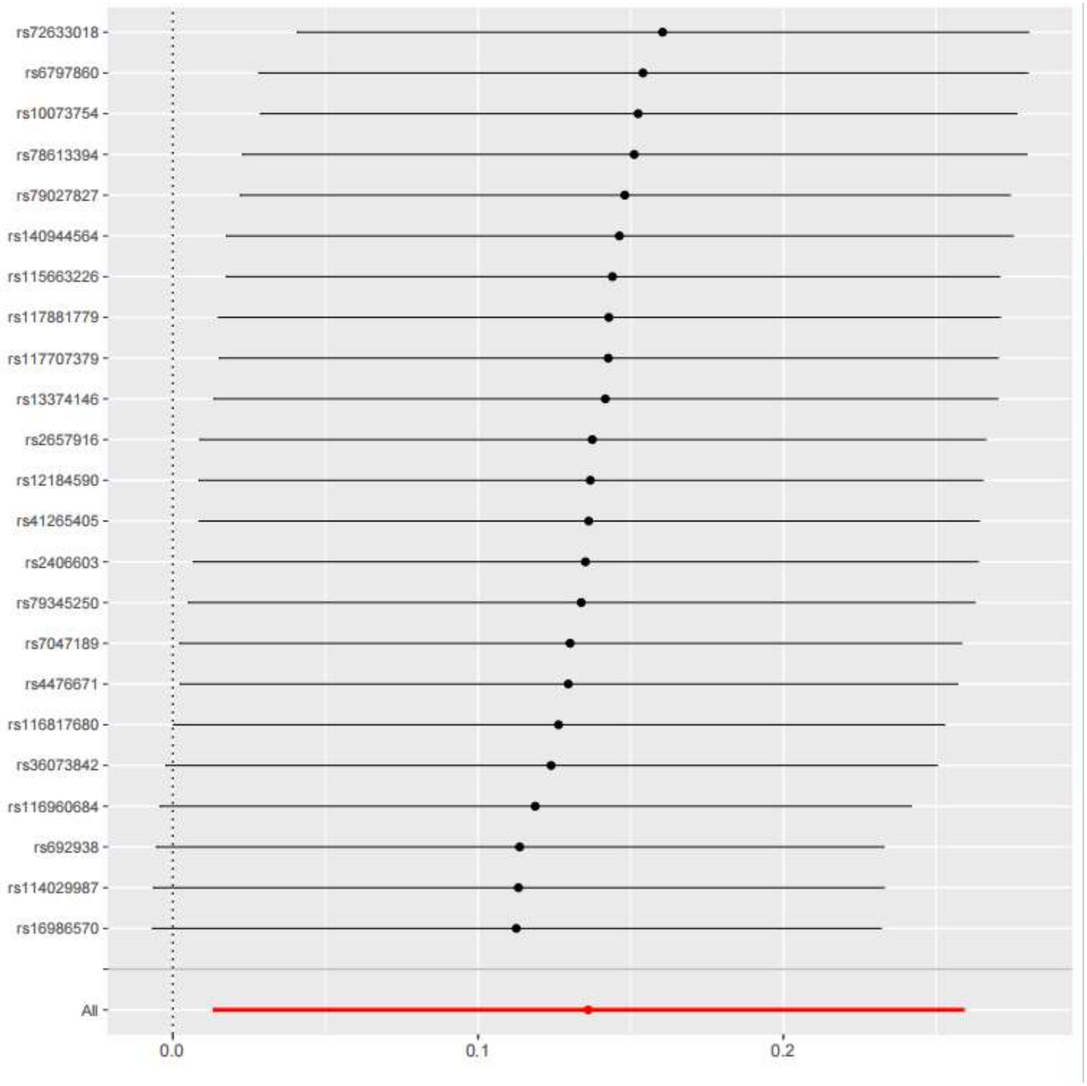

MR leave-one-out sensitivity analysis for Anaeromassilibacillus sp001305115 abundance in stool on cervical spondylosis

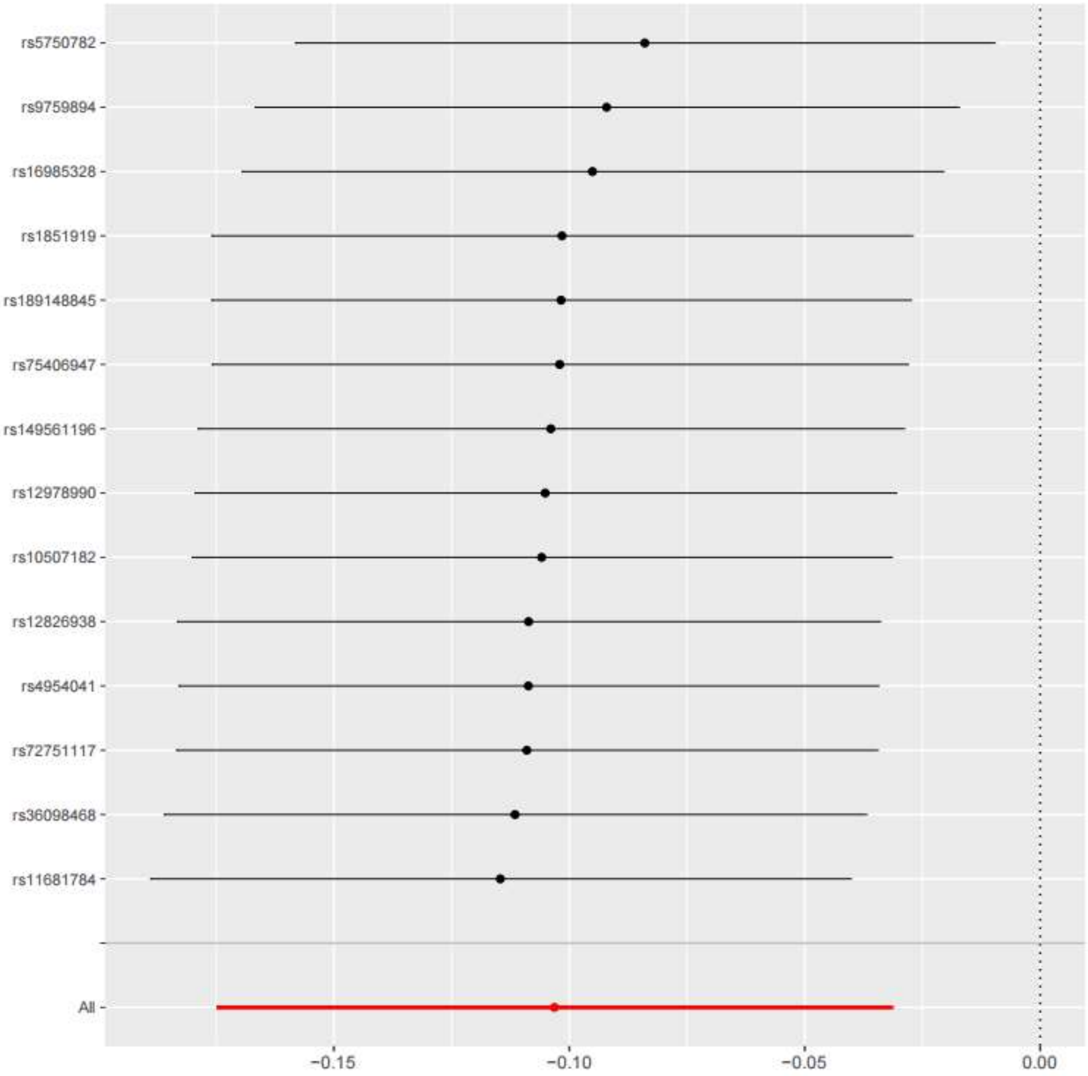

MR leave-one-out sensitivity analysis for Bacteroides A plebeius A abundance in stool on cervical spondylosis

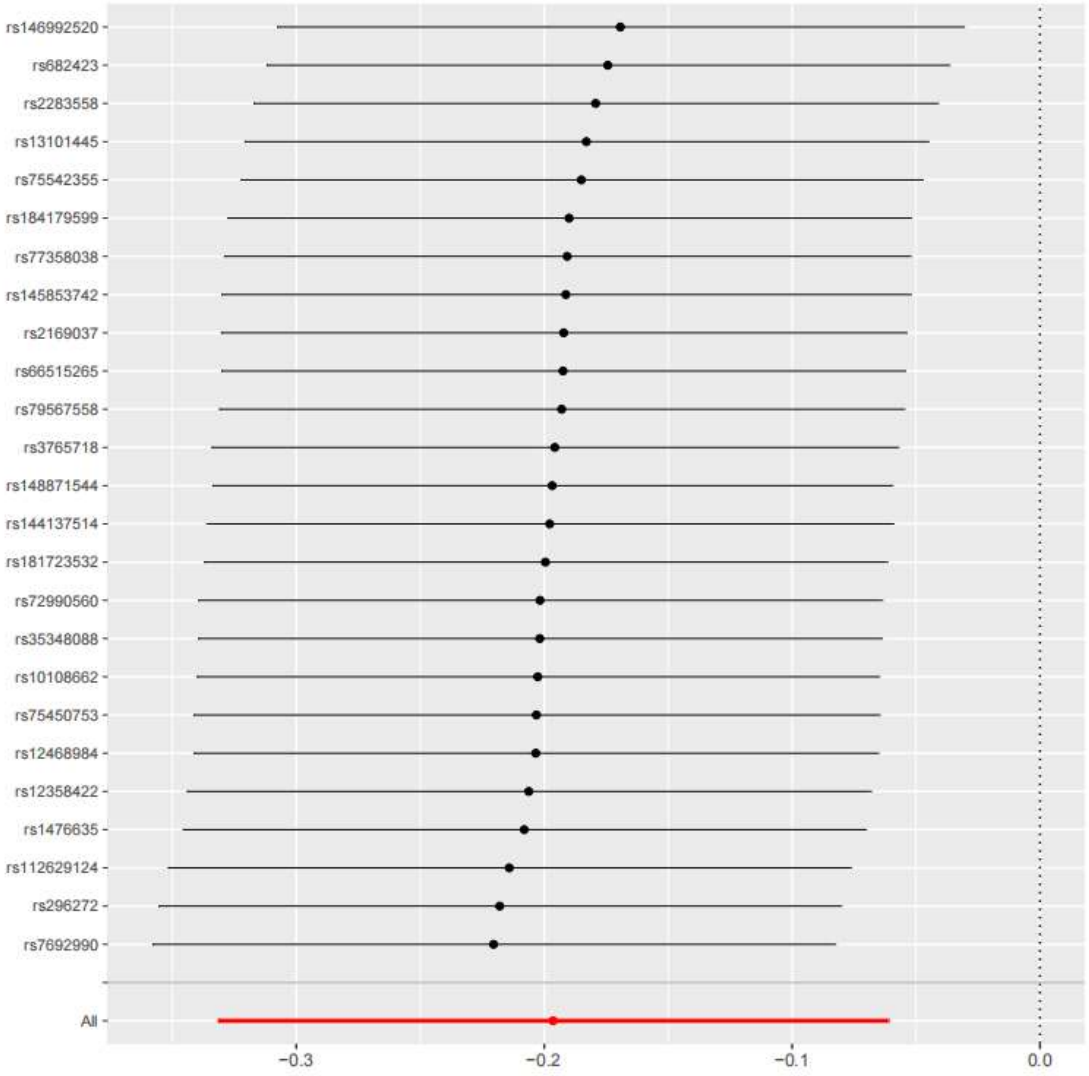

MR leave-one-out sensitivity analysis for Brachyspiraceae abundance in stool on cervical spondylosis

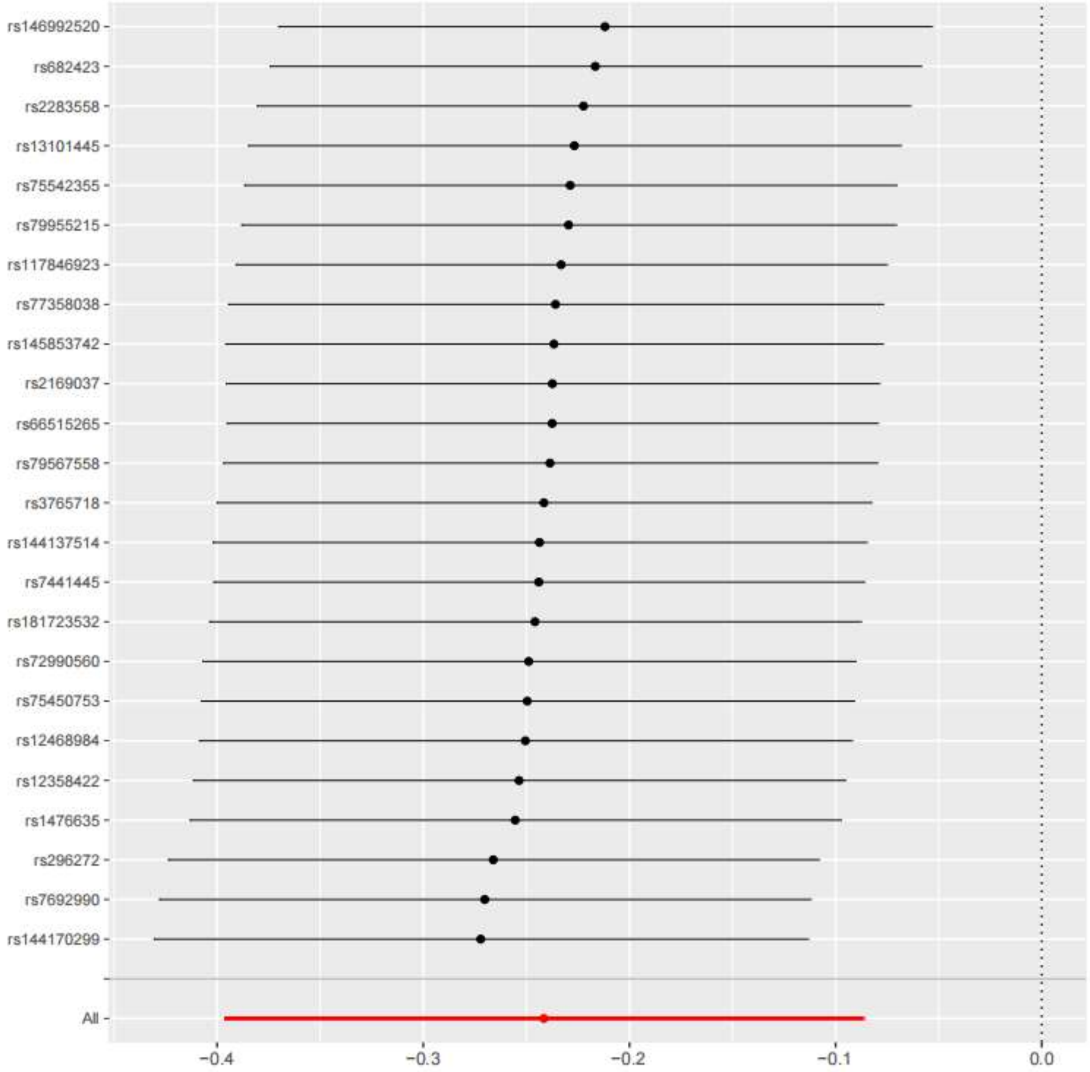

MR leave-one-out sensitivity analysis for Brachyspirae abundance in stool on cervical spondylosis

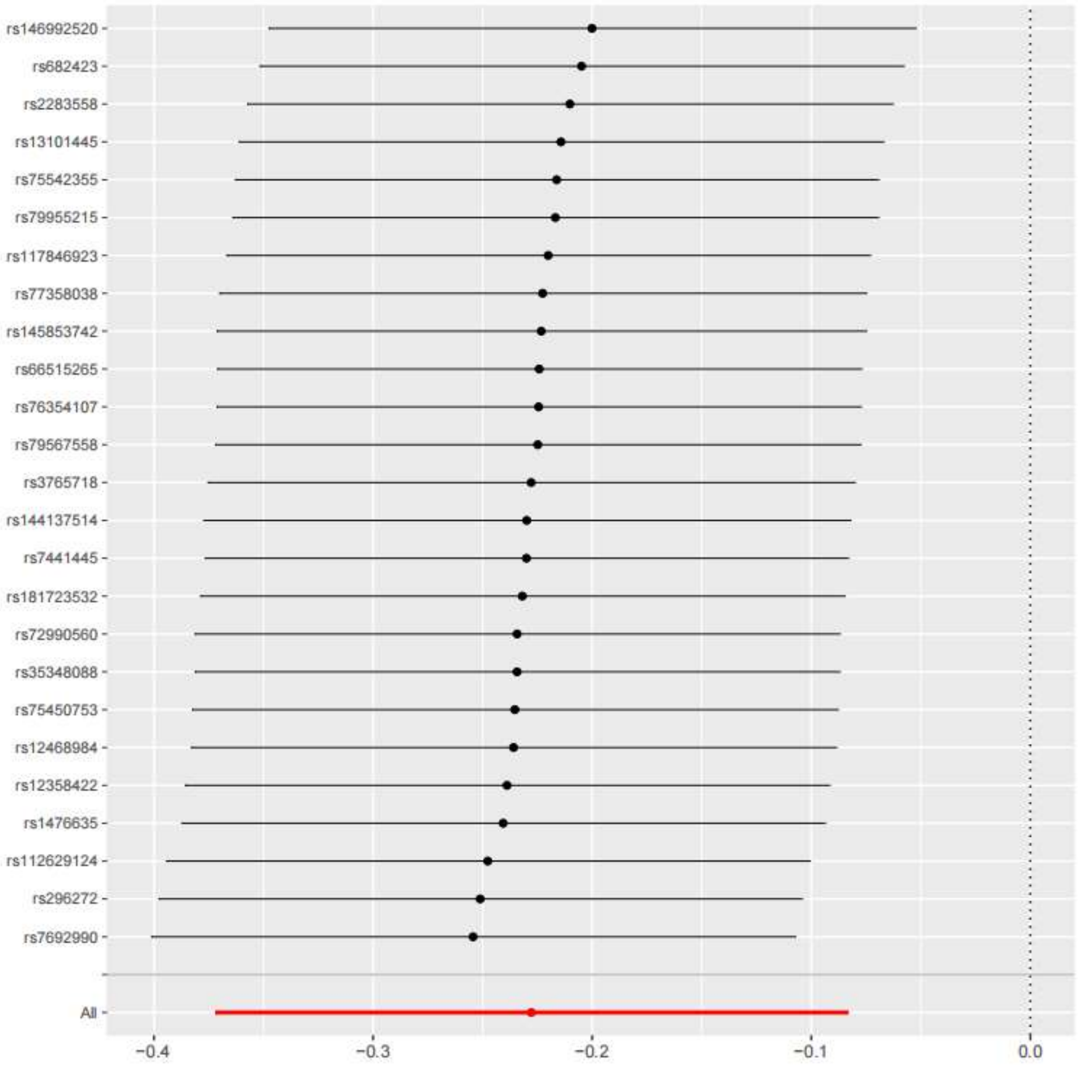

MR leave-one-out sensitivity analysis for Brachyspirales abundance in stool on cervical spondylosis

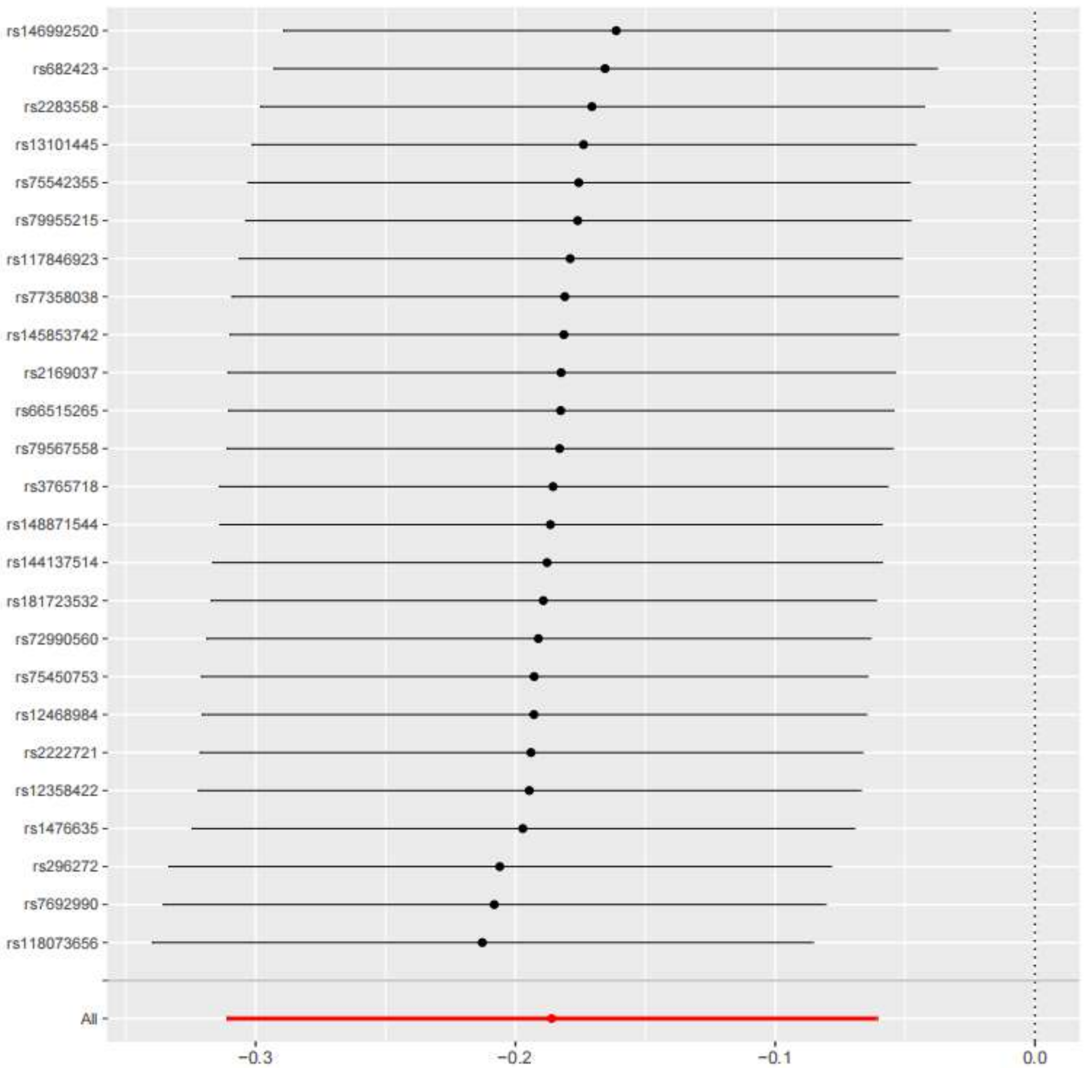

MR leave-one-out sensitivity analysis for Brachyspira abundance in stool on cervical spondylosis

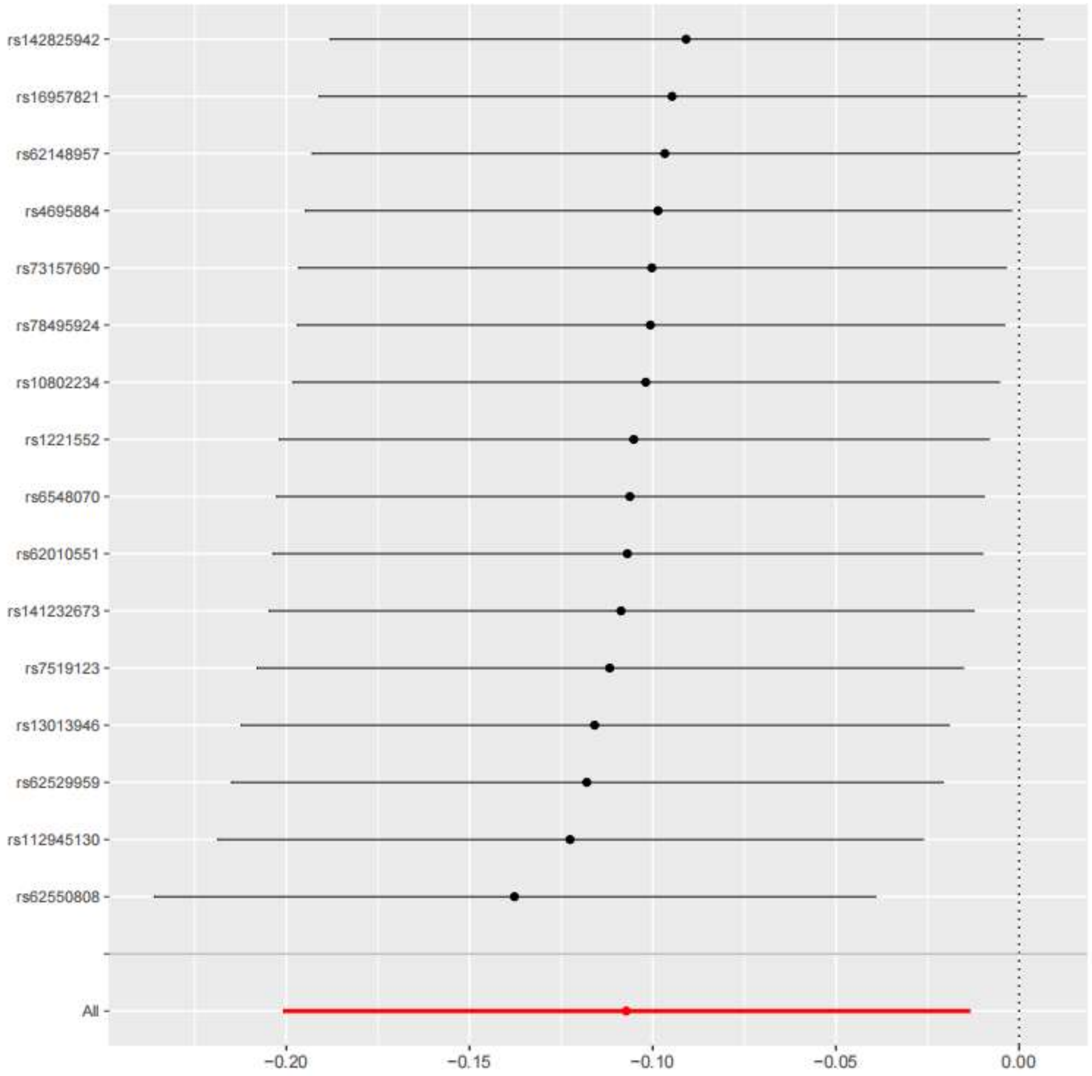

MR leave-one-out sensitivity analysis for CAG-110 abundance in stool on cervical spondylosis

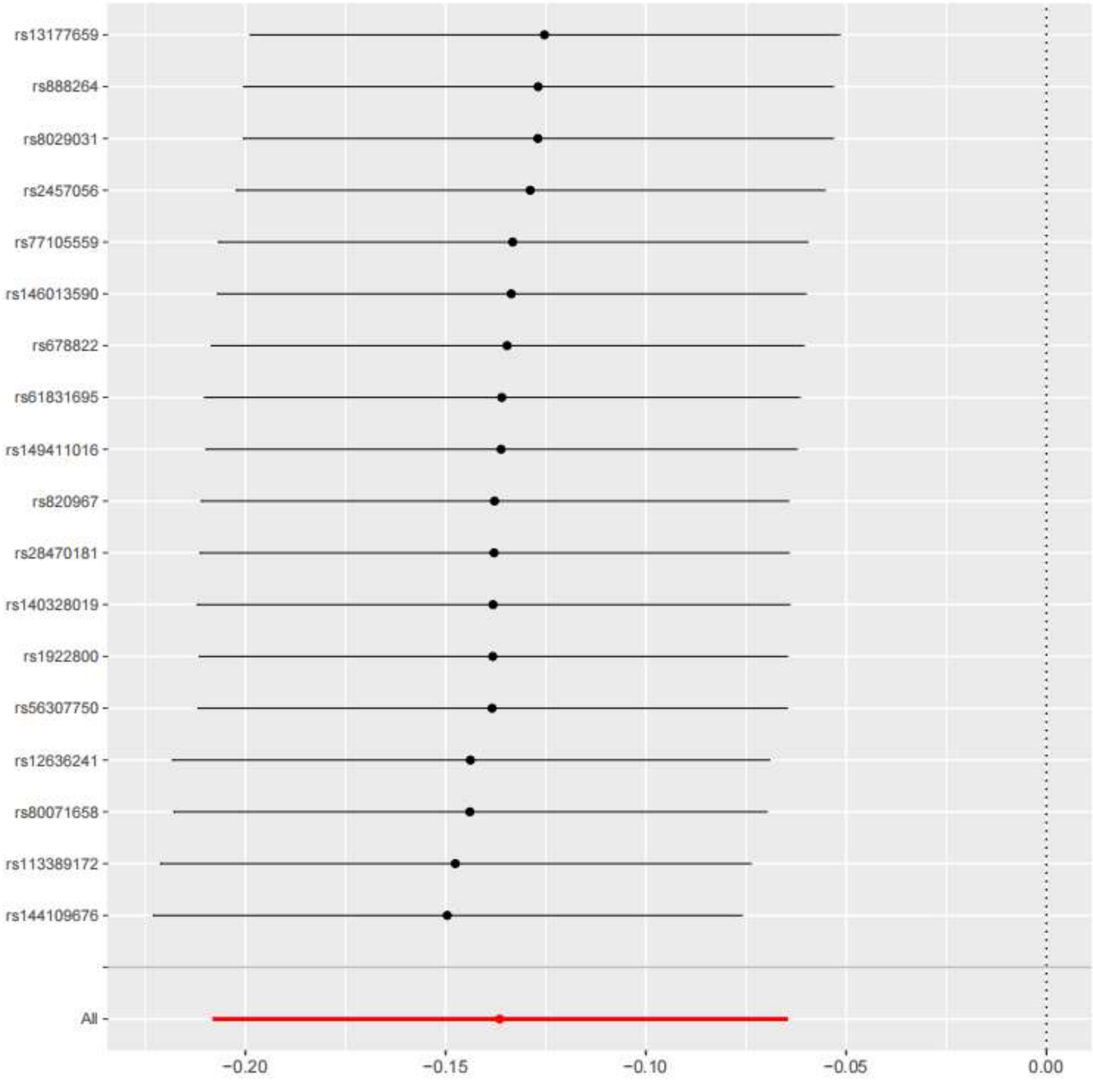

MR leave-one-out sensitivity analysis for CAG-448 sp003150135 abundance in stool on cervical spondylosis

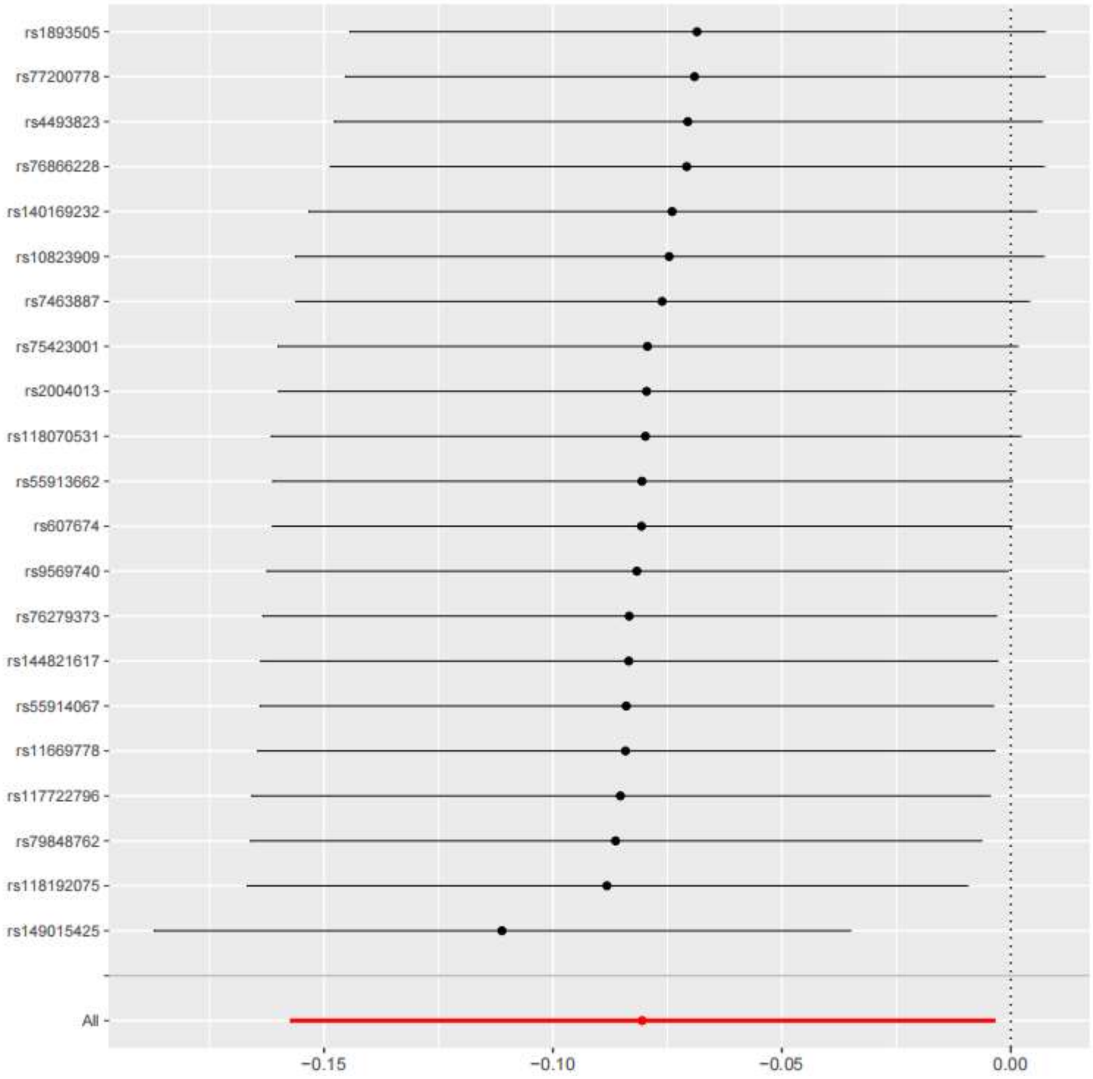

MR leave-one-out sensitivity analysis for CAG-776 abundance in stool on cervical spondylosis

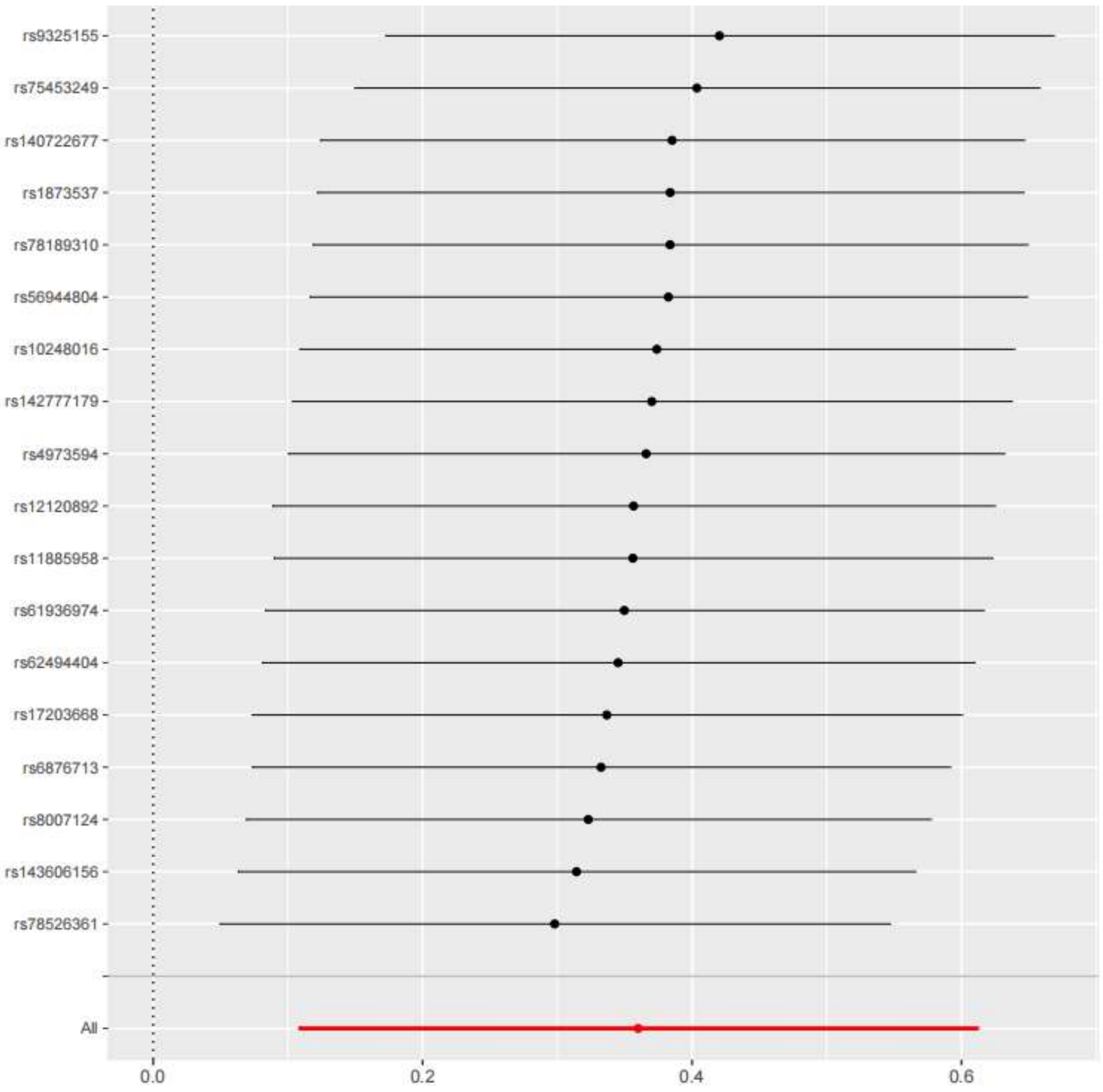

MR leave-one-out sensitivity analysis for Comamonas B abundance in stool on cervical spondylosis

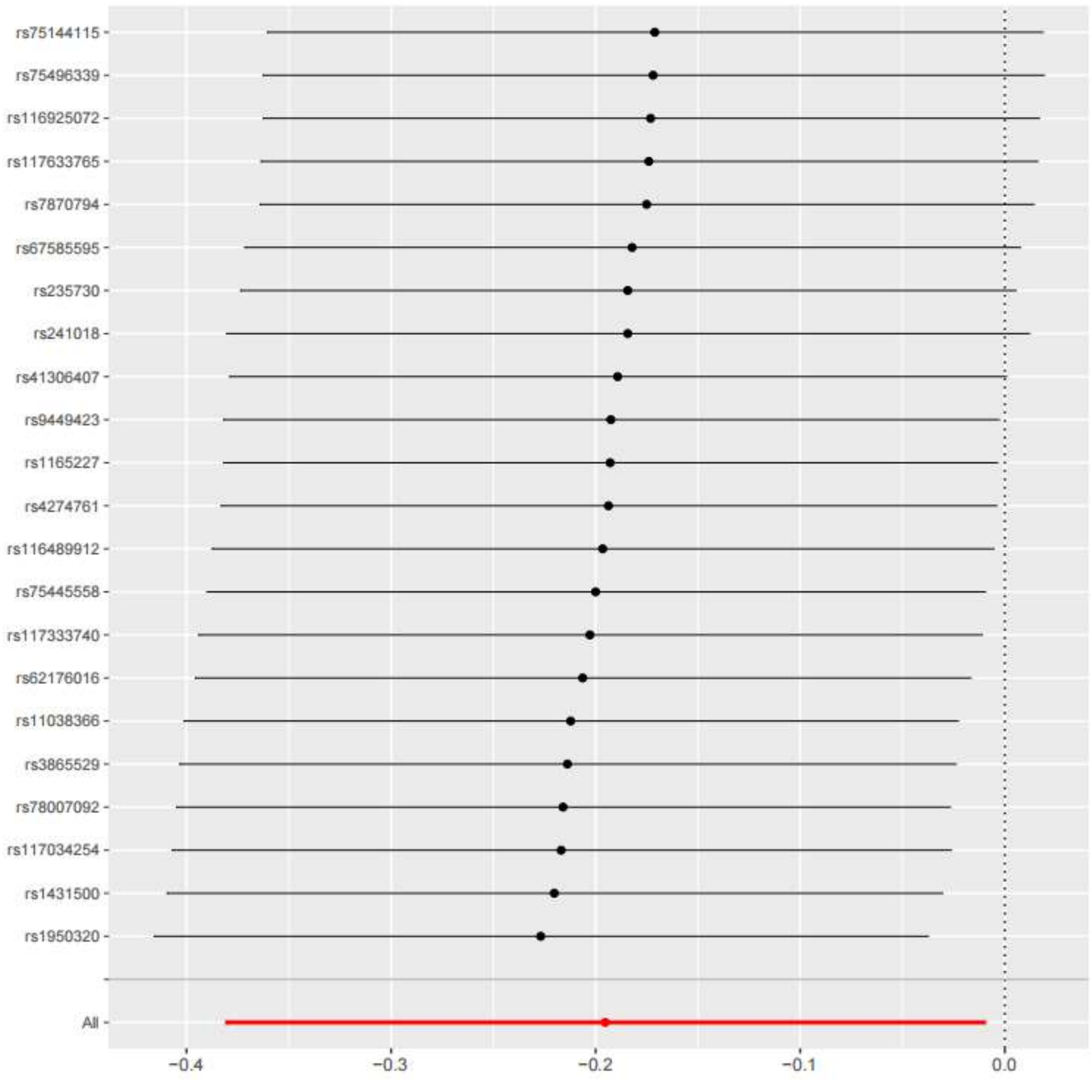

MR leave-one-out sensitivity analysis for Cyanobacteria abundance in stool on cervical spondylosis

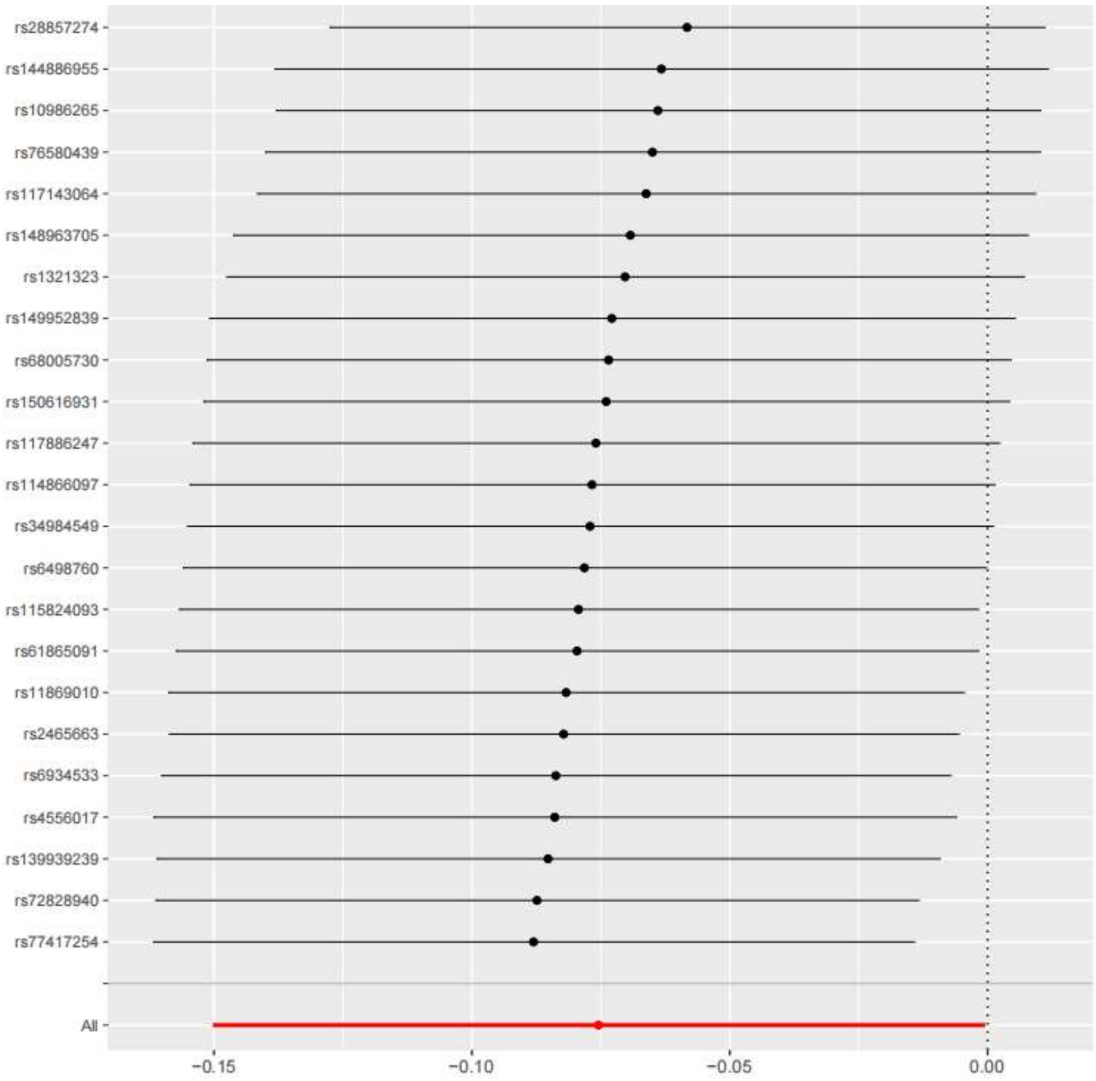

MR leave-one-out sensitivity analysis for Escherichia flexneri abundance in stool on cervical spondylosis

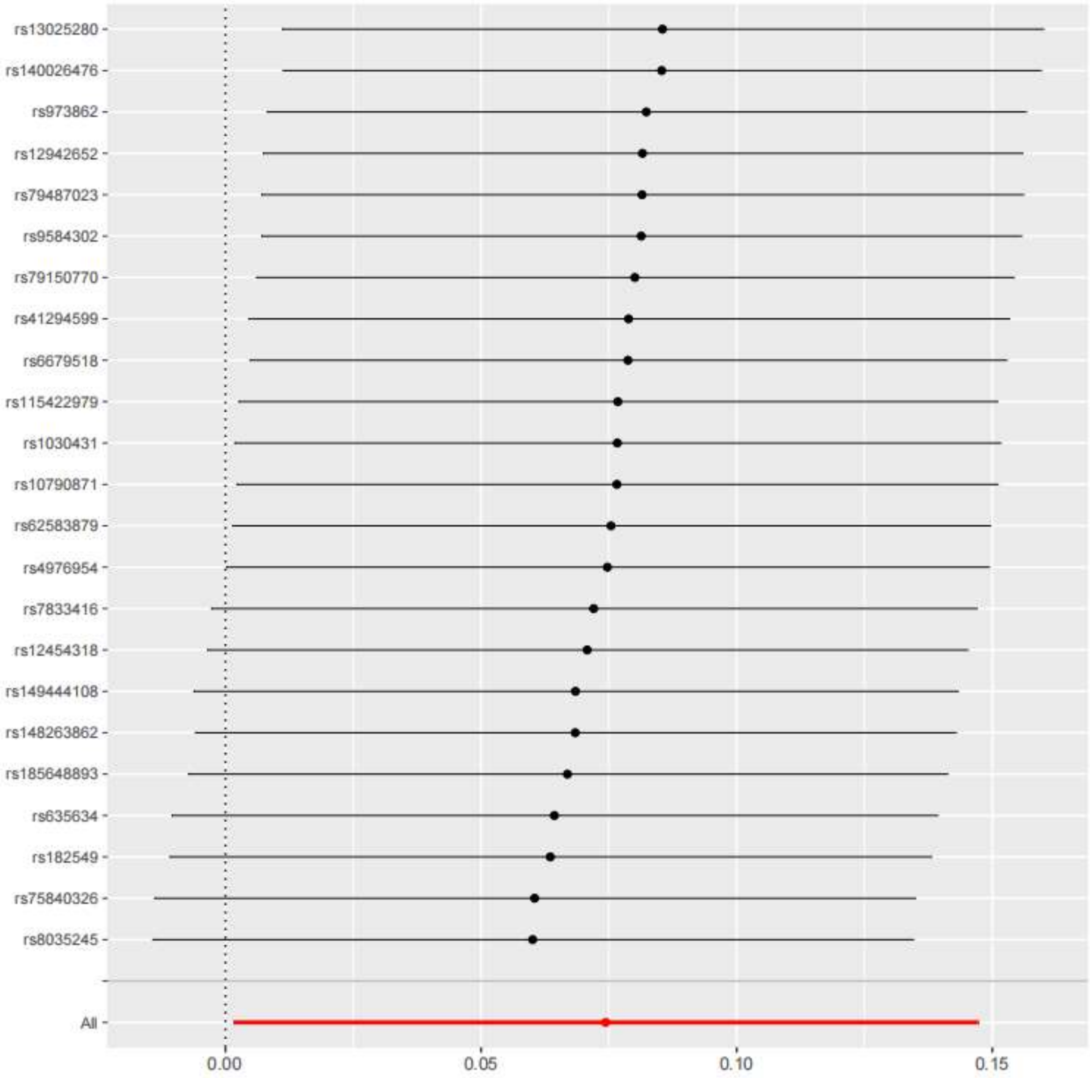

MR leave-one-out sensitivity analysis for Faecalicatena torques abundance in stool on cervical spondylosis

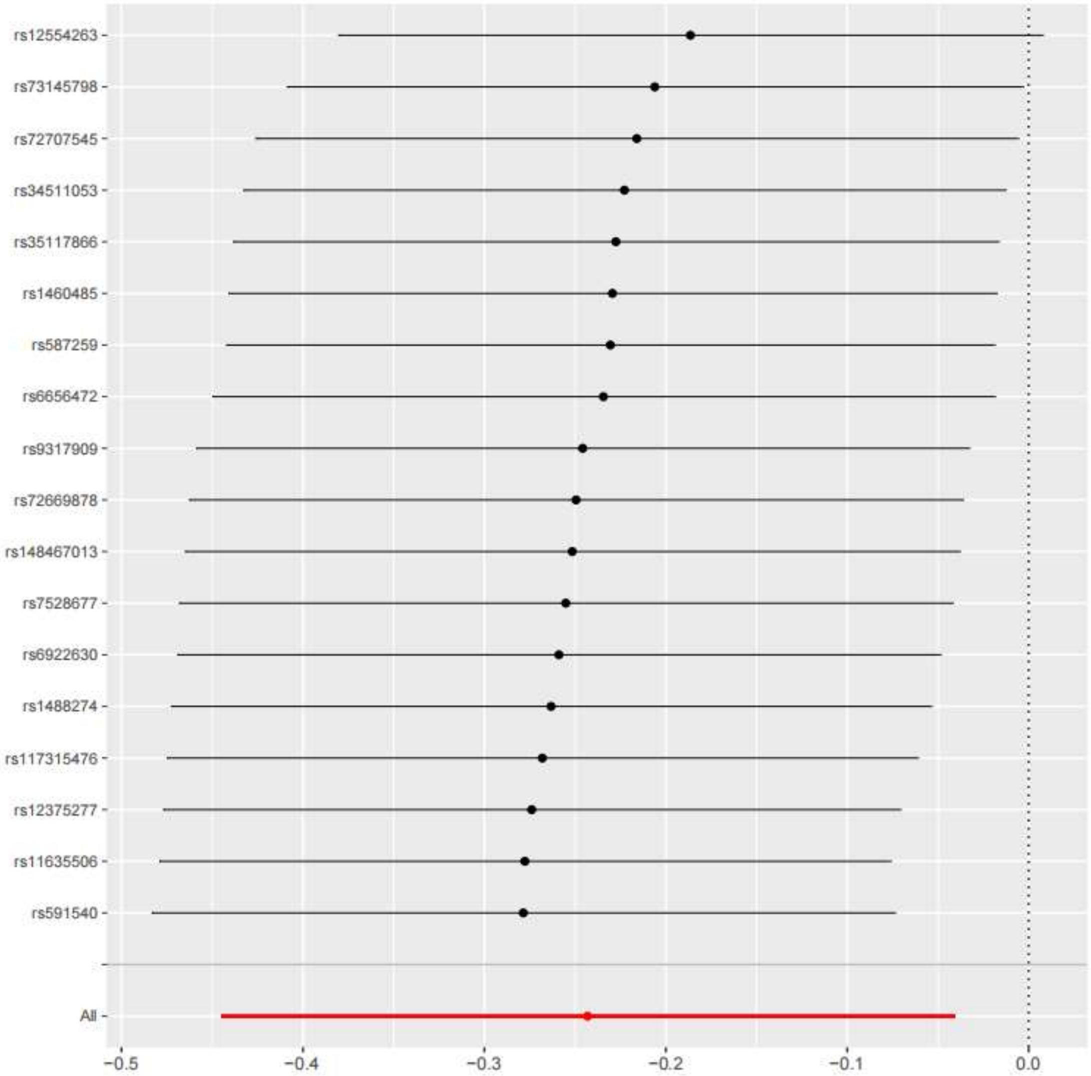

MR leave-one-out sensitivity analysis for Fournierella massiliensis abundance in stool on cervical spondylosis

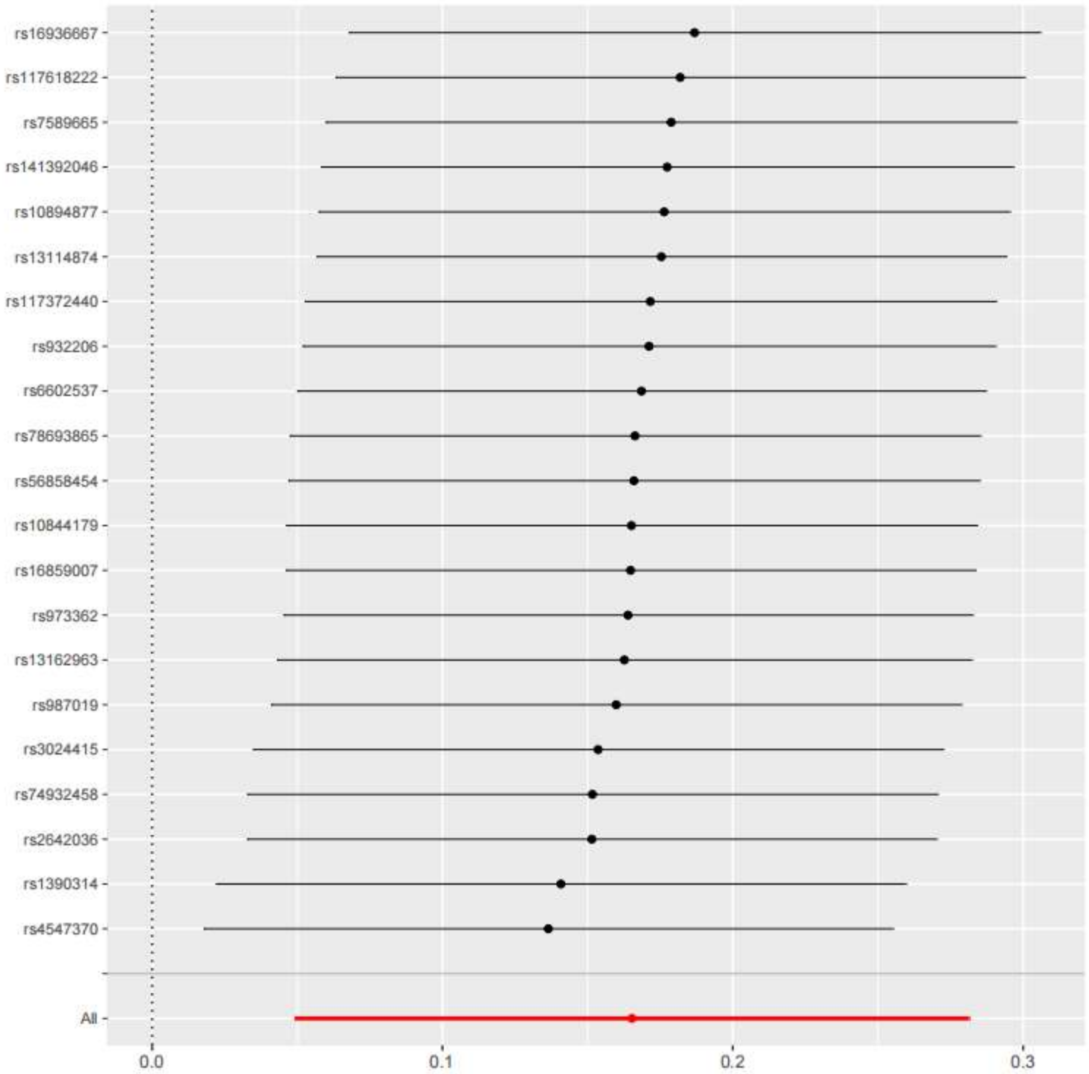

MR leave-one-out sensitivity analysis for GCA-900066495 sp900066495 abundance in stool on cervical spondylosis

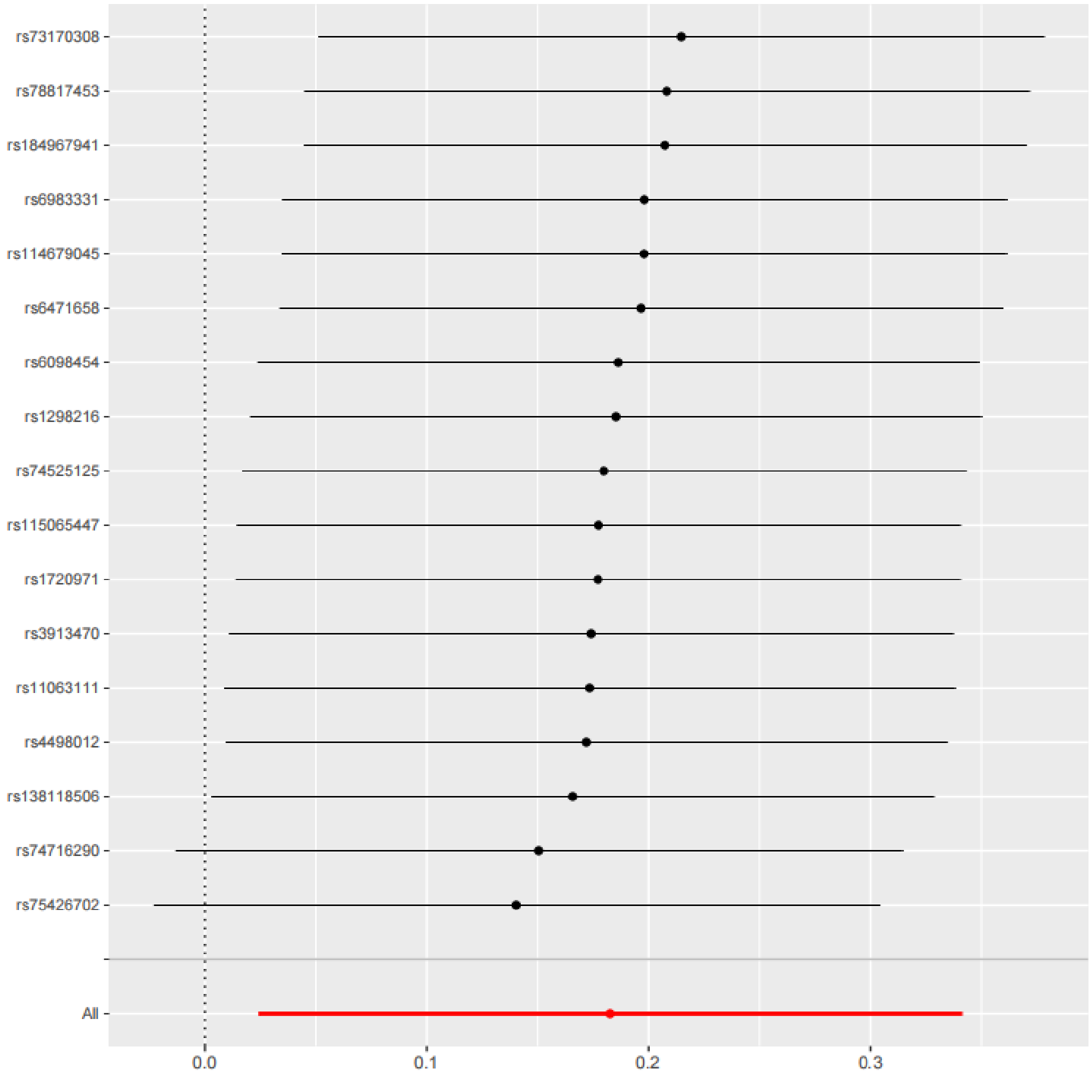

MR leave-one-out sensitivity analysis for Intestinimonas massiliensis abundance in stool on cervical spondylosis

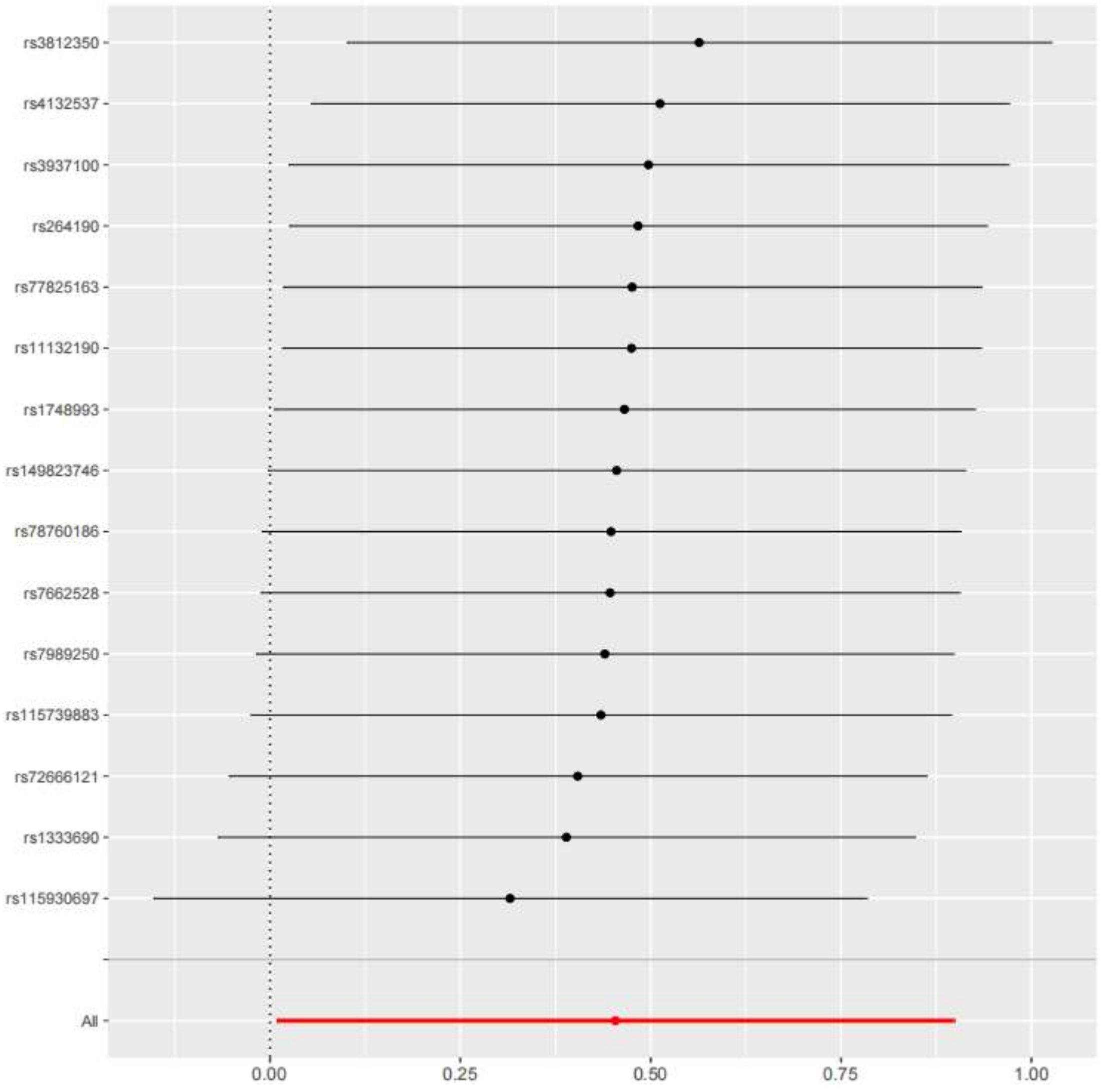

MR leave-one-out sensitivity analysis for koll11 abundance in stool on cervical spondylosis

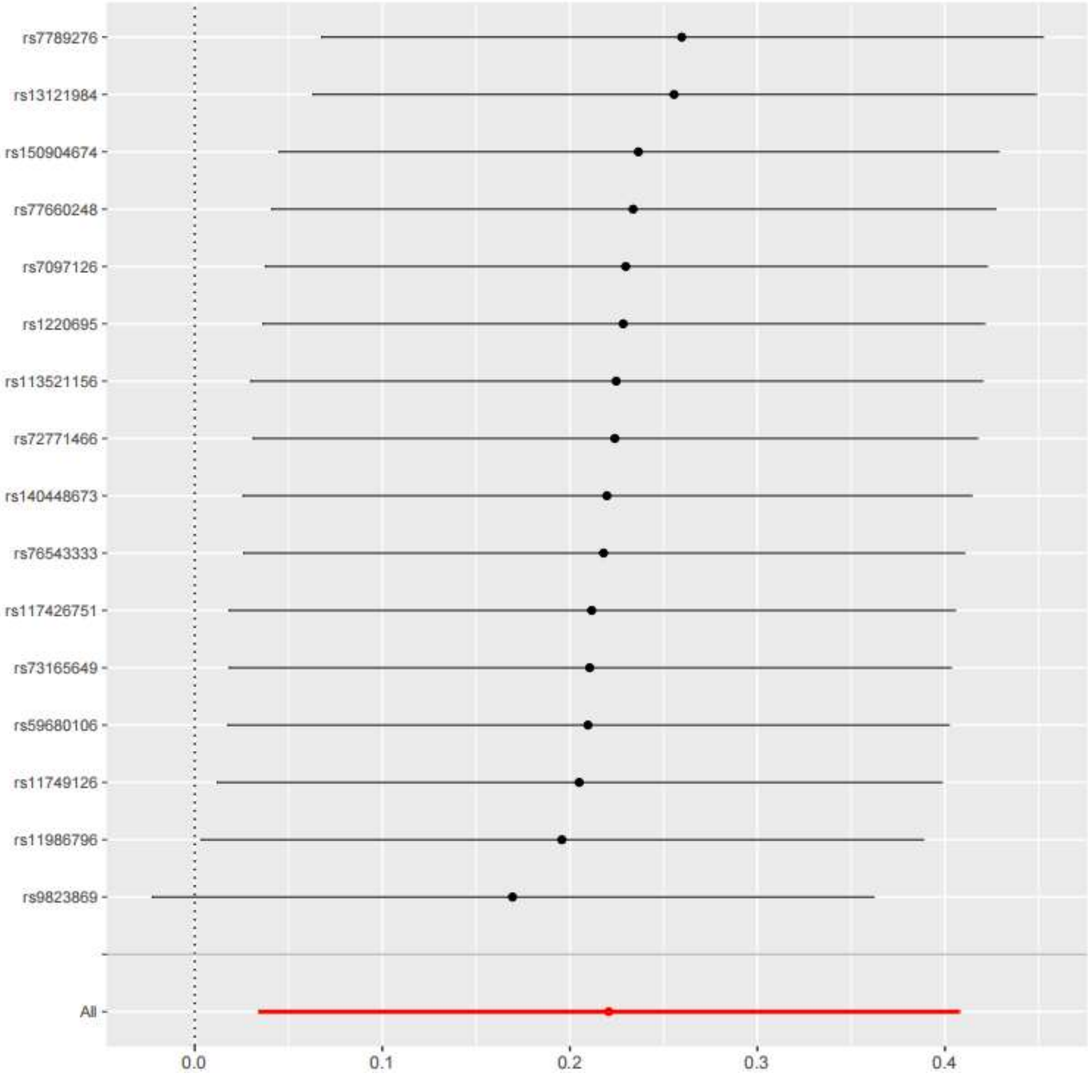

MR leave-one-out sensitivity analysis for Methanobacterium B abundance in stool on cervical spondylosis

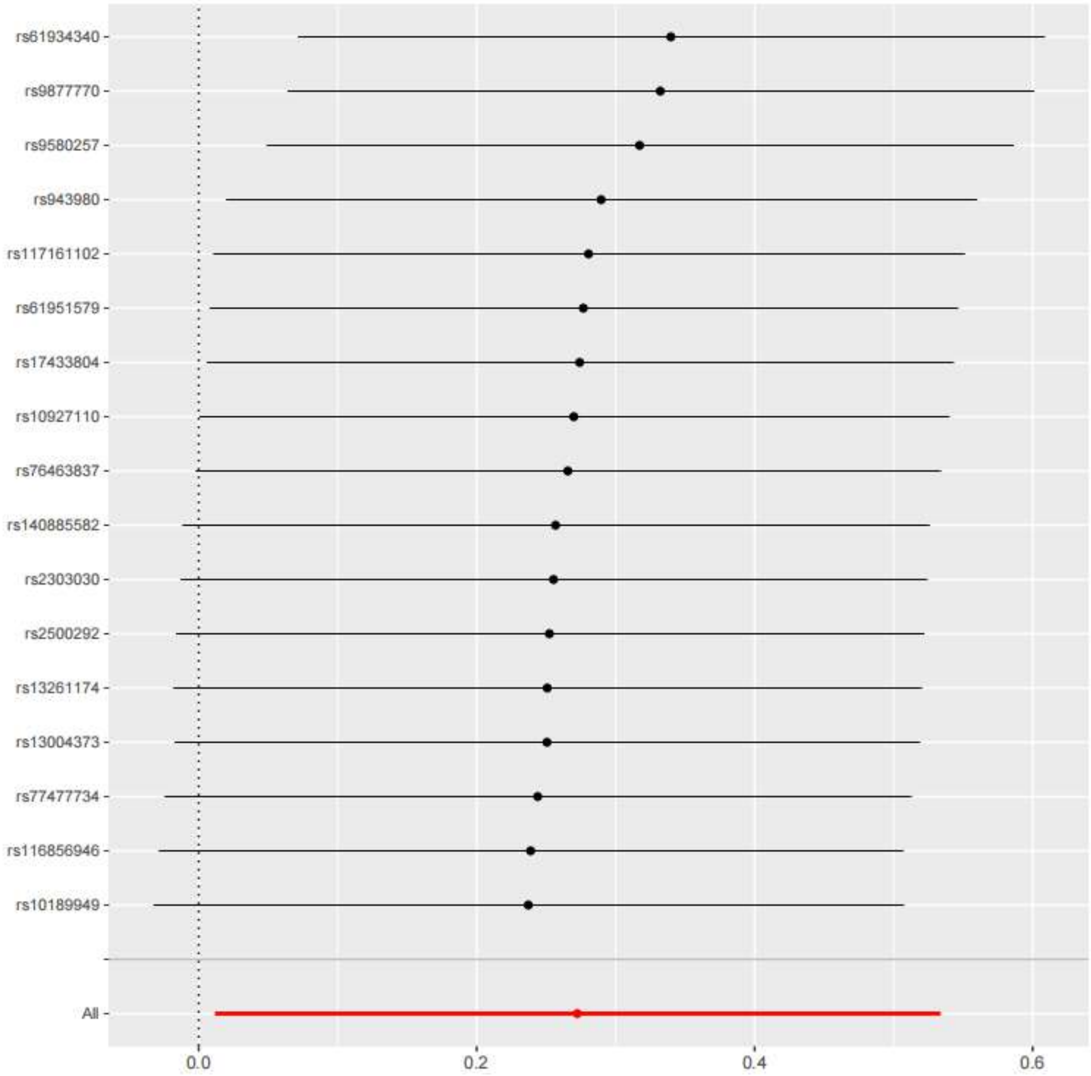

MR leave-one-out sensitivity analysis for NK4A144 abundance in stool on cervical spondylosis

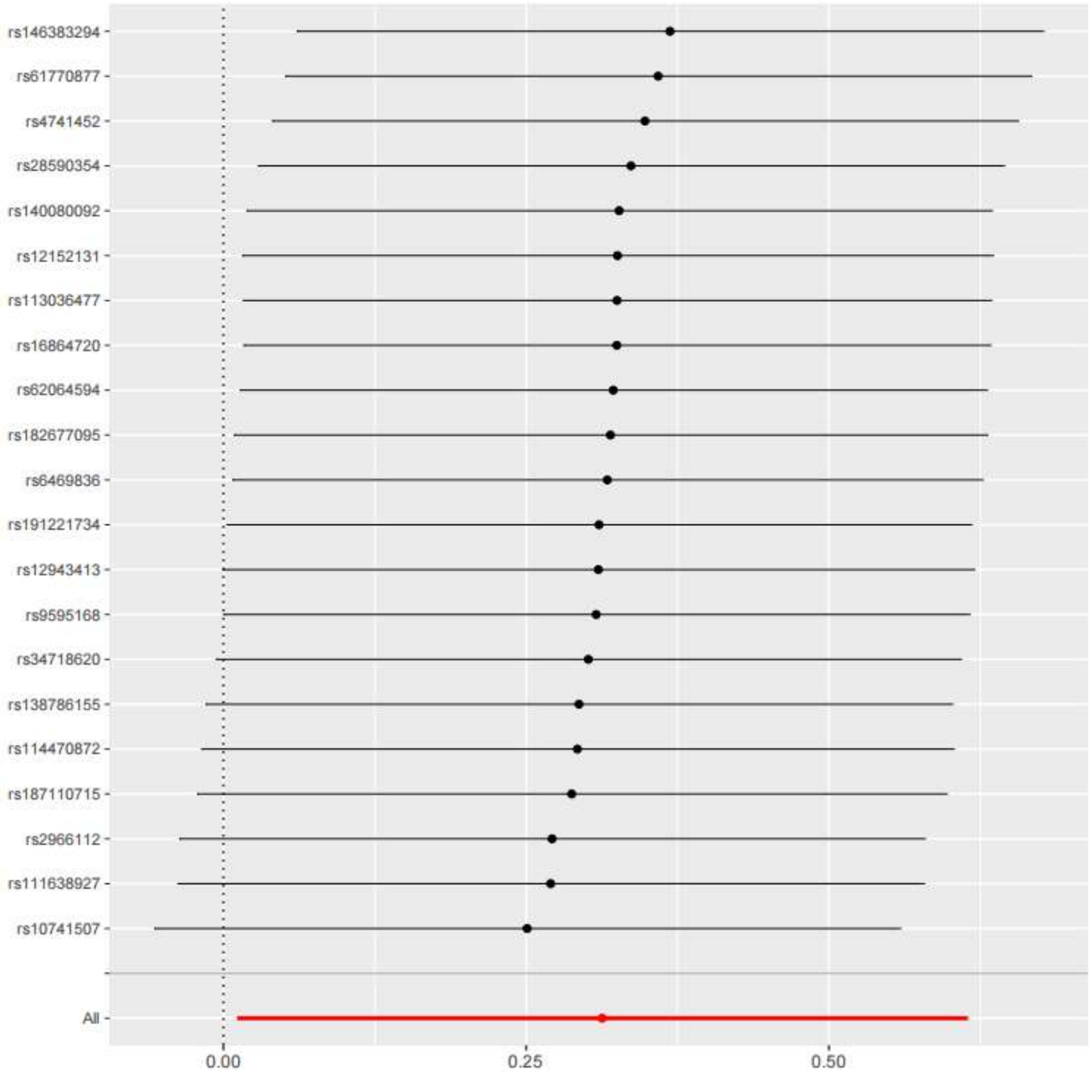

MR leave-one-out sensitivity analysis for Poseidoniaceae abundance in stool on cervical spondylosis

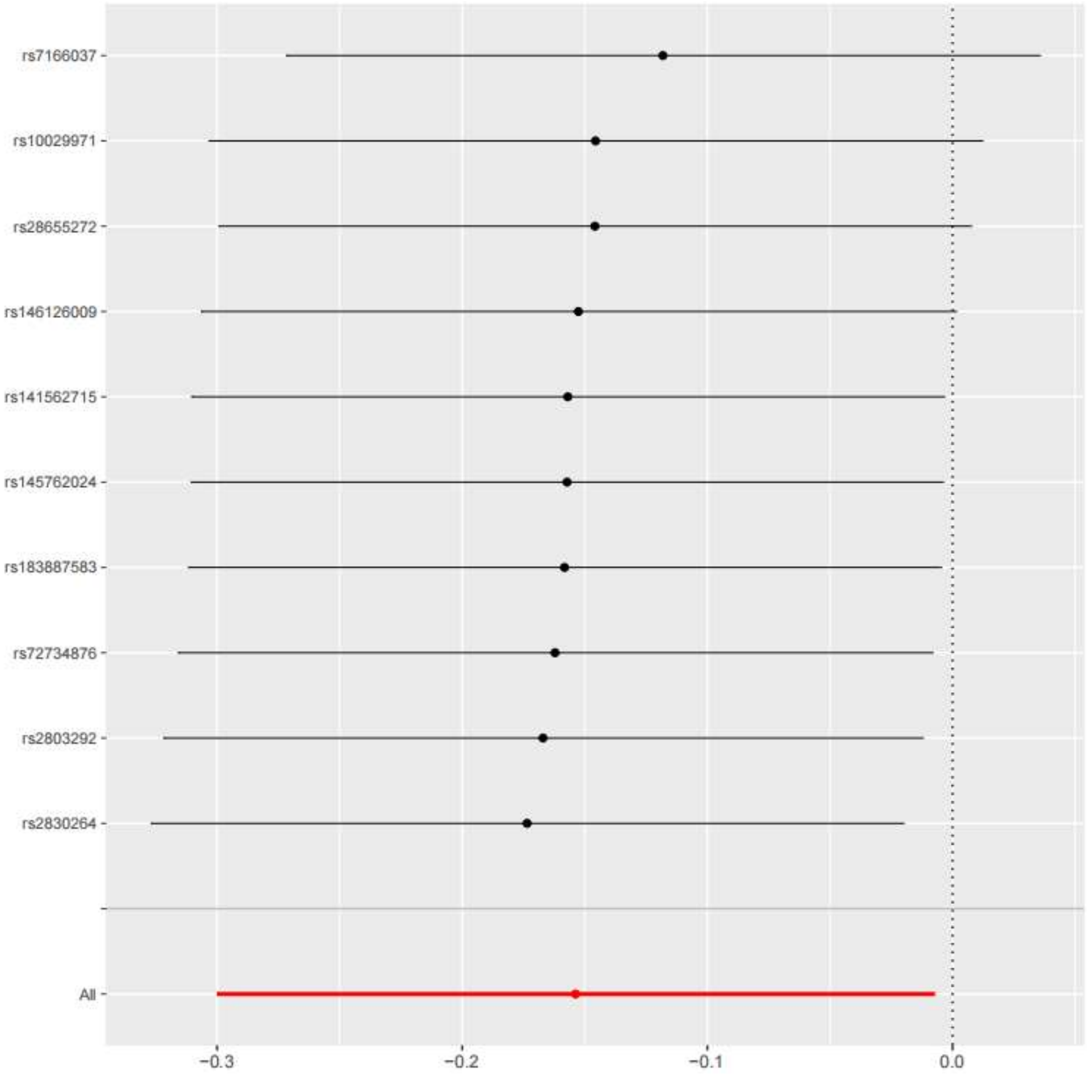

MR leave-one-out sensitivity analysis for Prevotella sp900318625 abundance in stool on cervical spondylosis

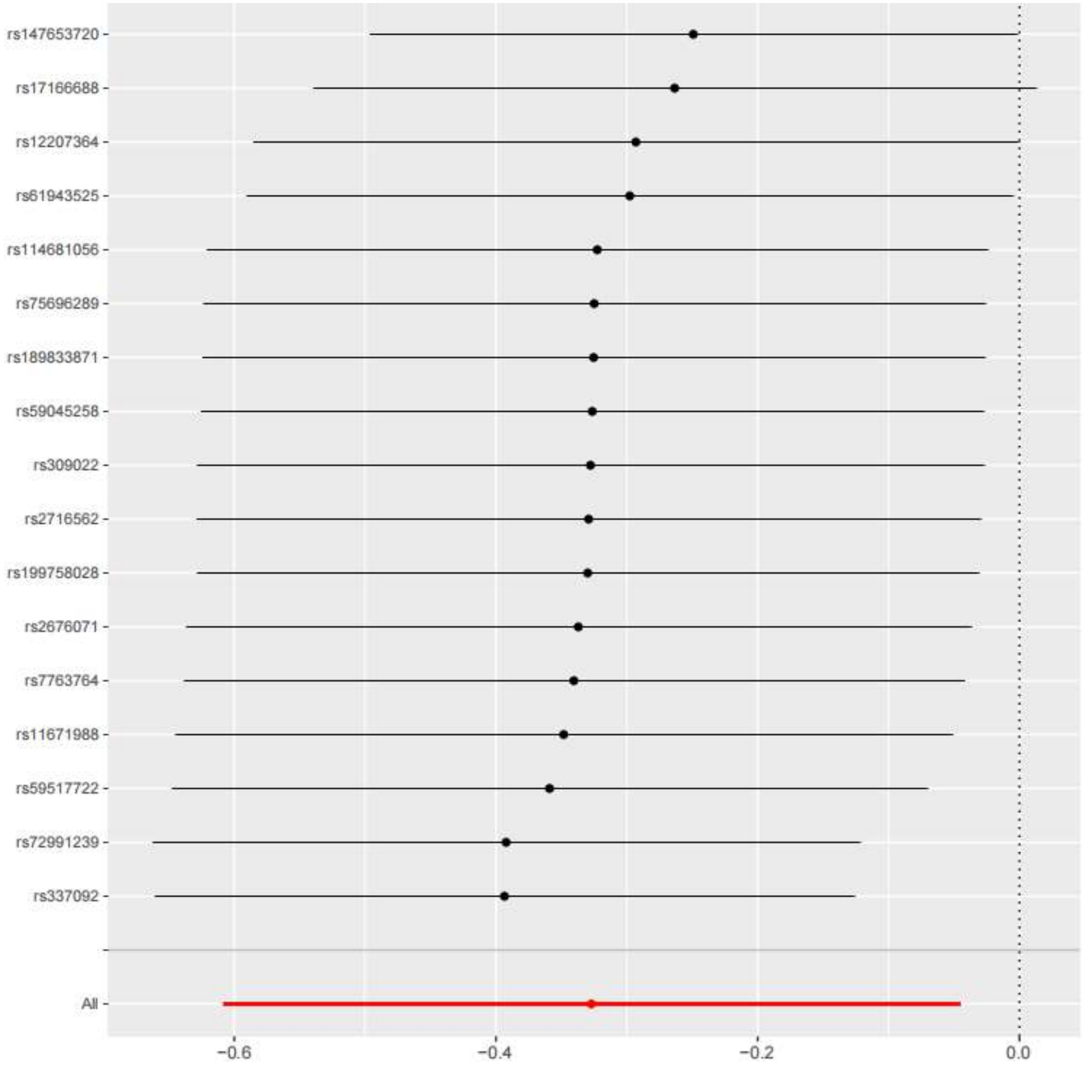

MR leave-one-out sensitivity analysis for RUG420 sp900317985 abundance in stool on cervical spondylosis

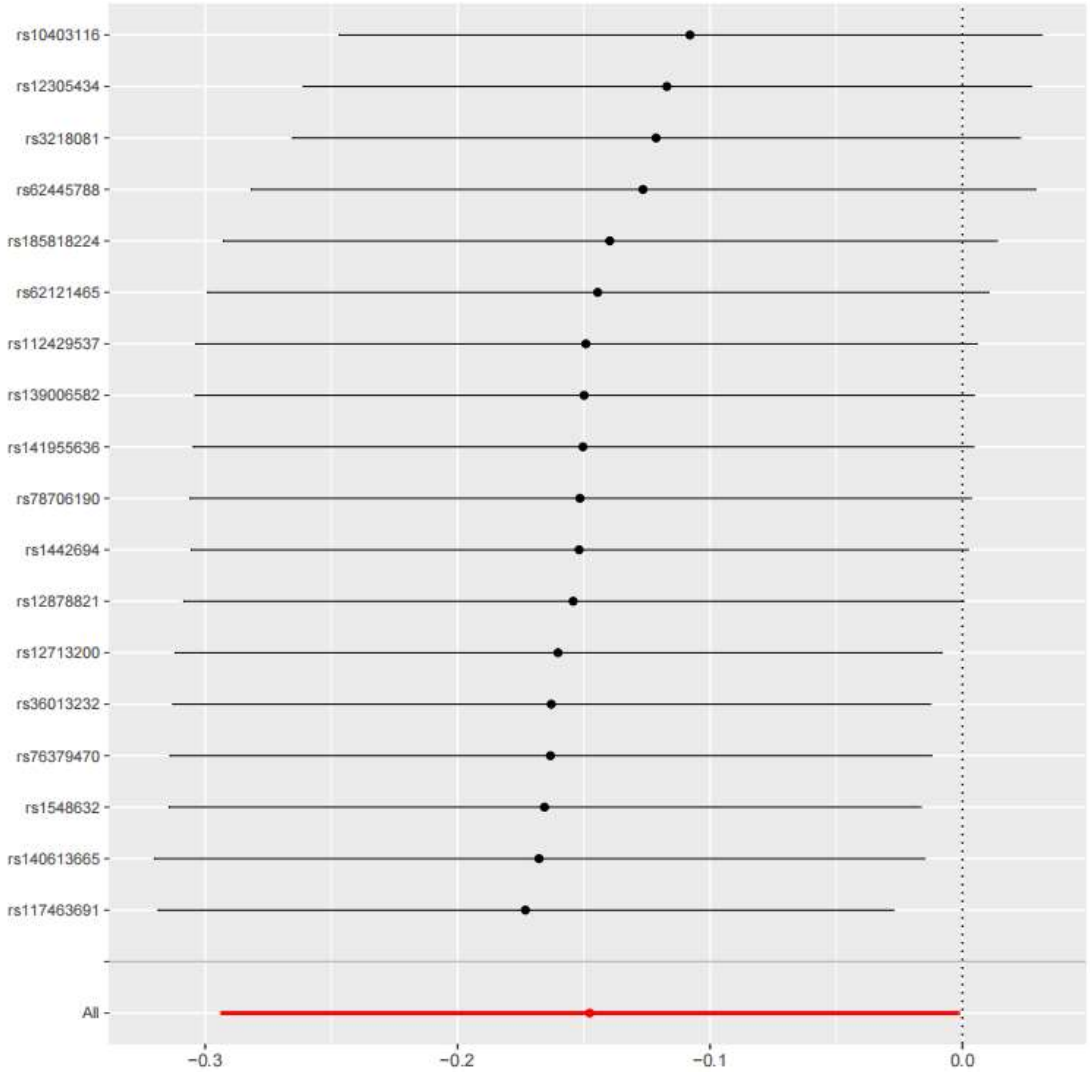

MR leave-one-out sensitivity analysis for Ruminococcus A sp000432335 abundance in stool on cervical spondylosis

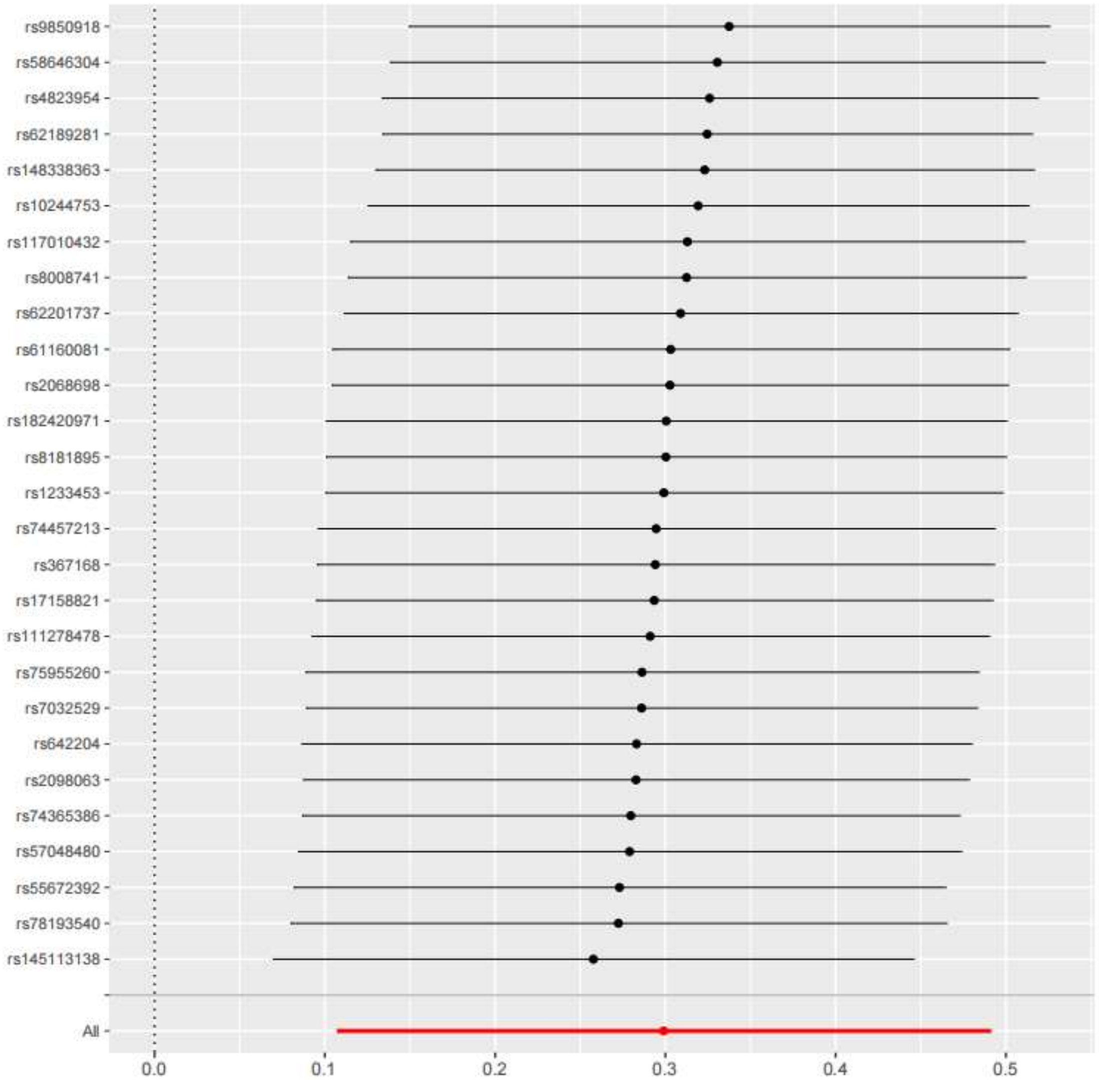

MR leave-one-out sensitivity analysis for Odoribacter laneus abundance in stool on cervical spondylosis

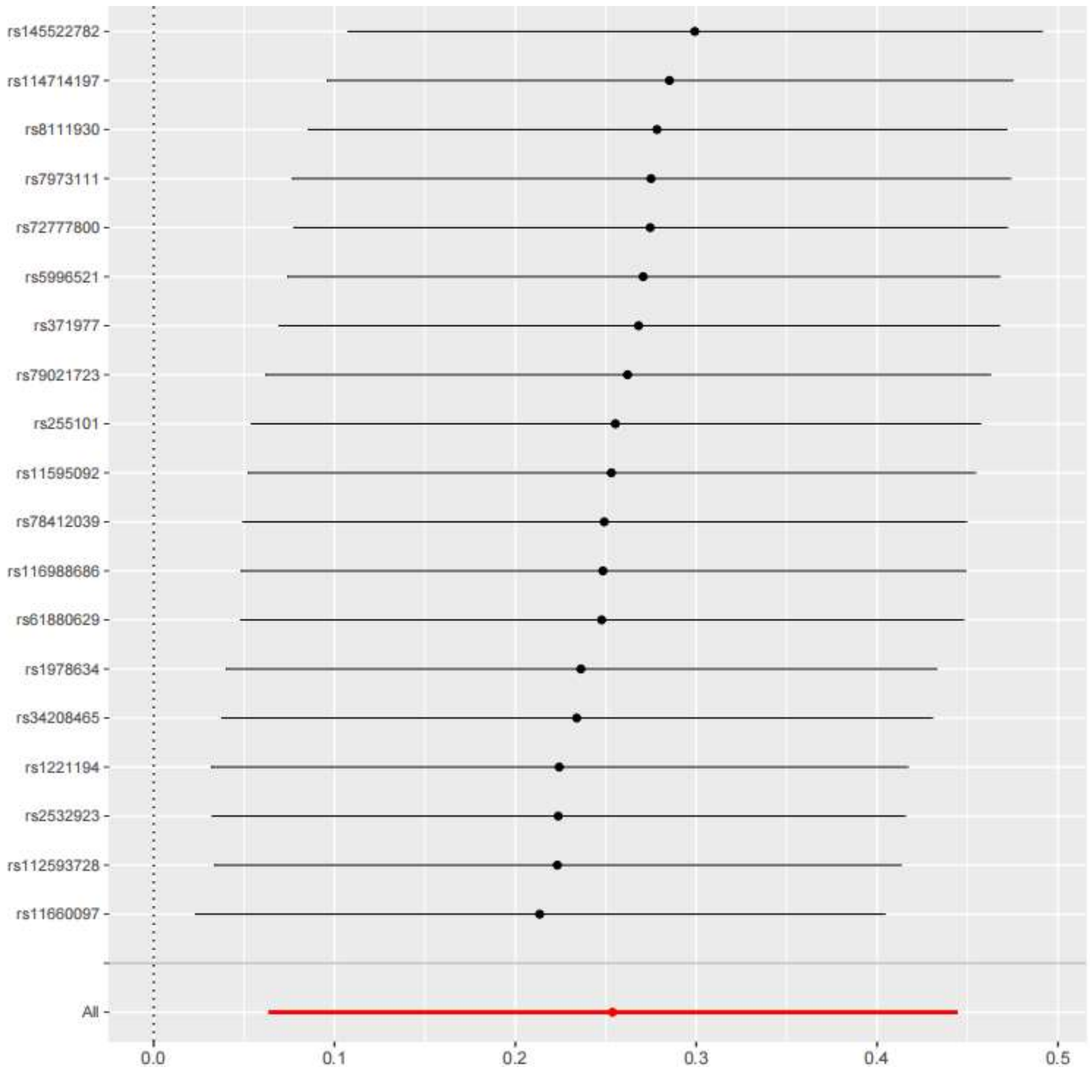

MR leave-one-out sensitivity analysis for TMED109 abundance in stool on cervical spondylosis

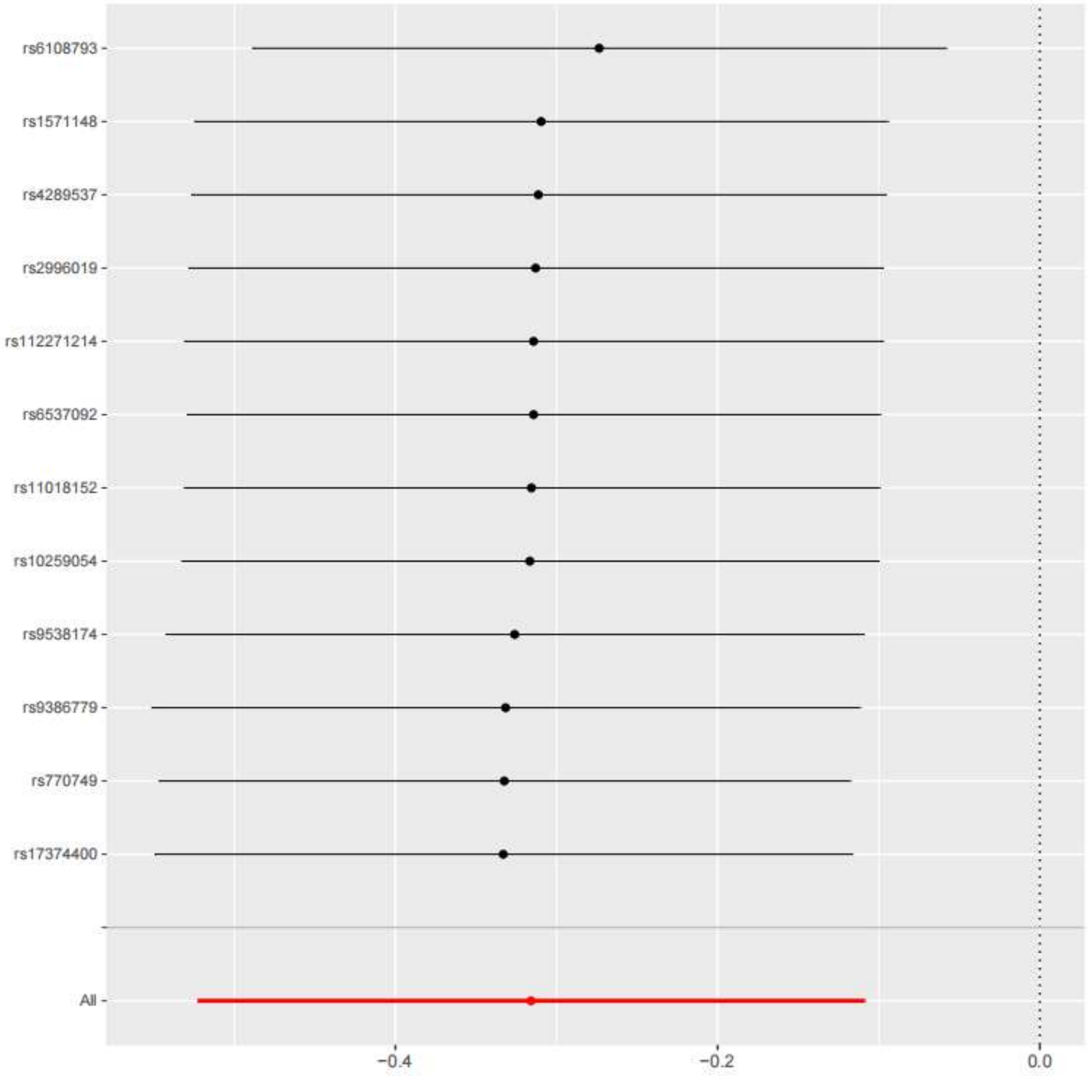

MR leave-one-out sensitivity analysis for UBA1448 abundance in stool on cervical spondylosis

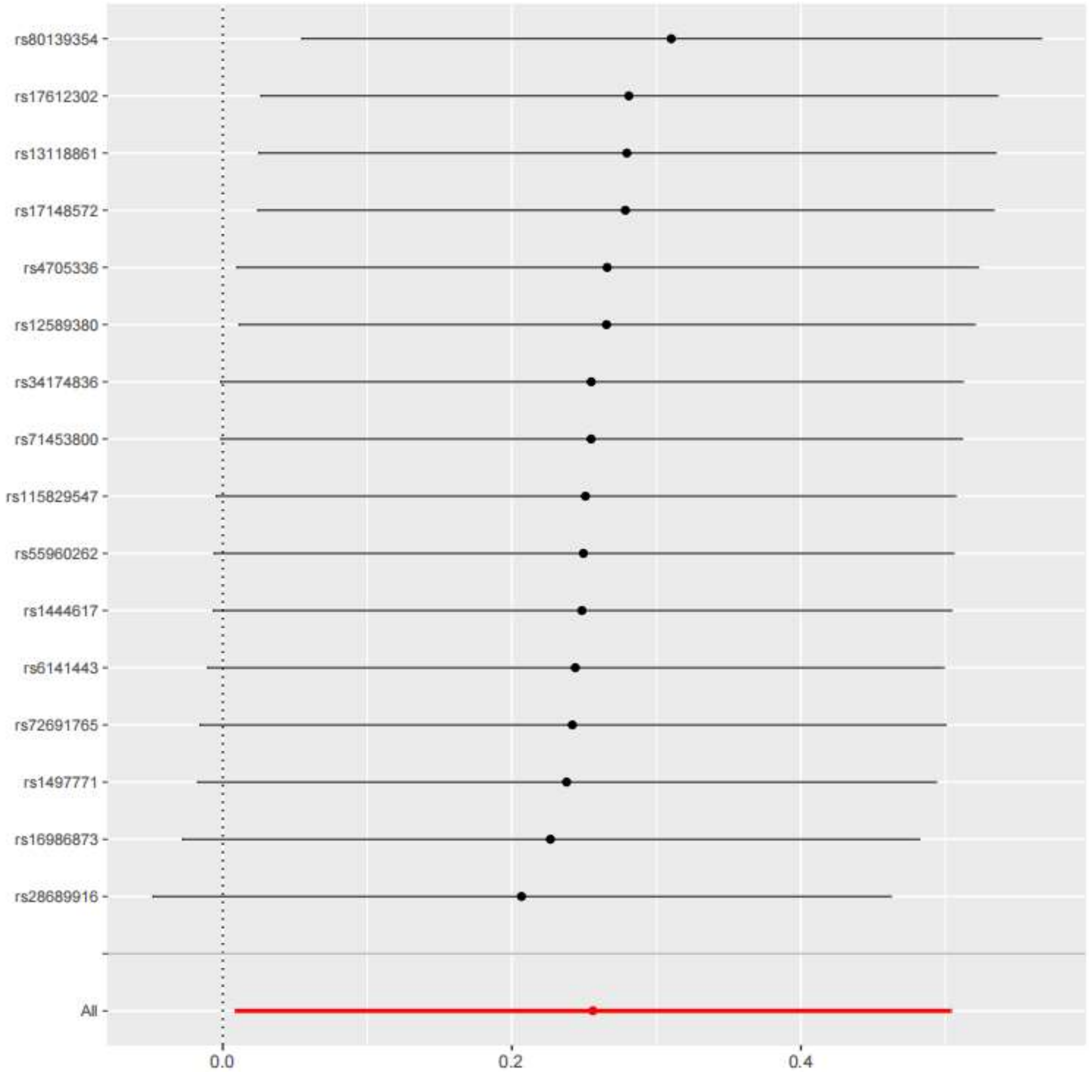

MR leave-one-out sensitivity analysis for UBA6960 abundance in stool on cervical spondylosis

TableS1 gut microbiota accessionId and reported Trait

accessionId	reported Trait
GCST90032270	CAG-273 sp003534295 abundance in stool
GCST90032271	CAG-274 sp000432155 abundance in stool
GCST90032272	CAG-302 abundance in stool
GCST90032273	CAG-345 sp000433315 abundance in stool
GCST90032274	CAG-345 abundance in stool
GCST90032275	CAG-349 abundance in stool
GCST90032276	CAG-390 sp003523225 abundance in stool
GCST90032277	CAG-433 abundance in stool
GCST90032278	CAG-448 sp000433415 abundance in stool
GCST90032279	CAG-448 sp003150135 abundance in stool
GCST90032280	CAG-448 abundance in stool
GCST90032281	CAG-449 abundance in stool
GCST90032282	CAG-452 sp000434035 abundance in stool
GCST90032283	CAG-452 abundance in stool
GCST90032284	CAG-465 sp000433135 abundance in stool
GCST90032285	CAG-475 abundance in stool
GCST90032286	CAG-485 sp002362485 abundance in stool
GCST90032287	CAG-485 sp002404675 abundance in stool
GCST90032288	CAG-488 sp000434055 abundance in stool
GCST90032289	CAG-488 abundance in stool
GCST90032290	CAG-495 abundance in stool
GCST90032291	CAG-510 sp002432425 abundance in stool
GCST90032292	CAG-552 abundance in stool
GCST90032293	CAG-590 sp000431135 abundance in stool
GCST90032294	CAG-632 abundance in stool
GCST90032295	CAG-698 abundance in stool
GCST90032296	CAG-776 sp000438195 abundance in stool
GCST90032297	CAG-776 abundance in stool
GCST90032298	CAG-81 sp000435795 abundance in stool
GCST90032299	CAG-822 sp000432855 abundance in stool
GCST90032300	CAG-822 abundance in stool
GCST90032301	CAG-826 abundance in stool
GCST90032302	CAG-83 sp000435555 abundance in stool
GCST90032303	CAG-83 sp002392625 abundance in stool
GCST90032304	CAG-841 sp002479075 abundance in stool
GCST90032305	CAG-873 sp001701165 abundance in stool
GCST90032306	CAG-877 sp000433455 abundance in stool
GCST90032307	CAG-877 abundance in stool
GCST90032308	CAG-882 sp003486385 abundance in stool
GCST90032309	CAG-884 sp000433875 abundance in stool
GCST90032310	CAG-884 abundance in stool
GCST90032311	CAG-977 abundance in stool

GCST90032312	Caloranaerobacteraceae abundance in stool
GCST90032313	Caloranaerobacter abundance in stool
GCST90032314	Campylobacter D abundance in stool
GCST90032315	Cetobacterium A abundance in stool
GCST90032316	CHKCI006 sp900018345 abundance in stool
GCST90032317	Chloroflexales abundance in stool
GCST90032318	Chromatiales abundance in stool
GCST90032319	Chromobacteriaceae abundance in stool
GCST90032320	Citrobacter A abundance in stool
GCST90032321	Clostridia abundance in stool
GCST90032322	Clostridium E sporosphaeroides abundance in stool
GCST90032323	Clostridium I abundance in stool
GCST90032324	Clostridium M clostridioforme abundance in stool
GCST90032325	Clostridium M sp001304855 abundance in stool
GCST90032326	Clostridium P abundance in stool
GCST90032327	Clostridium saudiense abundance in stool
GCST90032328	Clostridium S felsineum abundance in stool
GCST90032329	Clostridium tertium abundance in stool
GCST90032330	Collinsella abundance in stool
GCST90032331	Comamonas B abundance in stool
GCST90032332	Comamonas abundance in stool
GCST90032333	Coprobacillus cateniformis abundance in stool
GCST90032334	Coprobacillus abundance in stool
GCST90032335	Coprobacter secundus abundance in stool
GCST90032336	Corynebacterium abundance in stool
GCST90032337	Cyanobacteria abundance in stool
GCST90032338	Demequinaceae abundance in stool
GCST90032339	Demequina abundance in stool
GCST90032340	Desulfobacterota A abundance in stool
GCST90032341	Desulfovibrionaceae abundance in stool
GCST90032342	Desulfovibrionales abundance in stool
GCST90032343	Desulfovibrionia abundance in stool
GCST90032344	Desulfovibrio piger abundance in stool
GCST90032470	Massiliomicrobiota abundance in stool
GCST90032471	Megamonas funiformis abundance in stool
GCST90032472	Megamonas abundance in stool
GCST90032473	Megasphaera elsdenii abundance in stool
GCST90032474	Megasphaera sp900066485 abundance in stool
GCST90032475	Megasphaera abundance in stool
GCST90032476	Merdibacter massiliensis abundance in stool
GCST90032477	Methanobacterium B abundance in stool
GCST90032478	Methanobrevibacter B abundance in stool
GCST90032479	Microvirga abundance in stool
GCST90032480	Monoglobaceae abundance in stool

GCST90032481	Monoglobus pectinilyticus abundance in stool
GCST90032482	Monoglobus abundance in stool
GCST90032483	Morganella abundance in stool
GCST90032484	Mycobacteriaceae abundance in stool
GCST90032485	Mycoplasmataceae abundance in stool
GCST90032486	Mycoplasmatales abundance in stool
GCST90032487	Mycoplasmoidaceae abundance in stool
GCST90032488	Negativibacillus massiliensis abundance in stool
GCST90032489	Negativibacillus sp000435195 abundance in stool
GCST90032490	Negativibacillus abundance in stool
GCST90032491	NK4A144 abundance in stool
GCST90032492	Odoribacter laneus abundance in stool
GCST90032493	Olsenella C abundance in stool
GCST90032494	Omnitrophota abundance in stool
GCST90032445	Klebsiella A abundance in stool
GCST90032446	Klebsiella pneumoniae abundance in stool
GCST90032447	Klebsiella abundance in stool
GCST90032448	koll11 abundance in stool
GCST90032449	Lachnoanaerobaculum saburreum abundance in stool
GCST90032450	Lachnospiraceae abundance in stool
GCST90032451	Lachnospirales abundance in stool
GCST90032452	Lachnospira rogosae abundance in stool
GCST90032453	Lachnospira sp000437735 abundance in stool
GCST90032454	Lactobacillus B ruminis abundance in stool
GCST90032455	Lactobacillus B salivarius abundance in stool
GCST90032456	Lactobacillus B abundance in stool
GCST90032457	Lactococcus lactis abundance in stool
GCST90032458	Lawsonibacter sp000492175 abundance in stool
GCST90032459	Lawsonibacter sp002161175 abundance in stool
GCST90032460	Lawsonibacter sp900066645 abundance in stool
GCST90032461	Leclercia abundance in stool
GCST90032462	Lentimicrobiaceae abundance in stool
GCST90032463	Leptospirae abundance in stool
GCST90032464	Leptospirales abundance in stool
GCST90032465	Leuconostoc mesenteroides abundance in stool
GCST90032466	Leuconostoc abundance in stool
GCST90032467	Magnetospirillum A abundance in stool
GCST90032468	Marinilabiliaceae abundance in stool
GCST90032469	Massiliomicrobiota sp002160815 abundance in stool
GCST90032505	Pararhizobium abundance in stool
GCST90032506	Pauljensenia sp000411415 abundance in stool
GCST90032507	Peptococcia abundance in stool
GCST90032508	Phascolarctobacterium sp003150755 abundance in stool
GCST90032509	Phocea massiliensis abundance in stool

GCST90032510	Phocea abundance in stool
GCST90032511	Photobacterium abundance in stool
GCST90032512	Planococcaceae abundance in stool
GCST90032513	Poseidoniaceae abundance in stool
GCST90032514	Prevotella bivia abundance in stool
GCST90032515	Prevotella buccae abundance in stool
GCST90032516	Prevotellamassilia sp000437675 abundance in stool
GCST90032517	Prevotellamassilia abundance in stool
GCST90032495	Paceibacteria abundance in stool
GCST90032496	Paenibacillales abundance in stool
GCST90032497	Paenibacillus J abundance in stool
GCST90032498	Pandoraea abundance in stool
GCST90032499	Parabacteroides johnsonii abundance in stool
GCST90032500	Parabacteroides sp000436495 abundance in stool
GCST90032501	Parabacteroides abundance in stool
GCST90032502	Parachlamydiales abundance in stool
GCST90032503	Paraglaciecola abundance in stool
GCST90032504	Paramuribaculum sp001689565 abundance in stool
GCST90032195	Atopobiaceae abundance in stool
GCST90032196	Aureimonas abundance in stool
GCST90032197	Azorhizobium abundance in stool
GCST90032198	Bacillaceae A abundance in stool
GCST90032199	Bacillales A abundance in stool
GCST90032200	Bacilli A abundance in stool
GCST90032201	Bacillus AY abundance in stool
GCST90032202	Bacillus C abundance in stool
GCST90032203	Bacillus abundance in stool
GCST90032204	Bacillus U abundance in stool
GCST90032205	Bacillus velezensis abundance in stool
GCST90032206	Bacteroides A plebeius A abundance in stool
GCST90032207	Bacteroides A plebeius abundance in stool
GCST90032208	Bacteroides A abundance in stool
GCST90032209	Bacteroides clarus abundance in stool
GCST90032210	Bacteroides eggerthii abundance in stool
GCST90032211	Bacteroides faecis abundance in stool
GCST90032212	Bacteroides intestinalis A abundance in stool
GCST90032213	Bacteroides sp002160055 abundance in stool
GCST90032214	Bacteroides sp003545565 abundance in stool
GCST90032215	Bacteroides stercoris abundance in stool
GCST90032216	Bacteroides thetaiotaomicron abundance in stool
GCST90032217	Barnesiellaceae abundance in stool
GCST90032218	Barnesiella abundance in stool
GCST90032219	Bifidobacteriaceae abundance in stool
GCST90032245	Brevibacillaceae abundance in stool

GCST90032246	Brevibacillales abundance in stool
GCST90032247	Brevibacillus B abundance in stool
GCST90032248	Butyricimonas sp900258545 abundance in stool
GCST90032249	CAG-1000 sp000434555 abundance in stool
GCST90032250	CAG-1000 abundance in stool
GCST90032251	CAG-1031 abundance in stool
GCST90032252	CAG-110 abundance in stool
GCST90032253	CAG-145 sp000435615 abundance in stool
GCST90032254	CAG-145 sp002320005 abundance in stool
GCST90032255	CAG-145 abundance in stool
GCST90032256	CAG-170 sp003516765 abundance in stool
GCST90032257	CAG-177 sp002438685 abundance in stool
GCST90032258	CAG-177 sp002451755 abundance in stool
GCST90032259	CAG-177 sp003514385 abundance in stool
GCST90032260	CAG-177 sp003538135 abundance in stool
GCST90032261	CAG-177 abundance in stool
GCST90032262	CAG-180 sp000432435 abundance in stool
GCST90032263	CAG-194 sp002441865 abundance in stool
GCST90032264	CAG-245 sp000435175 abundance in stool
GCST90032265	CAG-245 abundance in stool
GCST90032266	CAG-269 sp001915995 abundance in stool
GCST90032267	CAG-269 sp001916065 abundance in stool
GCST90032268	CAG-269 sp002372935 abundance in stool
GCST90032269	CAG-273 sp003507395 abundance in stool
GCST90032220	Bifidobacterium adolescentis abundance in stool
GCST90032221	Bifidobacterium angulatum abundance in stool
GCST90032222	Bifidobacterium bifidum abundance in stool
GCST90032223	Bifidobacterium breve abundance in stool
GCST90032224	Bifidobacterium catenulatum abundance in stool
GCST90032225	Bifidobacterium infantis abundance in stool
GCST90032226	Bifidobacterium kashiwanohense abundance in stool
GCST90032227	Bifidobacterium longum abundance in stool
GCST90032228	Bifidobacterium pseudocatenulatum abundance in stool
GCST90032229	Bifidobacterium ruminantium abundance in stool
GCST90032230	Bifidobacterium abundance in stool
GCST90032231	Bin127 abundance in stool
GCST90032232	Blautia A sp000285855 abundance in stool
GCST90032233	Blautia A sp002159835 abundance in stool
GCST90032234	Blautia A sp900066145 abundance in stool
GCST90032235	Blautia A sp900066355 abundance in stool
GCST90032236	Blautia hansenii abundance in stool
GCST90032237	Blautia sp000436935 abundance in stool
GCST90032238	Blautia sp001304935 abundance in stool
GCST90032239	Borreliaceae abundance in stool

GCST90032240	Borreliales abundance in stool
GCST90032241	Brachyspiraceae abundance in stool
GCST90032242	Brachyspirae abundance in stool
GCST90032243	Brachyspirales abundance in stool
GCST90032244	Brachyspira abundance in stool
GCST90032618	UBA6382 abundance in stool
GCST90032619	UBA6398 sp002451695 abundance in stool
GCST90032620	UBA6398 abundance in stool
GCST90032621	UBA644 abundance in stool
GCST90032622	UBA6960 abundance in stool
GCST90032623	UBA7102 sp002315655 abundance in stool
GCST90032624	UBA7177 sp002491225 abundance in stool
GCST90032625	UBA7177 abundance in stool
GCST90032626	UBA7182 sp002491115 abundance in stool
GCST90032627	UBA7182 abundance in stool
GCST90032628	UBA737 sp002451855 abundance in stool
GCST90032629	UBA737 abundance in stool
GCST90032630	UBA7703 abundance in stool
GCST90032631	UBA7748 sp900314535 abundance in stool
GCST90032632	UBA8517 abundance in stool
GCST90032633	UBA8621 abundance in stool
GCST90032634	UBA8904 abundance in stool
GCST90032635	UBA9475 sp002161235 abundance in stool
GCST90032636	UBA9475 sp002161675 abundance in stool
GCST90032637	UBP9 abundance in stool
GCST90032638	UCG-010 sp003150215 abundance in stool
GCST90032639	UNC496MF abundance in stool
GCST90032640	V9D3004 abundance in stool
GCST90032641	Veillonellaceae abundance in stool
GCST90032642	Veillonella rogosae abundance in stool
GCST90032643	Veillonella abundance in stool
GCST90032644	Victivallis sp002998355 abundance in stool
GCST90032172	Absiella dolichum abundance in stool
GCST90032173	Acetobacteraceae abundance in stool
GCST90032174	Acetobacterales abundance in stool
GCST90032175	Achromobacter abundance in stool
GCST90032176	Acidaminococcus fermentans abundance in stool
GCST90032177	Acidaminococcus sp900315205 abundance in stool
GCST90032178	Acidobacteriales abundance in stool
GCST90032179	Actinobacteria abundance in stool
GCST90032180	Actinobacteriota abundance in stool
GCST90032181	Actinomycetales abundance in stool
GCST90032182	Agathobacter sp000434275 abundance in stool
GCST90032183	Akkermansia muciniphila B abundance in stool

GCST90032184	Aliivibrio abundance in stool
GCST90032185	Alistipes shahii abundance in stool
GCST90032186	Alistipes abundance in stool
GCST90032187	Alloprevotella abundance in stool
GCST90032188	Alteromonadaceae abundance in stool
GCST90032189	An181 abundance in stool
GCST90032190	An7 abundance in stool
GCST90032191	Anaeromassilibacillus sp001305115 abundance in stool
GCST90032192	Aneurinibacillaceae abundance in stool
GCST90032193	Aneurinibacillales abundance in stool
GCST90032194	AR31 abundance in stool
GCST90032543	RUG147 sp900315495 abundance in stool
GCST90032550	Ruminococcus D bicirculans abundance in stool
GCST90032551	Ruminococcus D abundance in stool
GCST90032552	Ruminococcus E sp003521625 abundance in stool
GCST90032553	Ruminococcus E sp900100595 abundance in stool
GCST90032554	Ruminococcus E sp900314705 abundance in stool
GCST90032555	Ruminococcus abundance in stool
GCST90032560	Sorangium abundance in stool
GCST90032561	Spirillospora abundance in stool
GCST90032562	Spirochaetia abundance in stool
GCST90032563	Sporomusales abundance in stool
GCST90032544	RUG147 abundance in stool
GCST90032545	RUG420 sp900317985 abundance in stool
GCST90032546	RUG472 sp900319345 abundance in stool
GCST90032547	RUG472 abundance in stool
GCST90032548	Ruminococcus A sp000432335 abundance in stool
GCST90032549	Ruminococcus C sp000437255 abundance in stool
GCST90032556	Saccharofermentanaceae abundance in stool
GCST90032557	Saccharomonospora abundance in stool
GCST90032558	SAR324 abundance in stool
GCST90032559	SM23-33 abundance in stool
GCST90032564	Staphylococcus A fleurettii abundance in stool
GCST90032565	Staphylococcus aureus abundance in stool
GCST90032566	Stappia abundance in stool
GCST90032567	Streptacidiphilus abundance in stool
GCST90032568	Streptococcus sanguinis abundance in stool
GCST90032569	Succiniclasticum abundance in stool
GCST90032570	Succinivibrionaceae abundance in stool
GCST90032571	Succinivibrio abundance in stool
GCST90032572	Syntrophomonadia abundance in stool
GCST90032573	Syntrophorhabdaceae abundance in stool
GCST90032574	Syntrophorhabdia abundance in stool
GCST90032575	Tannerellaceae abundance in stool

GCST90032576	Tepidanaerobacteraceae abundance in stool
GCST90032577	Terrisporobacter othiniensis abundance in stool
GCST90032578	Terrisporobacter abundance in stool
GCST90032579	Thermococcaceae abundance in stool
GCST90032580	Thermococci abundance in stool
GCST90032581	Thermoplasmatota abundance in stool
GCST90032582	Thermoprotei abundance in stool
GCST90032583	Thioalkalivibrionaceae abundance in stool
GCST90032584	TMED109 abundance in stool
GCST90032585	Treponema D abundance in stool
GCST90032586	Treponemataceae abundance in stool
GCST90032587	Turicibacteraceae abundance in stool
GCST90032588	Turicibacter sp001543345 abundance in stool
GCST90032589	Turicibacter abundance in stool
GCST90032590	UBA1033 sp001695555 abundance in stool
GCST90032591	UBA1066 sp900317515 abundance in stool
GCST90032592	UBA1066 abundance in stool
GCST90032593	UBA11471 sp000434215 abundance in stool
GCST90032594	UBA11471 abundance in stool
GCST90032595	UBA1191 abundance in stool
GCST90032596	UBA11963 sp002362595 abundance in stool
GCST90032597	UBA11963 abundance in stool
GCST90032598	UBA1206 sp000433115 abundance in stool
GCST90032599	UBA1375 sp002305795 abundance in stool
GCST90032600	UBA1407 abundance in stool
GCST90032601	UBA1409 abundance in stool
GCST90032602	UBA1417 sp003531055 abundance in stool
GCST90032603	UBA1446 sp002329245 abundance in stool
GCST90032604	UBA1448 sp002329405 abundance in stool
GCST90032605	UBA1448 abundance in stool
GCST90032606	UBA1611 abundance in stool
GCST90032607	UBA1777 sp002320035 abundance in stool
GCST90032608	UBA1777 sp900316255 abundance in stool
GCST90032609	UBA1777 sp900319275 abundance in stool
GCST90032610	UBA1777 sp900319835 abundance in stool
GCST90032611	UBA2658 sp002841545 abundance in stool
GCST90032612	UBA2821 abundance in stool
GCST90032613	UBA2922 sp900313925 abundance in stool
GCST90032614	UBA3282 sp002493835 abundance in stool
GCST90032615	UBA3792 abundance in stool
GCST90032616	UBA3855 sp900316885 abundance in stool
GCST90032617	UBA5394 sp002409725 abundance in stool
GCST90032519	Prevotella sp000436915 abundance in stool
GCST90032520	Prevotella sp002437285 abundance in stool

GCST90032521	Prevotella sp002437565 abundance in stool
GCST90032522	Prevotella sp002933775 abundance in stool
GCST90032523	Prevotella sp900317685 abundance in stool
GCST90032524	Prevotella sp900318625 abundance in stool
GCST90032525	Propionibacterium freudenreichii abundance in stool
GCST90032526	Proteus abundance in stool
GCST90032527	Provencibacterium massiliense abundance in stool
GCST90032528	Provencibacterium abundance in stool
GCST90032529	Providencia abundance in stool
GCST90032530	Pseudomonadales abundance in stool
GCST90032531	Pseudomonas aeruginosa abundance in stool
GCST90032532	Psychroserpens abundance in stool
GCST90032533	QALR01 sp003150035 abundance in stool
GCST90032534	Raoultella abundance in stool
GCST90032535	RC9 sp900317925 abundance in stool
GCST90032536	Rhodanobacter abundance in stool
GCST90032537	Rhodococcus abundance in stool
GCST90032538	Rhodovulum abundance in stool
GCST90032539	Romboutsia ilealis abundance in stool
GCST90032540	Roseibacillus abundance in stool
GCST90032541	Rubneribacter sp002159915 abundance in stool
GCST90032542	RUG013 sp001486445 abundance in stool
GCST90032518	Prevotella sp000434975 abundance in stool
GCST90032370	Escherichia abundance in stool
GCST90032371	Eubacterium callanderi abundance in stool
GCST90032372	Eubacterium F sp000434115 abundance in stool
GCST90032373	Eubacterium I ramulus A abundance in stool
GCST90032374	Eubacterium Q abundance in stool
GCST90032375	Eubacterium R coprostanoligenes abundance in stool
GCST90032376	Eubacterium R sp000431535 abundance in stool
GCST90032377	Ezakiellaceae abundance in stool
GCST90032378	F0428 abundance in stool
GCST90032379	Faecalibacterium prausnitzii E abundance in stool
GCST90032380	Faecalibacterium sp002160895 abundance in stool
GCST90032381	Faecalicatena glycyrrhizinilyticum abundance in stool
GCST90032382	Faecalicatena lactaris abundance in stool
GCST90032383	Faecalicatena sp000364245 abundance in stool
GCST90032384	Faecalicatena sp001517425 abundance in stool
GCST90032385	Faecalicatena sp002161355 abundance in stool
GCST90032386	Faecalicatena sp002397985 abundance in stool
GCST90032387	Faecalicatena torques abundance in stool
GCST90032388	Faecalicoccus pleomorphus abundance in stool
GCST90032389	Faecalicoccus abundance in stool
GCST90032390	Faecalitalea cylindroides abundance in stool

GCST90032391	Fervidobacteriaceae abundance in stool
GCST90032392	Fibrobacteraceae abundance in stool
GCST90032393	Fibrobacterales abundance in stool
GCST90032394	Fibrobacteria abundance in stool
GCST90032345	Dialister sp000434475 abundance in stool
GCST90032346	Dokdonella abundance in stool
GCST90032347	Dorea phocaeense abundance in stool
GCST90032348	Dorea abundance in stool
GCST90032349	DTU024 sp002411105 abundance in stool
GCST90032350	Dysgonomonadaceae abundance in stool
GCST90032351	Eisenbergiella sp900066775 abundance in stool
GCST90032352	Elusimicrobiaceae abundance in stool
GCST90032353	Elusimicrobia abundance in stool
GCST90032354	Elusimicrobiota abundance in stool
GCST90032355	Emergencia abundance in stool
GCST90032356	Endozoicomonadaceae abundance in stool
GCST90032357	Enorma massiliensis abundance in stool
GCST90032358	Ensifer abundance in stool
GCST90032359	Enterobacteriaceae abundance in stool
GCST90032360	Enterococcaceae abundance in stool
GCST90032361	Enterococcus A abundance in stool
GCST90032362	Enterococcus B abundance in stool
GCST90032363	Enterococcus faecalis abundance in stool
GCST90032364	Enterococcus abundance in stool
GCST90032365	Enteroscipio abundance in stool
GCST90032366	ER4 sp002437735 abundance in stool
GCST90032367	Eremiobacterota abundance in stool
GCST90032368	Erysipelatoclostridiaceae abundance in stool
GCST90032369	Escherichia flexneri abundance in stool
GCST90032395	Fimbriimonadia abundance in stool
GCST90032396	Firmicutes A abundance in stool
GCST90032397	Firmicutes E abundance in stool
GCST90032398	Firmicutes I abundance in stool
GCST90032399	Flavobacteriales abundance in stool
GCST90032400	Flavonifractor sp002159265 abundance in stool
GCST90032401	Flavonifractor sp900199495 abundance in stool
GCST90032402	Fournierella massiliensis abundance in stool
GCST90032403	Francisellaceae abundance in stool
GCST90032404	Francisellales abundance in stool
GCST90032405	Fusobacteriaceae abundance in stool
GCST90032406	Fusobacterium A abundance in stool
GCST90032407	GCA-900066135 sp900066135 abundance in stool
GCST90032408	GCA-900066495 sp900066495 abundance in stool
GCST90032409	GCA-900066495 abundance in stool

GCST90032410	GCA-900066575 sp900066385 abundance in stool
GCST90032411	GCA-900066755 sp900066755 abundance in stool
GCST90032412	GCA-900066755 abundance in stool
GCST90032413	GCA-900199385 sp900320755 abundance in stool
GCST90032414	Geminocystis abundance in stool
GCST90032415	Gemmatimonadaceae abundance in stool
GCST90032416	Geobacteraceae abundance in stool
GCST90032417	Geobacter C abundance in stool
GCST90032418	Gillisia abundance in stool
GCST90032419	Gluconobacter abundance in stool
GCST90032420	Gordonibacter pamelaeae abundance in stool
GCST90032421	Gordonibacter abundance in stool
GCST90032422	Gramella abundance in stool
GCST90032423	Haemophilus D sp001679485 abundance in stool
GCST90032424	Halarcobacter abundance in stool
GCST90032425	Halomonadaceae abundance in stool
GCST90032426	Haloplasmatales abundance in stool
GCST90032427	Helicobacter abundance in stool
GCST90032428	Herbidospora abundance in stool
GCST90032429	Herbinix abundance in stool
GCST90032430	Holdemania massiliensis abundance in stool
GCST90032431	Holdemania sp900120005 abundance in stool
GCST90032432	Holdemania abundance in stool
GCST90032433	Hungatella sp900155545 abundance in stool
GCST90032434	Hydrogenophaga abundance in stool
GCST90032435	Hyphomonas abundance in stool
GCST90032436	Intestinimonas massiliensis abundance in stool
GCST90032437	Jiangellaceae abundance in stool
GCST90032438	Johnsonella ignava abundance in stool
GCST90032439	K10 sp001941205 abundance in stool
GCST90032440	K10 abundance in stool
GCST90032441	Kandleria vitulina abundance in stool
GCST90032442	Kineothrix abundance in stool
GCST90032443	KLE1615 sp900066985 abundance in stool
GCST90032444	KLE1615 abundance in stool

TableS2 The forward Mendelian randomization analysis preliminary results

accessionId	MR Egger		Weighted median		Inverse variance weighted		Simple mode		Weighted mode	
	pval	OR	pval	OR	pval	OR	pval	OR	pval	OR
GCST90032172	0.533042799	1.105930065	0.733857203	1.033059617	0.948421774	0.995532615	0.560550785	1.111143187	0.590680103	1.103513942
GCST90032173	0.443416592	2.445306442	0.75179049	1.115080899	0.395675863	1.368909287	0.920508563	0.947333362	0.996301065	0.997619752
GCST90032174	0.135381954	5.693332477	0.795933874	1.091813029	0.797010483	1.075305315	0.760970101	1.194875182	0.77414363	1.176177008
GCST90032175	0.547389794	1.385335801	0.078710598	1.598450412	0.307313173	1.225857535	0.234302714	1.748334564	0.251065669	1.748334564
GCST90032176	0.145039674	1.191833051	0.626283391	0.9604352	0.640468907	0.972745505	0.676845082	0.929446742	0.759116495	0.945937582
GCST90032177	0.891488727	0.9759828	0.208010741	1.144859922	0.185420878	1.112237122	0.2076379	1.311782296	0.187201832	1.306705817
GCST90032178	0.170676743	3.017837494	0.056931263	1.857320668	0.136368313	1.467069016	0.225691125	2.119327939	0.236155034	1.987318379
GCST90032179	0.707905755	1.129344964	0.150727599	0.839140159	0.92823687	0.988672747	0.842646919	1.049274692	0.330656993	0.873506519
GCST90032180	0.745223911	0.842845114	0.428046654	0.864807613	0.824291271	1.03439975	0.423924406	1.36274815	0.363176477	0.804282511
GCST90032181	0.592132617	1.109894729	0.132209481	0.883399918	0.642301029	0.96274338	0.173540104	0.811052871	0.118713909	0.867435396
GCST90032182	0.213850067	0.858974578	0.257577942	1.070742571	0.898784646	1.006766582	0.311300173	1.111577967	0.27059271	1.111577967
GCST90032183	0.609798538	1.032752935	0.802561449	0.990646692	0.431030621	0.975889507	0.715984013	0.973805411	0.76918668	0.979285764
GCST90032184	0.58816841	0.820794413	0.989000687	1.002138355	0.306283726	0.88876447	0.830710111	1.054726193	0.733799099	1.084846996

	6					7				
GCST90032185	0.178756851	0.879384313	0.081798422	0.907979871	0.100544541	0.93011291	0.300837377	0.897073857	0.292664178	0.904886881
GCST90032186	0.689584484	1.038163318	0.659761217	1.024947329	0.975177797	0.998754017	0.593339391	1.054787211	0.752032123	1.030385922
GCST90032187	0.603191346	1.060971991	0.881049345	1.009700331	0.997733155	0.999852396	0.528536616	0.921687709	0.634102367	0.945207141
GCST90032188	0.225339974	0.487116207	0.979663178	0.993020387	0.949009771	0.987085211	0.788130086	1.150725591	0.747160389	1.194389907
GCST90032189	0.980358423	1.007446799	0.251259007	1.14216017	0.070102102	1.217774615	0.987094903	1.003858922	0.829782186	1.05118529
GCST90032190	0.925817912	0.98022967	0.217670906	1.15154139	0.477202697	1.064797738	0.303526594	1.236953209	0.310185965	1.244002116
GCST90032191	0.357652958	1.143186003	0.507023539	1.059610272	0.030111089	1.14575808	0.858209935	1.029554202	0.987249733	1.002255134
GCST90032192	0.241643976	1.552284917	0.969110104	1.007231163	0.630515614	1.067282063	0.489155866	0.794323041	0.645649119	0.854527034
GCST90032193	0.107981092	1.722736439	0.734560729	1.064315907	0.25843702	1.162294003	0.939411781	1.026340527	0.840995967	1.072447632
GCST90032194	0.474527731	1.285045596	0.700324509	0.928367893	0.298303098	0.842034031	0.834468516	0.931656378	0.884216947	0.954266295
GCST90032195	0.263087119	1.271950601	0.924563853	0.988145162	0.834048695	0.979295657	0.981334607	1.006606104	0.997333584	1.000898867
GCST90032196	0.252060747	0.604721121	0.611934218	0.910184496	0.457451834	0.888218163	0.615882949	0.851145606	0.594090308	0.843189165
GCST90032197	0.297424255	0.708613784	0.809432808	0.956759622	0.353020066	0.883695775	0.888014425	1.050187181	0.87585718	1.055701145
GCST90032198	0.0251775	5.109887317	0.482863933	1.275691111	0.209150558	1.414709466	0.813271635	0.859210159	0.827245273	0.870674758
GCST90032	0.308	0.46797	0.9156632	0.9605	0.7767542	1.095	0.50792	0.60368	0.4150192	0.5271

199	007316	3929	01	2015	75	070386	5418	2067	14	54388
GCST90032200	0.8143113	1.326855037	0.150777133	1.98487411	0.976834845	0.987078877	0.207452504	2.691753415	0.214954199	2.759375615
GCST90032201	0.168285878	1.500316276	0.006810992	0.646236901	0.047838494	0.744058638	0.05708364	0.582687689	0.074647723	0.601519468
GCST90032202	0.318878036	1.411615016	0.51126477	0.882726332	0.234418152	0.84445528	0.092820548	0.521993892	0.995222831	1.002097224
GCST90032203	0.520410455	1.320890381	0.639525996	1.128701839	0.160449988	1.308202134	0.604511119	0.780615522	0.632798947	0.787787749
GCST90032204	0.143356881	1.649837703	0.622363065	1.065499891	0.711676776	1.044328066	0.817183603	1.052983447	0.813045334	1.045525469
GCST90032205	0.72261804	1.110537468	0.839076048	1.033027294	0.205777548	1.162058625	0.912973278	0.969173055	0.880725692	0.960690911
GCST90032206	0.74153967	0.966663302	0.112337059	0.922126249	0.004825644	0.901997477	0.383784107	0.930076014	0.379715362	0.933785353
GCST90032207	0.016581375	1.206827303	0.602688371	1.021451924	0.579537906	1.017792753	0.967797894	0.996612332	0.984654075	0.998435895
GCST90032208	0.235397563	1.112143093	0.966925638	1.002162934	0.480672982	0.973111895	0.781232451	1.024263105	0.755602875	1.02840597
GCST90032209	0.322866571	0.890121131	0.799159247	1.015178708	0.423633974	1.037259618	0.952311729	0.993282974	0.920537641	0.989427606
GCST90032210	0.429638295	0.952991268	0.751221096	0.989298474	0.501226624	0.983205404	0.950362605	0.995970779	0.932740645	0.994774857
GCST90032211	0.592070388	0.958017817	0.908073484	0.994601843	0.700094002	1.01367455	0.768122105	0.978472752	0.855484063	0.986824748
GCST90032212	0.664872892	0.963089876	0.044738834	1.09672228	0.062401008	1.06581422	0.156888921	1.119111409	0.192455641	1.124504436
GCST90032213	0.818460766	0.970721078	0.211585676	1.080344877	0.287198097	1.051044226	0.204851365	1.195592055	0.183117357	1.186496664

GCST90032214	0.651164451	1.061526997	0.38574904	0.931322765	0.32873959	0.938435382	0.673161237	0.937336372	0.715032441	0.944315589
GCST90032215	0.283931951	1.121258387	0.790236011	0.986429285	0.395777932	0.967747548	0.975254059	1.002712727	0.951350668	1.005343619
GCST90032216	0.284267523	0.83008283	0.163118149	1.097259997	0.72393789	1.026090879	0.084179879	1.183970781	0.091719118	1.174735558
GCST90032217	0.204241202	1.179465514	0.708988537	1.019425861	0.763428369	1.011646925	0.963818536	1.003641235	0.920523854	1.006969995
GCST90032218	0.140391788	1.138706778	0.828187654	1.009156748	0.954758755	0.998199124	0.830129299	1.014532981	0.827224932	1.014532981
GCST90032219	0.833068909	0.980575272	0.53116978	0.967172573	0.476788216	0.972977051	0.953247842	1.006343887	0.374066055	0.93776834
GCST90032220	0.161167835	0.90744333	0.981302135	0.999263106	0.581822839	0.987698417	0.731100333	1.022063413	0.743287809	1.022063413
GCST90032221	0.370315213	0.927400577	0.057126743	0.911844425	0.178790689	0.950504569	0.817964942	0.980008729	0.185668713	0.904470259
GCST90032222	0.359104719	0.891474463	0.756478933	0.985434359	0.594950419	0.978931387	0.995349816	1.000580309	0.261802458	0.921558408
GCST90032223	0.483234037	0.902370769	0.122915226	0.897878434	0.35268996	0.947527399	0.48357675	0.915406396	0.180868583	0.889525592
GCST90032224	0.445163028	1.161529167	0.618090533	0.972780909	0.919138755	1.006419474	0.202354546	1.203751884	0.381424742	0.928865781
GCST90032225	0.112609701	0.682762675	0.777040993	0.976385849	0.43145255	0.949735459	0.987707387	1.002230856	0.982855694	1.002230856
GCST90032226	0.705346363	0.943100846	0.093265206	0.897103615	0.083827596	0.912133383	0.276434187	0.889081477	0.170993705	0.886914427
GCST90032227	0.219500759	0.871587628	0.504978635	0.961562851	0.84307929	0.991523102	0.587733863	0.947062437	0.389109641	0.926869385
GCST90032228	0.74877286	0.95870346	0.396986074	0.955956971	0.577310859	1.02823279	0.695809647	0.961583953	0.383685891	0.930637259

	4					9				
GCST90032229	0.19118341	0.86160394	0.414891885	0.955501438	0.991168962	0.999572619	0.266608128	1.152570358	0.155154896	0.896355258
GCST90032230	0.803989661	0.970828206	0.7314896	0.981784053	0.905954269	0.994912458	0.309132584	1.119192763	0.466035989	0.952536196
GCST90032231	0.768524211	0.88294286	0.146682578	0.778830775	0.658623953	0.940556075	0.501139557	0.799494088	0.521141974	0.799494088
GCST90032232	0.381546123	0.739587649	0.72680477	0.952567532	0.378856762	0.899109647	0.885678643	1.031273727	0.908738084	1.025577606
GCST90032233	0.826072107	0.94671783	0.521878849	0.932168434	0.980578503	1.002478347	0.798554158	0.944170374	0.801459501	0.949109931
GCST90032234	0.779018531	0.962945882	0.454197902	1.070520902	0.217218933	1.084697335	0.754814382	1.053219114	0.786159145	1.049683267
GCST90032235	0.613207367	1.107299779	0.709108526	0.961623319	0.357422911	0.930961998	0.626352767	0.916618446	0.66824759	0.926987381
GCST90032236	0.621125272	1.06206779	0.32114341	1.084281238	0.492250433	1.040080731	0.446837517	1.11755651	0.471773964	1.100935018
GCST90032237	0.886413218	1.009648613	0.984085951	0.999092662	0.723105882	0.988181375	0.983441965	0.998163245	0.913496213	0.989856505
GCST90032238	0.080420444	1.289474621	0.160145616	1.127698286	0.199072919	1.082348424	0.385536395	1.14078429	0.401703655	1.146776209
GCST90032239	0.272654145	0.714614432	0.531507463	1.111682694	0.608766219	1.079395895	0.622783109	1.138357196	0.681985483	1.115171274
GCST90032240	0.465109572	0.815417404	0.901282226	0.977942603	0.644975728	0.939773481	0.943947432	0.979345452	0.841907219	0.941147042
GCST90032241	0.162215923	0.804325093	0.047052578	0.828592654	0.004399744	0.821674236	0.324507259	0.831170407	0.259265616	0.81305964
GCST90032242	0.135922423	0.771064607	0.015917171	0.761175944	0.002258125	0.785446954	0.217005651	0.76175112	0.194401452	0.76175112
GCST90032	0.126	0.77448	0.0305243	0.7981	0.0019626	0.796	0.30622	0.80706	0.3049880	0.8037

243	417961	2201	56	11692	2	309919	1334	1143	41	17567
GCST90032244	0.13823036	0.809691819	0.023613971	0.811270028	0.003582454	0.830221042	0.226486107	0.815997506	0.226726061	0.812617294
GCST90032245	0.61538489	0.842626684	0.620543173	1.111575247	0.394814157	1.138194479	0.813223248	1.09947023	0.826835832	1.093431938
GCST90032246	0.826504596	1.081170547	0.711808606	1.08524827	0.203130368	1.229478465	0.943998545	0.969899189	0.918424734	0.955263631
GCST90032247	0.83213661	1.039354803	0.724688632	0.968969168	0.508849881	1.044882624	0.732108488	0.94736196	0.74148135	0.95079292
GCST90032248	0.187160275	1.219090939	0.70343733	0.974877404	0.379628514	1.04664433	0.441625946	0.915548246	0.420040293	0.919546877
GCST90032249	0.055150261	0.83474747	0.078973752	0.916991837	0.100982771	0.933801673	0.196073055	0.886064671	0.171620702	0.88385786
GCST90032250	0.096879179	0.852744592	0.676721325	0.979706167	0.980815972	0.998907555	0.869041453	0.985563859	0.857548576	0.985563859
GCST90032251	0.181620384	0.885901769	0.297847866	1.055011396	0.269922007	1.042751542	0.553150476	1.056227436	0.548785265	1.058471607
GCST90032252	0.923867909	0.98971391	0.067890171	0.883995683	0.024771879	0.898314423	0.125360122	0.821538338	0.166728993	0.831020735
GCST90032253	0.45402004	0.936632237	0.847812131	1.010436505	0.638235073	0.981710349	0.710151063	1.037751331	0.734909687	1.031827801
GCST90032254	0.506692211	0.79103556	0.266995192	0.851088666	0.132482335	0.840258788	0.899171115	0.972531316	0.876794107	0.963002346
GCST90032255	0.619839974	0.886038769	0.783184183	0.970103663	0.433504748	0.917622217	0.361570263	1.207269917	0.423738965	1.180505302
GCST90032256	0.133247194	0.816049297	0.935715811	1.004133467	0.814607146	1.008581518	0.825429188	1.023647156	0.845791681	1.019024883
GCST90032257	0.361856789	1.06289509	0.893183556	0.994045263	0.5758131	0.982393851	0.862321939	0.985559511	0.949329515	0.994713618

GCST90032258	0.200265907	1.136073442	0.78884137	0.98546817	0.886675157	1.00625387	0.474497239	0.92455578	0.5214686	0.938749625
GCST90032259	0.337033262	1.054157461	0.857571336	1.006241913	0.776358189	1.00704347	0.807181321	0.984747374	0.842260222	0.98857161
GCST90032260	0.907806001	0.989429499	0.826035161	1.011661895	0.604188922	0.98098446	0.479326788	1.079011036	0.521477336	1.07249802
GCST90032261	0.407852484	1.063026355	0.992795002	1.000390357	0.907650216	1.003667013	0.899774509	0.99067839	0.826741534	0.984672999
GCST90032262	0.204479632	1.145177083	0.166318848	0.95450629	0.834145808	1.006276443	0.388636255	0.948307795	0.335305902	0.948307795
GCST90032263	0.065973496	1.718823249	0.448751312	1.101171369	0.614809031	1.059114804	0.737387414	1.075660524	0.755911143	1.062697462
GCST90032264	0.78690855	0.972706754	0.758077503	1.01515268	0.822610163	0.991560072	0.313912737	1.107643659	0.463458622	1.068319447
GCST90032265	0.549433012	0.944502136	0.450793245	0.963240522	0.472288532	0.971908031	0.4213577	1.079582616	0.759674242	1.02669398
GCST90032266	0.534793705	0.924545574	0.150034594	0.906358755	0.158460017	0.929163952	0.205063025	0.829211975	0.205499361	0.834711051
GCST90032267	0.55498298	1.051730499	0.150386027	0.927005276	0.13418019	0.944870326	0.534780013	0.941422073	0.498629264	0.936421239
GCST90032268	0.198920702	0.764348887	0.472442742	1.067304402	0.55197532	1.038373381	0.469305881	1.115733027	0.463495351	1.115733027
GCST90032269	0.627006502	0.964724356	0.585803702	1.021771892	0.942120237	1.00213786	0.548037376	1.049519362	0.596176897	1.044442667
GCST90032270	0.358029085	1.077641108	0.148069975	1.06526563	0.053676488	1.062686747	0.544605033	1.053971073	0.577432429	1.043174381
GCST90032271	0.242828474	1.087434453	0.517108819	1.024843839	0.366630976	1.025910065	0.43956041	0.943848024	0.4321328	0.943848024
GCST90032272	0.06154578	0.892340009	0.637203508	0.984843318	0.06635077	0.95760637	0.986734511	1.00099804	0.985518735	1.00099804

	1					4				
GCST90032273	0.289844024	0.929575289	0.817828998	0.990071798	0.804444783	0.991828282	0.223016385	1.141803131	0.235545994	1.12809159
GCST90032274	0.508196318	0.958869919	0.536014404	0.975180008	0.556700175	0.981752861	0.211755	1.123225369	0.257072212	1.101527554
GCST90032275	0.002659883	1.221080728	0.587727578	1.020582395	0.331254532	1.03125297	0.087667987	1.164220793	0.092444251	1.148382658
GCST90032276	0.149656871	0.829096875	0.961540715	1.003897749	0.755301701	0.982462317	0.514932037	1.091994189	0.585499524	1.073515869
GCST90032277	0.270019912	0.88787374	0.371800204	0.95143485	0.7195524	0.984832878	0.557957864	0.941675344	0.546164411	0.941675344
GCST90032278	0.804179218	1.046890484	0.760480744	0.973465611	0.722360467	1.02977173	0.582872977	0.916340386	0.629481176	0.933023139
GCST90032279	0.789473604	0.978625859	0.023388124	0.890250898	0.000187226	0.872414301	0.170192246	0.883601681	0.180487971	0.884905702
GCST90032280	0.757470297	1.024261461	0.401289068	0.958375107	0.067089437	0.933824304	0.769591274	0.972993108	0.974973848	0.997241917
GCST90032281	0.654561064	1.050555811	0.732958295	1.024312755	0.608170845	0.974309834	0.685749487	1.057527986	0.692675347	1.054557997
GCST90032282	0.736292408	1.03753887	0.859487205	1.00812037	0.904622989	1.004670004	0.702937198	1.039152281	0.686180602	1.039152281
GCST90032283	0.96056896	1.00509963	0.592045194	1.024463735	0.768846068	1.011665883	0.458661502	1.084024413	0.427520741	1.082123879
GCST90032284	0.117560579	1.34145389	0.964613131	1.003826703	0.10101953	1.132111655	0.727587763	0.939656083	0.770442785	0.951484001
GCST90032285	0.499799277	1.073613207	0.582416029	1.030747057	0.995567411	0.999755056	0.622464417	1.056045644	0.591772875	1.058635583
GCST90032286	0.556053417	1.087691034	0.776552275	0.978413369	0.781519263	1.017164989	0.372359896	0.858447513	0.411409565	0.88145755
GCST90032	0.041	0.65239	0.4727059	0.9313	0.2670791	0.911	0.83543	0.95968	0.7578008	0.9421

287	047915	4547	11	02441	22	856577	9124	6432	4	48433
GCST90032288	0.626287716	1.092901693	0.031288726	1.196741386	0.209032009	1.094291149	0.1040214	1.308459903	0.074452658	1.299160394
GCST90032289	0.50285024	0.918902103	0.307215171	1.074704028	0.799537668	0.986019811	0.520193267	1.107477452	0.515363428	1.098533478
GCST90032290	0.144845532	0.934069788	0.68612145	1.011125845	0.409967279	0.983224292	0.701097068	1.020532079	0.68536443	1.020532079
GCST90032291	0.521968923	1.137431917	0.586234004	1.062997627	0.899321921	0.98960887	0.608832765	1.100030061	0.591035484	1.106071444
GCST90032292	0.577285626	1.05041743	0.671296118	1.019300803	0.259213626	1.038821789	0.924774979	0.991989233	0.886567785	0.988002992
GCST90032293	0.674498949	0.948513056	0.81868056	0.981953645	0.927090078	1.005050547	0.749802078	0.957654158	0.741888648	0.953947194
GCST90032294	0.730656807	0.958351176	0.553917653	1.048569238	0.449512452	0.954385269	0.541459266	1.10681447	0.531252105	1.101525805
GCST90032295	0.951330072	1.001253064	0.764622928	1.00613167	0.70243835	1.007129314	0.425269822	1.089061123	0.675659286	1.008243197
GCST90032296	0.490174081	1.074864011	0.210973823	0.928311681	0.585891265	0.973843294	0.548912903	0.942341755	0.477441612	0.932683193
GCST90032297	0.927781311	1.008056179	0.112508234	0.922164269	0.040067631	0.922638462	0.567307581	0.94841254	0.476762604	0.939795517
GCST90032298	0.338878789	0.924705767	0.048025681	1.095610814	0.215936316	1.042951277	0.269369751	1.104292062	0.199729963	1.102499158
GCST90032299	0.784909872	1.055309262	0.779395176	1.023743387	0.73169012	1.024955089	0.787335197	1.046683569	0.663981938	1.072432395
GCST90032300	0.720244099	0.952387711	0.721636569	0.975747651	0.743552646	0.982848259	0.986800879	0.997588676	0.985756588	0.997588676
GCST90032301	0.562477568	1.088584137	0.938577558	0.993028268	0.909687388	0.992348602	0.978064986	0.995222301	0.946569078	0.989549653

GCST90032302	0.92311831	0.987179254	0.198197695	0.944866613	0.199012679	0.953419648	0.167740385	0.857527123	0.174868649	0.86036637
GCST90032303	0.267676497	1.254817168	0.275014139	1.105854124	0.317328036	1.08332137	0.340776602	1.190287541	0.367032061	1.163465516
GCST90032304	0.987627554	1.003152559	0.144623963	1.19557758	0.666441734	1.041685242	0.28854777	1.26084512	0.228438076	1.266450519
GCST90032305	0.728069987	1.021913638	0.160403526	1.04888463	0.596247566	1.014386107	0.454585172	1.050570696	0.442693392	1.048417134
GCST90032306	0.412358894	0.904110057	0.686064219	0.975370924	0.728898529	0.982841201	0.687977435	0.949791202	0.705621726	0.953080127
GCST90032307	0.366210408	0.910281963	0.597590714	0.97518898	0.816487081	0.991101706	0.784338246	0.972449424	0.737033831	0.967225951
GCST90032308	0.235342705	1.097101913	0.342744343	0.955811105	0.483333714	1.023264841	0.473675712	0.940430438	0.403545359	0.936677373
GCST90032309	0.974836965	0.996505155	0.43187451	1.049344976	0.147187737	1.070749805	0.664650743	1.05169679	0.672235571	1.046405001
GCST90032310	0.85088315	1.018414904	0.891963458	1.00802586	0.412969823	1.037375372	0.698420397	0.961916692	0.671228453	0.957108857
GCST90032311	0.368676328	1.143588912	0.649846067	0.954706928	0.300445771	0.924817179	0.852901408	0.962106842	0.806793606	0.953521502
GCST90032312	0.246499311	0.699638374	0.644826012	0.941959699	0.274036061	0.898535569	0.805816074	0.943682152	0.867555961	0.96140935
GCST90032313	0.341186438	0.733744601	0.400476016	0.904110091	0.381268916	0.917297903	0.6690857	0.905136232	0.746387483	0.928719881
GCST90032314	0.245113404	1.424071856	0.700520827	1.069013035	0.569484844	1.088780562	0.557705395	1.218124126	0.666980712	1.153098248
GCST90032315	0.56439322	0.887808553	0.930721783	0.991213741	0.551642789	0.955878115	0.818562186	1.049960589	0.732336489	1.068424917
GCST90032316	0.75777815	1.042948419	0.137078874	1.121422401	0.114947032	1.09192660	0.338493775	1.139445319	0.370601974	1.123467822

	8					6				
GCST90032317	0.966387466	0.979270769	0.024214534	0.617830516	0.061391211	0.691965949	0.16060625	0.594664571	0.164442922	0.594664571
GCST90032318	0.366611051	0.631389917	0.614321786	1.140018057	0.638925726	1.100575809	0.631241871	1.295122434	0.563424306	1.326935919
GCST90032319	0.020640955	2.265190002	0.334978195	1.207326971	0.105440565	1.27859668	0.649596184	1.221670958	0.703900607	1.187349201
GCST90032320	0.45843501	1.117036369	0.604965108	1.051601593	0.389024457	1.063826902	0.961408971	0.990129842	0.958506725	0.990129842
GCST90032321	0.128737999	1.888446406	0.306289588	1.241220241	0.330196322	1.189541946	0.34362031	1.403806784	0.256396856	1.463603032
GCST90032322	0.065302523	0.629830469	0.43971995	0.900894032	0.331891566	0.901030001	0.626094706	0.86973906	0.667666322	0.890040368
GCST90032323	0.9133462	1.034214584	0.372587223	1.162593485	0.705688371	1.049886497	0.31371716	1.367947341	0.356507596	1.367947341
GCST90032324	0.134446598	1.215971888	0.364915099	1.085610982	0.129723311	1.100782457	0.631150403	1.095791775	0.680102291	1.087571207
GCST90032325	0.162985159	1.388962565	0.75833253	0.962505179	0.335544712	1.102362899	0.613541748	0.904315692	0.521316889	0.887261884
GCST90032326	0.055290282	0.675772325	0.048902006	0.800188369	0.175711837	0.893855507	0.23474378	0.767829227	0.274711904	0.774437111
GCST90032327	0.170718756	0.814659119	0.361488554	1.053441837	0.337613528	1.059199877	0.676605927	1.043726882	0.659410544	1.043726882
GCST90032328	0.48215722	1.257426381	0.396101768	1.168595299	0.196414203	1.219947311	0.680746378	1.119002693	0.670642208	1.117664459
GCST90032329	0.256126936	0.778767956	0.960844566	1.005551687	0.538152966	0.949172484	0.597006012	1.106245067	0.611743399	1.106245067
GCST90032330	0.32494537	0.884439354	0.953722264	0.996856413	0.572528992	1.022246862	0.940622353	0.99290811	0.875735229	0.985444688
GCST90032	0.148	1.50245	0.1807554	1.2617	0.0051240	1.433	0.55283	1.20194	0.6292657	1.1662

331	478877	853	72	07769	21	332328	4519	369	44	93783
GCST90032332	0.342159289	0.858119534	0.507806848	1.069376334	0.822279187	1.016718506	0.288633248	1.255827688	0.304496403	1.262236003
GCST90032333	0.268398639	0.884164639	0.040755664	1.147565387	0.103210319	1.096897052	0.161286281	1.212630961	0.220343145	1.186303667
GCST90032334	0.358150535	0.904232566	0.125440559	1.100790858	0.511182698	1.033686129	0.231165926	1.178812831	0.259181936	1.16796173
GCST90032335	0.619330891	1.183096025	0.615430081	0.943144771	0.935718223	1.010626876	0.195007437	0.782923491	0.243701001	0.785997506
GCST90032336	0.172120288	1.617601606	0.756928126	0.94056092	0.581109839	1.086370861	0.627418231	0.801293949	0.612673427	0.792703728
GCST90032337	0.062277161	0.689097325	0.056417579	0.777823039	0.039212794	0.822560139	0.266320796	0.759216565	0.267288991	0.774758366
GCST90032338	0.620934172	1.224743719	0.431527492	0.861267752	0.126999955	0.806198965	0.265488806	0.664925343	0.87452113	0.945726404
GCST90032339	0.815029849	0.924926289	0.868480475	0.971436933	0.396392501	0.896318548	0.571986459	1.204496568	0.640446557	1.175269167
GCST90032340	0.599778635	1.107306603	0.551794876	1.053803919	0.51096342	1.041270308	0.475364907	1.125715441	0.517888759	1.103689033
GCST90032341	0.894053692	0.98559142	0.176652282	1.076699194	0.400698359	1.033642528	0.308796442	1.124483289	0.315504296	1.108402382
GCST90032342	0.89047218	0.981763345	0.451059788	1.045396495	0.883809275	1.006664775	0.334558172	1.128941683	0.371093822	1.108508796
GCST90032343	0.628654384	1.078023906	0.534082978	1.043527426	0.488022576	1.035138981	0.475043569	1.102524189	0.517431974	1.085756444
GCST90032344	0.738498326	0.977296081	0.910556426	1.004567436	0.868774543	0.994933307	0.832037447	1.018919298	0.878465065	1.012989093
GCST90032345	0.131883129	1.133785768	0.596404397	1.029581732	0.464322679	0.969544513	0.596072859	1.059903105	0.627347544	1.050891321

GCST90032346	0.628041625	0.850173546	0.797224793	0.96218766	0.897565877	1.014742178	0.882598519	0.953234575	0.866919793	1.0538545
GCST90032347	0.413637722	0.831433934	0.295579207	1.117173662	0.575245516	0.955764108	0.381002541	1.188927576	0.330280465	1.19371816
GCST90032348	0.70216887	1.095898905	0.672213192	1.055215257	0.503909508	1.068160731	0.886207701	0.969350949	0.966994435	1.008971449
GCST90032349	0.628521057	0.86171477	0.206770106	0.833008761	0.876049583	0.982012829	0.395601761	0.744359459	0.376293424	0.753463314
GCST90032350	0.316986302	1.244752445	0.140849862	1.197337617	0.133837128	1.148323847	0.35700701	1.251494957	0.347935601	1.251494957
GCST90032351	0.082543134	1.275761808	0.361774829	0.934538166	0.982552213	0.99881139	0.53162304	0.926298053	0.52874446	0.924437484
GCST90032352	0.013240439	1.425494235	0.437049251	1.08061957	0.331490241	1.072147908	0.670151392	1.103402177	0.567516662	1.120427128
GCST90032353	0.544802011	0.864858896	0.244448115	1.212813201	0.212163749	1.147032966	0.415090072	1.339975537	0.33469591	1.32695085
GCST90032354	0.171270829	1.447110814	0.225183569	1.232150972	0.1171513	1.210465636	0.691360505	1.212110759	0.680941182	1.212110759
GCST90032355	0.891195573	1.038015333	0.340164177	0.888480614	0.828523153	0.978075006	0.333915238	0.781480628	0.407084715	0.807376518
GCST90032356	0.344249024	1.297648782	0.395077267	1.169196157	0.474813726	1.097358249	0.696462243	1.134376383	0.732061811	1.112518998
GCST90032357	0.064837475	1.296123874	0.162937046	1.117910596	0.093970075	1.115929706	0.315012398	1.190624092	0.243606027	1.203313375
GCST90032358	0.493575596	0.866147149	0.372625863	0.909599164	0.310657583	0.923327387	0.656113242	0.917551173	0.514126792	0.884112094
GCST90032359	0.173755795	0.837752671	0.55637302	1.036876791	0.955456232	0.997233655	0.595897461	1.058513603	0.55800979	1.061741799
GCST90032360	0.19103449	1.300020604	0.774813994	0.964880139	0.791329458	0.97606575	0.989465198	0.996736867	0.97953742	1.00660402

	6					8				
GCST90032361	0.296447665	1.299809824	0.482229547	0.90612764	0.587069047	1.06584482	0.407319509	0.806484153	0.417593338	0.806484153
GCST90032362	0.615744682	1.075426253	0.068974827	0.829368293	0.087243131	0.881801312	0.234654206	0.77819566	0.262614663	0.785995694
GCST90032363	0.717107829	0.92627991	0.690447502	1.045687988	0.844781222	0.982025158	0.577044531	1.131699428	0.583836484	1.112011345
GCST90032364	0.001752141	2.012017705	0.047862712	1.317466642	0.19549419	1.165802756	0.262008902	1.362384028	0.187449098	1.389993015
GCST90032365	0.420004752	1.099179128	0.239314077	1.078451962	0.595994033	1.025156469	0.537207677	1.087344321	0.502467803	1.098291139
GCST90032366	0.873447632	0.961405051	0.136994199	1.151949083	0.311239578	1.083817143	0.230818032	1.258467833	0.17363767	1.27101754
GCST90032367	0.190038084	1.99815736	0.633997243	1.136409866	0.333108053	1.223262539	0.96600859	0.977614921	0.99574716	1.002859385
GCST90032368	0.849796705	0.97188128	0.663033025	0.963939849	0.47604203	0.957085995	0.60198686	0.923446859	0.667991281	0.943966599
GCST90032369	0.145446761	0.871536705	0.412257278	0.958953291	0.047743178	0.927321577	0.993157651	1.000893203	0.953117548	1.005628351
GCST90032370	0.284184833	0.909602627	0.260251221	0.953022891	0.126468574	0.953339755	0.605691753	0.961170571	0.882057588	1.009996431
GCST90032371	0.479119102	0.854342989	0.470845036	1.090117588	0.665520632	0.962985489	0.467431065	1.164119516	0.453136904	1.167852886
GCST90032372	0.378864254	1.072381282	0.159517715	1.066495076	0.2553405	1.036623075	0.254284954	1.10498984	0.300306362	1.10498984
GCST90032373	0.498339531	0.886001609	0.214105375	1.112453432	0.668682247	1.029925243	0.342628687	1.188538399	0.294848222	1.203828879
GCST90032374	0.119984958	0.565992408	0.509800331	0.885699821	0.695036673	0.935151966	0.357720221	0.741130726	0.448070626	0.781548411
GCST90032	0.524	1.12503	0.5536003	1.0584	0.9864489	1.001	0.31805	1.21090	0.3080428	1.2024

375	129981	9108	38	04736	22	15454	1102	3212	33	3638
GCST90032376	0.171315165	0.891265661	0.977368356	1.001561149	0.344748121	1.037343582	0.739699912	0.965238307	0.788800032	0.971224149
GCST90032377	0.481495622	1.119522871	0.646230406	0.95498432	0.893124438	1.011042017	0.656908405	0.920102147	0.636449926	0.920102147
GCST90032378	0.453312084	1.116717255	0.788751471	0.978591724	0.675065561	1.025428533	0.545884063	0.905274074	0.530942943	0.910484645
GCST90032379	0.166306757	0.759512877	0.705724844	0.966212343	0.237046926	0.918700051	0.835541774	0.965717039	0.8473507	0.968744621
GCST90032380	0.202015507	0.722632799	0.803762952	0.964703702	0.602928679	1.061919082	0.733767097	0.919230703	0.755003003	0.92517782
GCST90032381	0.196521625	0.778620865	0.302545344	0.913298206	0.525212903	0.958992846	0.496422124	0.892582204	0.511135576	0.900086974
GCST90032382	0.359636363	1.107211547	0.874864578	0.991260476	0.404472381	0.966563937	0.877755675	1.015969008	0.865805279	1.017943566
GCST90032383	0.311286915	1.46861879	0.584704824	1.090491026	0.591555483	1.06235066	0.474577418	1.189969279	0.509770025	1.172842144
GCST90032384	0.14648133	1.477819565	0.410482079	1.111176098	0.709193238	1.034508789	0.739018776	1.088139695	0.618226333	1.117918054
GCST90032385	0.553613781	1.216196356	0.562923301	1.076353322	0.264914221	1.13740525	0.634443803	0.883671929	0.661439106	0.88767284
GCST90032386	0.552465803	0.856050779	0.139352198	1.27720327	0.050029461	1.28943284	0.431889048	1.28707235	0.446894499	1.278824828
GCST90032387	0.804724457	1.022434217	0.438494167	1.040921219	0.045110642	1.077216063	0.772232482	0.969381524	0.911976042	0.988454647
GCST90032388	0.631813928	1.05918811	0.794117064	1.02154994	0.906333264	1.006913515	0.761638941	1.043485484	0.832207017	1.027242796
GCST90032389	0.790141594	1.030188637	0.780691745	0.980897436	0.94997931	1.003298644	0.927241832	0.989154834	0.815631475	0.972929191

GCST90032390	0.837376912	1.037124026	0.191396268	1.131490597	0.431227899	1.060668176	0.451103768	1.167654502	0.363201307	1.185591678
GCST90032391	0.863019922	1.057257944	0.729426888	0.950202973	0.722783631	0.962168131	0.876399579	0.962669761	0.760565129	0.92614391
GCST90032392	0.424723191	1.719432485	0.643643261	0.863586065	0.73595276	0.911425272	0.546013825	0.683855633	0.511504899	0.657685157
GCST90032393	0.559681071	1.518805019	0.765742984	1.109665154	0.620174494	1.152969822	0.782599897	1.21522797	0.976292366	1.020137591
GCST90032394	0.240731542	2.420583324	0.170529567	0.615640751	0.772821811	0.916869367	0.259227373	0.463591645	0.278971505	0.485461433
GCST90032395	0.552074617	1.295293919	0.626985258	0.910093168	0.851943712	1.033179762	0.339385275	0.646110829	0.311866746	0.671093449
GCST90032396	0.106101691	3.228925225	0.06218182	1.672621463	0.305994241	1.283334719	0.223427177	1.943755228	0.199480074	1.914921336
GCST90032397	0.461171423	0.727444084	0.64588439	1.127906953	0.719697923	1.069726763	0.427212248	1.487322638	0.428555468	1.487322638
GCST90032398	0.92248559	1.1029229	0.499875454	1.43643626	0.78590837	1.111851485	0.21359738	0.233584633	0.295340726	3.382464477
GCST90032399	0.140501444	0.690536059	0.594317845	0.915842525	0.406880856	0.905743416	0.943340829	1.025260632	0.940194067	1.025260632
GCST90032400	0.682869409	0.883349825	0.946381945	0.991470202	0.979092669	0.997555409	0.994525856	0.998568357	0.94061029	0.984566058
GCST90032401	0.663857301	0.91671253	0.746427718	0.969323088	0.997196199	0.99975952	0.719226859	0.936460133	0.745331456	0.940084278
GCST90032402	0.57478228	0.864722743	0.281702274	0.866061896	0.018307116	0.784179373	0.935051113	1.024407056	0.910431765	1.030349232
GCST90032403	0.99972237	0.999846694	0.838719309	0.961135484	0.347086292	1.139038009	0.806300471	0.925089601	0.753375549	0.903291313
GCST90032404	0.49541320	0.679878199	0.644721409	1.114903366	0.307654297	1.22450006	0.978165802	1.012856513	0.903561062	0.945456272

	9					6				
GCST90032405	0.23874724	0.778450321	0.526889037	1.088252682	0.841397997	0.979061075	0.635503565	1.123372811	0.615158923	1.123372811
GCST90032406	0.745276866	0.940833825	0.098725138	0.830674684	0.059568132	0.856848588	0.125698639	0.713726326	0.110963944	0.721655386
GCST90032407	0.406416729	0.809015619	0.774150966	1.034443972	0.821741974	0.97845695	0.913108424	0.975506244	0.938426135	1.01657508
GCST90032408	0.825919037	0.964540061	0.066787415	1.15901003	0.005255874	1.179684504	0.459824296	1.119803301	0.459533912	1.115799094
GCST90032409	0.627292883	1.096714436	0.413515363	1.063661627	0.414091594	1.050820331	0.4547516	1.109943683	0.465130243	1.098811339
GCST90032410	0.299107762	0.730676776	0.329954959	1.139375657	0.819917043	1.025536818	0.631406293	1.118872627	0.618836698	1.125024357
GCST90032411	0.294456361	1.187953996	0.524908463	1.062672302	0.273067512	1.080901956	0.826595112	1.039048092	0.81173317	1.04499311
GCST90032412	0.605876409	1.068797723	0.528602121	1.050055308	0.48874932	1.03915818	0.808029555	1.038681076	0.786018729	1.041584713
GCST90032413	0.728940383	0.859775841	0.494561609	0.898791981	0.477969854	0.889458277	0.731813355	1.097905898	0.919165017	1.02731308
GCST90032414	0.946157334	0.982840307	0.837661211	0.971484793	0.996968538	0.999618899	0.583209548	0.863167891	0.595807004	0.869327304
GCST90032415	0.535776262	1.262520586	0.628117369	1.088563099	0.432820274	1.098553047	0.180234854	1.647483961	0.143303036	1.647483961
GCST90032416	0.754371791	1.14342408	0.226702668	1.397105296	0.05309504	1.507528709	0.509576486	1.351607545	0.481420344	1.331310537
GCST90032417	0.662691953	1.172690019	0.656736706	0.913706948	0.347145393	0.867281852	0.889942824	0.951101694	0.941753957	0.973580119
GCST90032418	0.251096986	0.553897133	0.746101994	0.953450514	0.368511895	0.911973778	0.952224122	1.017303717	0.873676197	1.044807031
GCST90032	0.159	0.61036	0.7665514	1.0541	0.7725170	1.045	0.96790	0.98370	0.9782723	1.0105

419	186081	4618	05	40069	76	524428	6332	7986	84	21952
GCST90032420	0.647272533	1.067106624	0.844416599	1.013640024	0.622036962	1.030012455	0.982245349	0.996886594	0.771765776	1.036303733
GCST90032421	0.272314417	1.203986655	0.129648311	1.114936753	0.070995838	1.104448132	0.372885115	1.124492233	0.299910649	1.133632908
GCST90032422	0.989158935	1.00339537	0.616800678	1.069359246	0.747522831	1.031925114	0.376182722	1.286143145	0.416351346	1.252550569
GCST90032423	0.645722557	0.941985268	0.5762671	1.036575591	0.9068981	0.993603668	0.371597471	1.122195267	0.368746249	1.122195267
GCST90032424	0.41297695	1.264001737	0.512569853	1.086339474	0.793674272	1.027919399	0.520491048	1.170841112	0.458135176	1.165486622
GCST90032425	0.602855392	1.409713978	0.385669633	1.284913422	0.455142563	1.20538932	0.586127644	1.332685094	0.465935381	1.406640855
GCST90032426	0.357560069	0.90365326	0.203654856	1.08017367	0.233493819	1.060248085	0.325309476	1.121705629	0.286427484	1.125082281
GCST90032427	0.315027497	0.744728614	0.613710743	0.920778541	0.640289879	0.939486541	0.685577555	0.885134073	0.672661667	0.879100709
GCST90032428	0.743733648	1.094014315	0.874065728	1.022871677	0.771721747	1.031433943	0.958940074	1.014468613	0.977266191	1.00727384
GCST90032429	0.336529177	1.269779424	0.977857142	1.003867971	0.874482434	0.983810873	0.621820661	1.138032553	0.619290444	1.133105573
GCST90032430	0.68198847	0.949027155	0.987829056	1.000881597	0.285669132	0.953563032	0.701394421	1.041130775	0.610334565	1.047082167
GCST90032431	0.684500606	0.957003312	0.413819371	0.959219203	0.212797152	0.954629339	0.978675831	0.997287743	0.965148472	1.004495875
GCST90032432	0.99751833	0.99935969	0.876635722	0.988978623	0.517987633	1.04216482	0.993332466	0.998948505	0.925060193	0.988992568
GCST90032433	0.906530639	1.039418265	0.325386983	1.160373477	0.527708224	1.075462126	0.266266898	1.395332921	0.27244023	1.387762148
GCST90032	0.252	1.79508	0.4968000	1.1692	0.9427767	1.012	0.52960	1.28463	0.4608126	1.3018

434	145901	58	63	30201	52	704604	1583	073	1	57476
GCST90032435	0.148395335	2.694132086	0.175604263	1.491333832	0.247711956	1.323431379	0.345516649	1.600822787	0.334220937	1.576195628
GCST90032436	0.91243402	0.974358082	0.059733668	1.23827192	0.023857043	1.200274602	0.229418571	1.281468475	0.265197693	1.265635309
GCST90032437	0.371676439	0.747935041	0.447722603	1.142791667	0.882672257	1.018707497	0.578478045	1.196403336	0.526595257	1.211693089
GCST90032438	0.649367221	1.118148882	0.885010616	0.980082583	0.860486061	1.018285491	0.394256236	1.266145598	0.407750461	1.26047329
GCST90032439	0.356412214	1.156274955	0.027280177	1.195312335	0.162301634	1.103618342	0.162345035	1.232070817	0.107536224	1.235638868
GCST90032440	0.877102772	0.976221968	0.526750226	1.053264882	0.804412634	0.984473188	0.511524381	1.101558426	0.446147957	1.113565212
GCST90032441	0.826237791	0.96834871	0.561648133	0.952059245	0.51359353	0.958424678	0.740098088	0.945715857	0.31166373	0.842139833
GCST90032442	0.467275711	0.78818044	0.660300264	0.930864234	0.620701743	0.944779138	0.668884006	0.864459313	0.706344041	0.889489409
GCST90032443	0.912991459	0.986515689	0.871204699	0.988130181	0.533227298	1.033916891	0.53934784	0.913188715	0.541577095	0.915559268
GCST90032444	0.331092313	0.893212707	0.867134429	0.988111883	0.451989317	1.038946506	0.758808305	0.956101146	0.595581464	0.94305066
GCST90032445	0.205184072	1.108080788	0.938711972	0.995414675	0.500221537	0.973135306	0.159369789	0.829053787	0.948658274	1.008600258
GCST90032446	0.829040105	0.982805139	0.934530845	1.004471549	0.603960427	1.020492394	0.916144674	0.988071973	0.891807611	0.985094532
GCST90032447	0.350417374	0.932797788	0.747170484	1.017112158	0.148461374	1.053147991	0.616599232	0.94494229	0.563360425	0.94494229
GCST90032448	0.18828593	1.877901509	0.26043336	1.422284755	0.045495845	1.574167548	0.547100712	1.402374384	0.646954813	1.284737791

GCST90032449	0.41221122	0.801730665	0.905240388	1.017511323	0.211844117	1.143014915	0.887400055	0.963133259	0.889918902	0.963133259
GCST90032450	0.923155125	0.976104405	0.504072121	1.081533751	0.887421449	1.013244844	0.637219162	1.093838283	0.637628158	1.089045092
GCST90032451	0.563530271	0.858671767	0.295800599	1.139259407	0.223704142	1.120098752	0.533863636	1.142869584	0.551370611	1.142869584
GCST90032452	0.689541983	0.961603773	0.472740904	1.034672405	0.390248298	1.029429612	0.834558149	1.019864191	0.797438409	1.024122773
GCST90032453	0.819175968	1.019694094	0.951709816	0.997022327	0.794109249	0.990954505	0.478983573	1.062016277	0.876297482	0.986863665
GCST90032454	0.168715804	0.876218508	0.966694058	0.997738238	0.232691115	0.943726103	0.724046455	1.040224597	0.692569609	1.043534065
GCST90032455	0.19491135	1.219848919	0.600544771	1.043486322	0.571319465	1.037413444	0.597626429	1.082218469	0.653174576	1.071645968
GCST90032456	0.388783306	0.914938347	0.584630271	1.035277866	0.718814967	0.97955154	0.590540851	1.0783412	0.541134722	1.090648903
GCST90032457	0.685757168	1.071847629	0.532779535	0.943413231	0.646981162	0.964407126	0.784492761	0.947988871	0.649600931	0.930220654
GCST90032458	0.127724511	0.553705024	0.883867005	0.973723363	0.983987126	1.002878594	0.642609175	0.864634641	0.721139432	0.906367961
GCST90032459	0.557751933	1.134044631	0.771587208	0.965412841	0.356477332	1.087108491	0.831738554	0.954908238	0.796022651	0.951054759
GCST90032460	0.39702328	1.137733692	0.126500468	0.878498929	0.097356368	0.902003631	0.254392682	0.820753895	0.317611138	0.855938032
GCST90032461	0.315897552	1.276545125	0.944957144	0.989757182	0.664126614	0.949791587	0.912437713	1.031056605	0.969561424	1.01035192
GCST90032462	0.285336662	0.642752353	0.711178003	1.063325226	0.908283148	1.015691917	0.358314937	1.39993666	0.335339563	0.705067586
GCST90032463	0.19970066	0.555573411	0.613650796	0.884776988	0.154020078	0.77485302	0.916908331	0.951815803	0.915492585	0.951815803

	6					6				
GCST90032464	0.186423957	0.52380468	0.739931555	0.922086574	0.164605383	0.78462433	0.938237497	1.035275916	0.957893075	1.02407034
GCST90032465	0.422151786	1.123161313	0.795195747	1.01891914	0.68195644	1.026461647	0.800609907	1.033170845	0.793767396	1.033170845
GCST90032466	0.209099113	1.173637781	0.583442103	1.039167644	0.561942693	1.033356524	0.619864118	1.063949295	0.567287907	1.063949295
GCST90032467	0.407310784	1.571987605	0.021278837	1.689250609	0.105818878	1.351102777	0.082518357	1.976862458	0.106587979	1.954792182
GCST90032468	0.844569713	1.075758309	0.423058495	0.862294665	0.636075725	0.934905376	0.554966421	0.802896644	0.531724503	0.802896644
GCST90032469	0.936297996	1.009291483	0.11260992	1.09833169	0.399189945	1.036773814	0.172306028	1.194563169	0.176652942	1.171746752
GCST90032470	0.716833342	0.9446028	0.223699942	1.081157884	0.376914215	1.047851039	0.361684484	1.100851048	0.392445643	1.095187809
GCST90032471	0.193185672	1.092276712	0.296208742	1.055147021	0.774407722	1.010431727	0.20496526	1.161598857	0.217571367	1.145235031
GCST90032472	0.172563732	1.086483466	0.170433894	1.064877075	0.225858127	1.040144379	0.379133685	1.097358415	0.324864848	1.112163703
GCST90032473	0.154334797	0.898331703	0.409600454	0.958489621	0.857845401	1.006511777	0.365411385	0.916443003	0.4280972	0.925892677
GCST90032474	0.198443898	0.800264299	0.871840575	0.981160853	0.95961914	1.004653	0.633140795	0.882659127	0.608712804	0.891541502
GCST90032475	0.454023427	0.939013405	0.211448779	1.063261666	0.761703141	1.012468663	0.31411752	1.125367925	0.38463936	1.107482523
GCST90032476	0.979298799	0.994250878	0.380389875	1.087549688	0.761241044	1.023093456	0.39852454	1.15583726	0.425008704	1.153207077
GCST90032477	0.051075738	1.618138276	0.122992901	1.216004892	0.020504068	1.247030116	0.30678993	1.250864779	0.311452164	1.239711491
GCST90032	0.847	0.94733	0.8089263	1.0332	0.8914960	1.013	0.94049	0.98296	0.9426874	0.9829

478	312106	8652	14	89488	32	652757	0661	9973	69	69973
GCST90032479	0.640982922	0.849956627	0.67592204	1.091840367	0.066423255	1.336827138	0.871003084	0.941448774	0.87525357	0.946690106
GCST90032480	0.52572574	1.147822999	0.913534805	1.008474242	0.634101313	0.961865826	0.928981248	0.98380654	0.994341745	1.001044437
GCST90032481	0.288422652	1.179928482	0.830237289	0.984544409	0.934479472	0.995000982	0.797147282	0.962614617	0.839821277	0.973861653
GCST90032482	0.571910494	1.089772591	0.755044257	0.979610118	0.888844668	0.991196432	0.576149115	0.922712058	0.691625891	0.950580505
GCST90032483	0.393925917	1.099533862	0.953930891	0.995318297	0.449214895	1.045869768	0.906343447	0.978840939	0.95577991	0.990250824
GCST90032484	0.102605206	2.178007706	0.252136119	1.315735469	0.722550503	1.073071725	0.218033228	1.743769053	0.199913519	1.763452139
GCST90032485	0.033631342	0.550396022	0.107385602	0.781220748	0.152176777	0.8541664	0.15405101	0.655871366	0.157332322	0.662582061
GCST90032486	0.675617284	0.796857083	0.944586664	0.985466958	0.385529321	1.176787662	0.515509458	0.796453901	0.574268596	0.818583262
GCST90032487	0.279498959	0.799920729	0.11841248	0.823423581	0.333066097	0.91608895	0.499710042	0.84474906	0.487523055	0.850310898
GCST90032488	0.993379188	1.001579999	0.953727613	1.006294088	0.990384164	0.999095479	0.997589977	0.999427173	0.954587002	1.011653004
GCST90032489	0.356061821	1.0990227	0.153845682	1.077007636	0.449493516	1.031996234	0.274656475	1.153422553	0.270310868	1.159071681
GCST90032490	0.271153201	0.818498704	0.769631186	0.9754223	0.664587058	0.97387498	0.8886243	0.977724289	0.97791295	1.00464475
GCST90032491	0.25643923	1.523623891	0.04345035	1.437257528	0.04040974	1.313132702	0.083626243	1.781868344	0.097111746	1.752689814
GCST90032492	0.80259887	1.023405632	0.55544417	0.964539111	0.458930243	0.967513976	0.725210788	0.962028297	0.7225585	0.963861398

GCST90032493	0.547532162	1.119909099	0.355058102	1.107531519	0.426417747	1.067731315	0.443093386	1.154408484	0.404647691	1.169316386
GCST90032494	0.026434996	4.127886118	0.426396219	1.346682203	0.473870899	1.219280081	0.440446347	1.751475776	0.483882385	1.618438788
GCST90032495	0.431440671	1.706361516	0.599107107	0.819942824	0.954291801	1.017131426	0.603813759	0.691576538	0.61154023	0.702117495
GCST90032496	0.247148212	3.784440012	0.472587856	0.685659173	0.869740364	0.929549734	0.350941737	0.41767274	0.357361226	0.426345876
GCST90032497	0.539031329	1.252345587	0.703796401	1.063827829	0.571188048	1.071588141	0.370312696	1.320002535	0.455635045	1.252516355
GCST90032498	0.787000849	1.105242104	0.532868632	0.890187702	0.667751685	1.058541432	0.605028383	0.823892956	0.603753333	0.823892956
GCST90032499	0.447509944	0.901685605	0.644879489	0.971587802	0.60505689	0.974429294	0.610844399	0.935424957	0.806406695	0.968931468
GCST90032500	0.27537376	1.061668195	0.946716855	1.002645857	0.865433983	1.004751808	0.912185043	1.007877905	0.890637047	1.009288414
GCST90032501	0.547958268	0.914092011	0.306037362	0.946311645	0.332983416	0.954334322	0.409579075	0.91504936	0.437209867	0.92092243
GCST90032502	0.740111613	1.193937745	0.865476953	1.040530689	0.979440526	1.004765699	0.900921547	0.952277976	0.983691814	0.992694755
GCST90032503	0.413174818	0.651837743	0.832420864	0.964956436	0.877784349	1.025455032	0.506227149	0.803569222	0.52883747	0.831967772
GCST90032504	0.500955607	0.880662853	0.732933426	0.970340683	0.653718235	0.966189504	0.736921487	0.943459951	0.936695868	1.013149296
GCST90032505	0.010439123	0.386804505	0.445148552	1.168740188	0.890496291	0.975407497	0.369087309	1.446025013	0.407276782	1.397788264
GCST90032506	0.153633776	1.194828038	0.141605356	1.112029947	0.227716503	1.066724526	0.400234942	1.12507129	0.380837395	1.12507129
GCST90032507	0.90477398	1.036451278	0.378267053	1.16104168	0.299542094	1.15959794	0.66494645	1.14439584	0.804997975	0.929429533

	8									
GCST90032508	0.091221213	0.762080261	0.872683251	1.012347337	0.940509075	1.005430909	0.409888397	1.13894622	0.622096187	1.072140253
GCST90032509	0.014441698	0.600626717	0.994787538	0.99931652	0.63021458	1.04357137	0.89048906	0.975828582	0.954964082	0.989653431
GCST90032510	0.367282244	0.849202779	0.895989338	0.987731871	0.200142327	1.114799549	0.672573892	0.935492632	0.744894228	0.946904146
GCST90032511	0.82713839	0.913295698	0.253094333	1.27038394	0.062431503	1.343712098	0.973193201	1.013247636	0.908237839	1.040953154
GCST90032512	0.758996125	0.790101342	0.898527153	0.956163937	0.971488256	0.989127572	0.312750289	0.46633772	0.324965596	0.490616989
GCST90032513	0.459476151	1.296985186	0.284874534	1.259583574	0.041808789	1.367192061	0.538117163	1.258282202	0.519693796	1.258282202
GCST90032514	0.204900667	0.857161753	0.65002221	0.966181578	0.924262204	0.994866688	0.780507523	0.963371124	0.796483814	0.965607429
GCST90032515	0.371809278	0.903558142	0.081615599	0.895840567	0.607804939	0.976128078	0.282537766	0.869217185	0.277723364	0.873547261
GCST90032516	0.566990107	1.098247527	0.285791981	1.074331045	0.585773969	1.031212642	0.427997645	1.130017161	0.372820304	1.130017161
GCST90032517	0.199878063	0.89116336	0.554812857	0.970312472	0.642402202	1.017067105	0.570004561	0.94776895	0.576864351	0.94776895
GCST90032518	0.157159686	1.108942373	0.679288359	1.017826032	0.674734262	1.014378454	0.836189708	1.015768049	0.768801115	1.022101738
GCST90032519	0.873875694	1.016842714	0.933447544	0.995860018	0.838135721	1.00834658	0.900961721	0.989070898	0.904896921	0.989070898
GCST90032520	0.942241719	1.013036842	0.161190621	0.910910499	0.116722606	0.909596834	0.366043889	0.901220045	0.355247617	0.904087834
GCST90032521	0.328527425	1.095234553	0.588387749	0.970835359	0.994577345	1.000265295	0.665711244	0.954144478	0.667674219	0.954144478
GCST90032	0.537	0.91714	0.9503889	1.0050	0.7362961	0.980	0.55214	1.09689	0.6047701	1.0843

522	055928	2689	37	00654	16	090319	7934	8898	37	18124
GCST90032523	0.319603993	1.114296496	0.75089859	1.01985151	0.116781935	1.076218769	0.978539344	1.003024784	0.948153378	1.007264374
GCST90032524	0.309101647	0.801370109	0.188942686	0.885847206	0.039247321	0.857470864	0.423044776	0.888013543	0.40291084	0.885093191
GCST90032525	0.641930804	1.087859109	0.306152798	1.072582892	0.306373266	1.063383061	0.347889599	1.130419316	0.335644657	1.113994436
GCST90032526	0.380800301	1.163341169	0.977358725	1.002910095	0.699854099	1.031851531	0.790176264	0.949307606	0.809016737	0.954680814
GCST90032527	0.484819873	1.246954893	0.429914977	1.086113308	0.255329648	1.101112698	0.395203002	1.179131487	0.829981032	1.044494813
GCST90032528	0.709366186	1.090825495	0.405603159	1.077151412	0.186887833	1.096196454	0.868077897	1.03103725	0.920604878	1.017478563
GCST90032529	0.467324694	1.165256788	0.87396318	1.019820751	0.962003623	1.004274073	0.630386465	1.139409114	0.625526786	1.139409114
GCST90032530	0.764402247	0.869511524	0.692725922	0.879802889	0.585344726	0.880779739	0.905906133	1.075982869	0.969462227	0.976688541
GCST90032531	0.566814658	1.160008375	0.203894796	1.196742207	0.470050144	1.097052988	0.322735944	1.272589261	0.301318315	1.263062051
GCST90032532	0.7644849	0.911872251	0.843650949	0.974756013	0.634343594	1.060001349	0.808489264	0.949233561	0.818511107	0.955292018
GCST90032533	0.099997235	0.538399338	0.469452584	0.910090467	0.215981904	0.888265898	0.513802084	0.874537358	0.584712254	0.901067143
GCST90032534	0.315453005	1.126092977	0.396413382	1.077538793	0.165118654	1.095426167	0.711587454	1.074238683	0.552668996	1.109810621
GCST90032535	0.199084255	0.535480166	0.566936016	1.097175576	0.48504395	1.102287771	0.223432251	1.547177847	0.229924345	1.524578185
GCST90032536	0.139840014	2.091651016	0.279324664	1.23393011	0.110908671	1.341358576	0.565125264	1.200646998	0.670817083	1.145655095

GCST90032537	0.785305429	1.075847801	0.614222408	0.911914691	0.890116462	1.018450932	0.523519138	0.798665111	0.529591361	0.817721159
GCST90032538	0.334508857	0.656826549	0.407776838	0.854390346	0.632748252	0.934655043	0.301842631	0.706441734	0.328893478	0.713279578
GCST90032539	0.088680555	1.324214737	0.125803235	1.137823779	0.132401493	1.094980568	0.216131666	1.225331919	0.209768355	1.232957639
GCST90032540	0.592653841	1.301426372	0.680979834	1.107161812	0.812423293	1.049838658	0.616733834	1.194320385	0.643463436	1.174007884
GCST90032541	0.196423271	1.258477829	0.52743428	1.066977874	0.581357626	1.042219355	0.643151304	1.08268565	0.582274409	1.096478666
GCST90032542	0.61683961	1.106086205	0.36335548	0.901220704	0.636379511	0.958190049	0.440833344	0.827111251	0.418684505	0.831582916
GCST90032543	0.826616256	1.154798875	0.921795718	0.977119647	0.775459137	0.942248241	0.84400061	0.928840363	0.867408497	0.935987436
GCST90032544	0.400999358	0.638903506	0.753310747	1.076213654	0.520226562	1.122311542	0.837246775	1.08206569	0.853461707	1.069869229
GCST90032545	0.020467356	0.422150233	0.067263091	0.734491235	0.022578721	0.721004311	0.325714138	0.742142795	0.343695991	0.74695354
GCST90032546	0.858720445	0.956161556	0.186552738	1.189970274	0.952907773	0.993885169	0.421074379	1.221162242	0.354712483	1.234246192
GCST90032547	0.763576206	0.937786769	0.731749805	0.961202075	0.782761194	0.976451257	0.540095172	1.163559353	0.43595006	1.193230132
GCST90032548	0.51054171	0.87837526	0.358751973	0.913546683	0.04760553	0.862703931	0.841168153	0.963027265	0.815989513	0.958990341
GCST90032549	0.312852496	1.08824088	0.593702091	1.026955929	0.684471984	0.985841938	0.297024267	1.106690347	0.327765259	1.100828302
GCST90032550	0.7735783	1.035334355	0.243980763	1.056490466	0.446788549	1.029664874	0.16162114	1.15592162	0.153882008	1.153365796
GCST90032551	0.57720926	1.074684012	0.051120398	1.09898054	0.28393014	1.0431211	0.118146042	1.182202288	0.088065485	1.177422724

	9									
GCST90032552	0.04155614	1.339840877	0.437783183	0.930302693	0.35593359	0.928444953	0.621462298	0.920054401	0.559911988	0.916008756
GCST90032553	0.751185244	1.099949293	0.406316753	0.889857868	0.263469077	0.886604879	0.705251065	0.905326939	0.702516772	0.908672336
GCST90032554	0.183372174	0.907526785	0.837788786	0.990015081	0.384423856	1.032248961	0.641357807	0.94978045	0.527628286	0.941524006
GCST90032555	0.972948611	1.007892535	0.479966719	1.106622348	0.216336199	1.13746568	0.595130194	1.159268118	0.467019263	1.235382492
GCST90032556	0.468881162	0.706647345	0.772642512	1.077803368	0.960767628	1.009876266	0.752800403	1.166243231	0.649671348	1.22433193
GCST90032557	0.456097335	1.242326452	0.054163368	1.307926573	0.002237636	1.348535881	0.236754472	1.452005336	0.272908307	1.378382064
GCST90032558	0.265835619	1.758047375	0.147196392	1.462039554	0.113776157	1.389148536	0.114440853	2.381692976	0.131771205	2.268840045
GCST90032559	0.359138383	1.491485608	0.071765876	0.746609009	0.435930841	0.884491835	0.181684386	0.642036321	0.198890881	0.642036321
GCST90032560	0.176433726	0.686569296	0.480833293	1.114810133	0.538441604	1.083407662	0.432047145	1.32907177	0.44362984	1.289235257
GCST90032561	0.968337413	1.016664415	0.741695223	1.066620599	0.64167274	0.930451213	0.381957056	1.453134638	0.32048985	1.453134638
GCST90032562	0.750114447	1.059444567	0.524734438	1.08727817	0.873444016	1.015121959	0.837349249	1.065366657	0.382176585	0.755166856
GCST90032563	0.964204408	1.01853939	0.669327231	1.118397258	0.86128181	1.033696016	0.283310302	1.761069627	0.254225599	1.761069627
GCST90032564	0.639827703	0.950765882	0.263189001	0.93779346	0.137807749	0.939322022	0.535868273	0.938669743	0.571085301	0.943979871
GCST90032565	0.550938889	1.15261926	0.698416754	1.051142074	0.337044209	1.089602043	0.918178869	1.024477478	0.968064365	1.008692434
GCST90032	0.682	0.90308	0.6221304	0.9153	0.9634961	1.005	0.59736	0.84425	0.6019883	0.8387

566	05976	9283	87	46341	98	655547	4197	7281	82	93018
GCST90032567	0.347637227	1.792909058	0.25009403	1.274784513	0.080091492	1.339548226	0.419561639	1.353076779	0.732045218	0.887143499
GCST90032568	0.71960761	1.058544649	0.513651363	0.938793335	0.775447018	1.020826516	0.449626866	0.87783071	0.443511993	0.880347057
GCST90032569	0.725962278	0.957200998	0.806445537	0.981788755	0.832514293	0.987250904	0.647945025	0.920150038	0.712088584	0.93587347
GCST90032570	0.512008136	0.945338852	0.288594737	0.940102007	0.20891936	0.949576898	0.870168781	0.975983295	0.804911272	0.969269249
GCST90032571	0.334512069	1.089648068	0.521927476	1.034085266	0.145509381	1.056153811	0.313527155	1.133772558	0.591250671	1.068721579
GCST90032572	0.95571234	1.026350627	0.07779096	0.591100008	0.608889989	0.896490648	0.246134047	0.488358519	0.238536795	0.498097146
GCST90032573	0.440127523	1.221083508	0.804735571	0.96252361	0.937888413	1.008855603	0.583474419	0.854027304	0.56004232	0.844726796
GCST90032574	0.945363621	1.040596741	0.486180255	0.846574145	0.880367367	1.031839988	0.712744288	0.865429319	0.628718855	0.832733755
GCST90032575	0.481247072	1.114406254	0.358802246	0.946328586	0.962093574	1.002482758	0.496717506	0.923138214	0.475415254	0.920020244
GCST90032576	0.82248123	0.922801455	0.694298804	1.067822337	0.053670686	1.385122341	0.7132438	0.85036855	0.687813812	0.894910803
GCST90032577	0.42909738	0.886929472	0.504782561	0.940524448	0.234421665	0.923626783	0.606823383	0.916228791	0.612684629	0.922795547
GCST90032578	0.665647349	1.059854157	0.837788201	0.984167075	0.377035428	0.952879596	0.97449164	1.004594188	0.94369636	1.009797567
GCST90032579	0.658212063	0.883595981	0.629231025	0.927710289	0.88352282	1.018157734	0.310674576	0.704879836	0.297825598	0.716000317
GCST90032580	0.999861458	1.000061326	0.200189777	1.262760544	0.208776876	1.183571072	0.308815486	1.393254444	0.318736965	1.376000667

GCST90032581	0.776917509	1.074561038	0.732622473	0.944964132	0.86775529	0.977849618	0.472768835	0.741842779	0.608115594	0.816502384
GCST90032582	0.775366625	0.836108755	0.127028504	0.626459042	0.286846138	0.758579596	0.359812223	0.594367341	0.283637186	0.601618183
GCST90032583	0.418040164	1.294575475	0.968853652	1.008059534	0.445961152	1.1317062	0.795379083	0.913213137	0.762227024	0.903311921
GCST90032584	0.841805513	1.060185573	0.080706822	1.266378968	0.008892125	1.288802837	0.573679241	1.177221752	0.76789817	1.083903222
GCST90032585	0.366582738	0.903818803	0.062162071	0.868748256	0.177969186	0.934732917	0.24940475	0.795150571	0.226284382	0.795150571
GCST90032586	0.829823279	1.027818006	0.297857216	0.915788962	0.123773741	0.911435929	0.336884659	0.813900654	0.466052305	0.857917869
GCST90032587	0.379841364	0.916345659	0.095551436	1.099746894	0.132032239	1.065138261	0.257040097	1.119957071	0.26528636	1.124323012
GCST90032588	0.641424303	1.046876016	0.175027074	1.074569728	0.422207427	1.03165809	0.258788611	1.114758608	0.2233874	1.122399111
GCST90032589	0.433658514	0.920525742	0.314400246	1.053455973	0.158849644	1.056612572	0.561737447	1.053277445	0.498513845	1.058159833
GCST90032590	0.348690256	1.305596914	0.96563557	0.993364617	0.80557422	1.030176824	0.881489656	0.957915679	0.871442832	0.957915679
GCST90032591	0.026191311	2.407114166	0.698312902	1.083178019	0.715293839	0.940843359	0.477115445	1.28808062	0.666106755	1.147647337
GCST90032592	0.951398902	0.977163023	0.321371955	0.836823717	0.493597882	0.903773835	0.501850468	0.811145494	0.524398694	0.818510597
GCST90032593	0.526305947	1.082638007	0.414686466	1.055843019	0.079600917	1.09161397	0.892542728	1.016638549	0.944235342	0.992093267
GCST90032594	0.635705954	1.049219017	0.452281712	1.041024961	0.092309939	1.073413652	0.986104099	0.998271658	0.84986173	0.981934072
GCST90032595	0.17498789	1.262240703	0.636511093	1.053743833	0.430219424	1.06560752	0.893742079	0.976697106	0.926602042	0.984266334

	4					6				
GCST90032596	0.284642661	0.821477324	0.843049545	1.020497114	0.561374774	0.958385263	0.755918134	1.05755949	0.747063523	1.05755949
GCST90032597	0.406243478	0.865255054	0.868714233	0.982836648	0.575426837	0.958133896	0.844226982	0.966514339	0.869915332	0.972580296
GCST90032598	0.602902489	0.918976527	0.327954597	0.944024221	0.268576085	0.932883749	0.269791118	0.881705217	0.386019054	0.91190594
GCST90032599	0.596280307	1.099306943	0.730074714	1.039420731	0.536155254	0.945485017	0.402739748	1.25099244	0.395495513	1.221950574
GCST90032600	0.774610899	1.150737371	0.730195697	1.08411495	0.200846343	1.323327618	0.971886327	0.987614425	0.924429336	1.032840377
GCST90032601	0.307353236	1.157768941	0.582032194	1.046425462	0.592661372	1.032321139	0.8600094	1.027903863	0.850612782	1.027903863
GCST90032602	0.530141354	0.918175523	0.213438483	1.096774106	0.180272182	1.080182987	0.438596704	1.124506871	0.438773506	1.121060318
GCST90032603	0.901394359	0.96987921	0.942541304	1.009965419	0.89034458	0.985999825	0.964031427	1.011211966	0.847410357	1.048763712
GCST90032604	0.512308079	1.219276443	0.239342023	0.843457391	0.921946493	0.98928969	0.300905139	0.761059013	0.31753553	0.764011134
GCST90032605	0.196942349	0.611939339	0.017826493	0.726440622	0.002761829	0.729175397	0.129071721	0.711969928	0.131977298	0.711969928
GCST90032606	0.650760362	1.203087305	0.959765925	1.009083039	0.986546926	1.002239772	0.907980557	1.036043045	0.92964881	1.026666933
GCST90032607	0.648633064	0.914272236	0.258843819	1.156199071	0.318610501	1.096930382	0.246951713	1.340578595	0.262674213	1.345893737
GCST90032608	0.630238369	1.128650011	0.45135818	1.111266184	0.511459129	1.07296541	0.514158027	1.158822215	0.590411445	1.14502905
GCST90032609	0.715245547	1.179777732	0.376951687	1.194791827	0.668686294	1.088443441	0.42996563	0.714388403	0.739752125	1.116231965
GCST90032	0.241	1.37366	0.5844192	0.9207	0.6092356	0.949	0.57135	0.86152	0.5848904	0.8615

610	718122	1352	33	52495	97	775898	0168	4609	75	24609
GCST90032611	0.963890979	1.016237704	0.577926247	0.899730954	0.53412901	0.919237656	0.326978362	0.720861675	0.355632902	0.750436374
GCST90032612	0.767444128	1.036687071	0.70701631	0.974962135	0.374997998	0.959136055	0.885543305	0.979087485	0.879776111	0.979087485
GCST90032613	0.446162725	1.283945935	0.392491626	1.121777106	0.326176638	1.111136857	0.611362331	1.149428514	0.592332446	1.149428514
GCST90032614	0.414659128	1.284525771	0.468927657	0.892954052	0.960027354	0.994232371	0.37408858	0.795623536	0.31468637	0.781602805
GCST90032615	0.679083733	1.099959047	0.911383446	0.986707469	0.886672758	1.012455804	0.57294321	0.87546519	0.529756503	0.867354827
GCST90032616	0.034454512	0.507937171	0.223792114	0.84229753	0.290729416	0.905980097	0.573645629	1.156991385	0.154199748	0.741532785
GCST90032617	0.144566098	0.818369678	0.204241667	0.901429859	0.466136202	0.955884706	0.399209863	0.867220563	0.365671488	0.863658295
GCST90032618	0.208229941	0.798221745	0.144796329	0.865892041	0.063634914	0.874714507	0.327089889	0.843452657	0.391441797	0.850393805
GCST90032619	0.930304667	0.988841115	0.39152564	0.937884427	0.827387929	0.988330004	0.425644324	0.90287928	0.513292122	0.915818736
GCST90032620	0.989467061	0.99871159	0.241309554	0.940601376	0.128299051	0.944265574	0.619404434	0.947534536	0.577536289	0.947534536
GCST90032621	0.989627506	1.002201587	0.605441829	0.958703942	0.538557618	0.959038941	0.829108526	0.968623417	0.804698031	0.968623417
GCST90032622	0.9389692	1.027568997	0.086109992	1.34504742	0.042632028	1.291804534	0.247073788	1.408906076	0.247020603	1.402189721
GCST90032623	0.724923938	0.919634462	0.42303213	0.907757128	0.6090613	0.954918937	0.554353862	0.874816737	0.564896018	0.878462734
GCST90032624	0.27279355	1.352902825	0.154408244	1.219021509	0.481205323	1.073261668	0.354004472	1.288918368	0.310892603	1.304921446

GCST90032625	0.383182384	1.259580973	0.745512181	0.959274228	0.987121981	0.998401061	0.413223278	0.816375608	0.46848361	0.837959824
GCST90032626	0.945394053	1.022223683	0.552374121	0.907956709	0.977091141	0.996439393	0.441557016	0.774033146	0.548791174	0.805731728
GCST90032627	0.161164184	1.378461357	0.894416612	1.017353	0.801118512	1.023121959	0.883850974	1.03445698	0.817880481	1.054574556
GCST90032628	0.496528557	1.114046306	0.502366279	0.943036416	0.790312235	0.981198306	0.665017368	0.929992106	0.619504482	0.929992106
GCST90032629	0.523895271	1.095743518	0.749243179	0.974552524	0.483528609	1.041013307	0.459545529	0.891644405	0.426588781	0.894004614
GCST90032630	0.656298861	0.882915015	0.438565171	1.119129123	0.800680489	1.028178149	0.670058199	1.108469992	0.667130467	1.118824888
GCST90032631	0.021788451	0.704652312	0.41334996	0.912591669	0.55555915	0.95158957	0.542433991	0.884443798	0.55497147	0.889082637
GCST90032632	0.548282272	0.814041928	0.333752548	1.17375508	0.894016357	1.01968651	0.501968319	1.228385921	0.468817697	1.228385921
GCST90032633	0.770603584	1.070802634	0.620253955	1.069697985	0.786164914	1.028218806	0.821068446	1.065635802	0.759018322	1.085152301
GCST90032634	0.520343763	1.267509575	0.496895007	0.884725479	0.343988726	0.875809258	0.576002865	0.823775192	0.609992729	0.842171531
GCST90032635	0.946557287	1.022240154	0.217257471	1.191770779	0.171939316	1.17210203	0.201159445	1.465546712	0.22087596	1.40932314
GCST90032636	0.131957054	0.643449843	0.838053545	0.968395725	0.915676742	0.986156455	0.638497642	0.871812758	0.641814324	0.878099812
GCST90032637	0.386285113	1.41118367	0.876941516	0.974909302	0.529629285	0.915231155	0.601863779	1.211970588	0.631315018	1.160018821
GCST90032638	0.37430085	1.092766542	0.369198007	0.946083862	0.279605454	0.950038235	0.704149055	0.956725476	0.797570051	1.028562888
GCST90032639	0.23532293	1.528326003	0.314909701	1.203651276	0.802150889	1.03511975	0.321637637	1.423239752	0.294635661	1.403127545

	2									
GCST90032640	0.777958768	1.035303112	0.534542092	1.049943677	0.490757058	1.039618812	0.533403371	1.094897943	0.500574028	1.098533294
GCST90032641	0.518250678	0.898336879	0.280697023	1.073981643	0.943790558	1.004093749	0.387831423	1.106611773	0.342399413	1.109360303
GCST90032642	0.715565018	0.948270252	0.469819868	0.955745559	0.861143141	0.988633253	0.220362611	0.868323307	0.281295296	0.893910426
GCST90032643	0.301040327	0.781185164	0.010021419	0.819132542	0.061897881	0.870851572	0.135539509	0.797820362	0.118769034	0.799685376
GCST90032644	0.952389939	0.994663399	0.569152844	1.026660894	0.304083297	1.037798284	0.844049394	0.983542112	0.807672549	0.980432491

OR, odds ratio

TableS3 The reverse Mendelian randomization analysis results

Exposures		SNP(n)	OR	OR_lci95	OR_uci95	p-value
GCST90032182	Agathobacter sp000434275 abundance in stool	7	0.930080778	0.887429904	0.974781501	0.002474263
GCST90032551	Ruminococcus D abundance in stool	7	0.949753261	0.906416859	0.995161605	0.03049908
GCST90032550	Ruminococcus D bicirculans abundance in stool	7	0.95235567	0.908939641	0.997845492	0.040305902
GCST90032489	Negativibacillus sp000435195 abundance in stool	7	0.958808489	0.921987629	0.99709984	0.035259228
GCST90032457	Lactococcus lactis abundance in stool	7	0.97039663	0.943839985	0.997700494	0.033786301
GCST90032625	UBA7177 abundance in stool	7	0.974793997	0.955277195	0.994709537	0.01335841
GCST90032385	Faecalicatena sp002161355 abundance in stool	7	0.974815547	0.952390741	0.997768363	0.031701486
GCST90032433	Hungatella sp900155545 abundance in stool	7	0.977771493	0.960886786	0.994952899	0.011428092
GCST90032624	UBA7177 sp002491225 abundance in stool	7	0.980508812	0.962584617	0.998766773	0.036519745
GCST90032627	UBA7182 abundance in stool	7	0.981260954	0.964853551	0.997947367	0.027889507
GCST90032483	Morganella abundance in stool	7	0.981524801	0.964971248	0.99836232	0.031644716
GCST90032626	UBA7182 sp002491115 abundance in stool	7	0.982716204	0.969071474	0.996553054	0.014524292
GCST90032442	Kineothrix abundance in stool	7	0.98355838	0.96948664	0.997834365	0.024141136
GCST90032581	Thermoplasmatota abundance in stool	7	1.012685329	1.001855657	1.023632066	0.021563514
GCST90032566	Stappia abundance in stool	7	1.013964582	1.000784251	1.027318498	0.037761388
GCST90032357	Enorma massiliensis abundance in stool	7	1.023461934	1.000494207	1.046956917	0.045213047
GCST90032371	Eubacterium callanderi	7	1.024068889	1.004461592	1.044058925	0.015894233

	abundance in stool					
GCST90032304	CAG-841 sp002479075 abundance in stool	7	1.025310769	1.004067996	1.047002969	0.019280255
GCST90032295	CAG-698 abundance in stool	7	1.026696134	1.001032165	1.053018063	0.041362723
GCST90032524	Prevotella sp900318625 abundance in stool	7	1.028922096	1.000227899	1.058439462	0.048178454
GCST90032287	CAG-485 sp002404675 abundance in stool	7	1.029734305	1.005811542	1.05422606	0.014558512
GCST90032309	CAG-884 sp000433875 abundance in stool	7	1.033599053	1.000584378	1.067703061	0.04601315
GCST90032269	CAG-273 sp003507395 abundance in stool	7	1.053098848	1.003102164	1.10558747	0.037086355
GCST90032275	CAG-349 abundance in stool	7	1.08377499	1.018460839	1.153277754	0.011186728

SNP（n）, number of single nucleotide polymorphism; OR, odds ratio; lci95,95%lower confidence interval;uci95,95%upper confidence interval.

TableS4 Sensitivity analysis of reverse causality between intestinal flora and cervical spondylosis

gut microbiota		heterogeneity			mr_presso		pleiotropy	
		method	Q	Q_pval	mr_presso Outlier-corrected	mr_presso global test p val	mr_egger_intercept	p val
GCST90032182	Agathobacter sp000434275 abundance in stool	MR_egger	2.55302772	0.768488671	NA	0.861	-0.02007431	0.610225193
		IVW	2.848259257	0.827636772				
GCST90032551	Ruminococcus D abundance in stool	MR_egger	1.844843922	0.870170203	NA	0.934	0.001162564	0.975992602
		IVW	1.845844236	0.933316148				
GCST90032550	Ruminococcus D bicirculans abundance in stool	MR_egger	2.223433561	0.817444011	NA	0.909	-0.000694178	0.985649764
		IVW	2.223790877	0.898009093				
GCST90032489	Negativibacillus sp000435195 abundance in stool	MR_egger	1.427646832	0.921265286	NA	0.947	0.018830266	0.567912996
		IVW	1.80095445	0.937064463				
GCST90032457	Lactococcus lactis abundance in stool	MR_egger	3.727631098	0.589257467	NA	0.733	-0.0005079	0.982345139
		IVW	3.72817197	0.713405787				
GCST90032625	UBA7177 abundance in stool	MR_egger	6.207194535	0.286576926	NA	0.203	0.023246842	0.157688147
		IVW	9.630539091	0.141097997				
GCST90032385	Faecalicatena sp002161355 abundance in stool	MR_egger	1.661571798	0.893705047	NA	0.875	0.017298819	0.388316094
		IVW	2.55351	0.862431058				

			5065					
GCST900 32433	Hungatella sp900155545 abundance in stool	MR_egger	1.39387 4038	0.924982213	NA	0.97	-0.00051358	0.971 56693 2
		IVW	1.39527 7379	0.966145071				
GCST900 32624	UBA7177 sp002491225 abundance in stool	MR_egger	4.85615 1587	0.433686873	NA	0.181	0.024488753	0.090 68250 2
		IVW	9.23213 6094	0.160937634				
GCST900 32627	UBA7182 abundance in stool	MR_egger	2.23124 0367	0.816310879	NA	0.906	-0.002141615	0.878 11493 4
		IVW	2.25728 276	0.894581475				
GCST900 32483	Morganella abundance in stool	MR_egger	2.37882 3706	0.794623457	NA	0.843	-0.00653527	0.646 06646 6
		IVW	2.61715 8873	0.855132194				
GCST900 32626	UBA7182 sp002491115 abundance in stool	MR_egger	1.92325 5224	0.859658998	NA	0.887	0.009941985	0.407 68930 9
		IVW	2.73953 3889	0.840755743				
GCST900 32442	Kineothrix abundance in stool	MR_egger	4.27217 1081	0.510931688	NA	0.6	0.004117911	0.731 38891 4
		IVW	4.40399 8909	0.622177658				
GCST900 32581	Thermoplasmatot a abundance in stool	MR_egger	6.18265 8963	0.288849031	NA	0.41	-0.006806332	0.472 09385 2
		IVW	6.92992 2042	0.327376657				
GCST900 32566	Stappia abundance in stool	MR_egger	2.25305 8783	0.813135979	NA	0.529	0.018400409	0.133 99929 2
		IVW	5.44606 1431	0.487999738				

GCST90032357	Enorma massiliensis abundance in stool	MR_egger	4.767149876	0.444953331	NA	0.573	-0.008887614	0.639899702
		IVW	5.014716628	0.541926987				
GCST90032371	Eubacterium callanderi abundance in stool	MR_egger	4.652997693	0.459677368	NA	0.447	0.019235358	0.250366925
		IVW	6.342454009	0.385941086				
GCST90032304	CAG-841 sp002479075 abundance in stool	MR_egger	1.095654698	0.954487577	NA	0.687	0.029361113	0.134860298
		IVW	4.270908398	0.640067113				
GCST90032295	CAG-698 abundance in stool	MR_egger	4.593361247	0.46749002	NA	0.413	0.024730454	0.263344175
		IVW	6.180455041	0.403282177				
GCST90032524	Prevotella sp900318625 abundance in stool	MR_egger	5.101124349	0.403664012	NA	0.457	-0.020270871	0.408609425
		IVW	5.930402155	0.431031533				
GCST90032287	CAG-485 sp002404675 abundance in stool	MR_egger	1.224015994	0.94255271	NA	0.971	-0.009819927	0.618269328
		IVW	1.505780665	0.959109646				
GCST90032309	CAG-884 sp000433875 abundance in stool	MR_egger	4.142430949	0.529097096	NA	0.662	0.004831435	0.857446966
		IVW	4.178191695	0.65257639				
GCST90032269	CAG-273 sp003507395 abundance in	MR_egger	5.92143232	0.313939135	NA	0.467	-0.016133994	0.712206599

	stool							
		IVW	6.102081938	0.411852579				
GCST90032275	CAG-349 abundance in stool	MR_egger	8.0119455	0.155578515	NA	0.239	0.019076637	0.733127996

IVW,inverse variance weighted.
